# Supplementary material for: SIRPα ablated iPSC-derived macrophages resist hypophagia and enhance mAb-dependent and CAR-mediated cytotoxicity of solid tumors
Source: Mol Ther Oncol. 2026 May 20;34(2):201240. doi: 10.1016/j.omton.2026.201240 (PMC13264185; doi:10.1016/j.omton.2026.201240)
Supplement: Document S1. Figures S1–S7 and Tables S1–S3 [file mmc1.pdf]

OMTON, Volume *34*

Supplemental information

SIRPα ablated iPSC-derived macrophages resist hypophagia and enhance mAb-dependent and CAR-mediated cytotoxicity of solid tumors

Portia R. Smith, Md Ehsanul Kabir, Jue Zhang, John P. Maufort, Matthew H. Forsberg, Divine M. Sedzro, Mark Berres, James A. Thomson, Christian M. Capitini, and Igor I. Slukvin

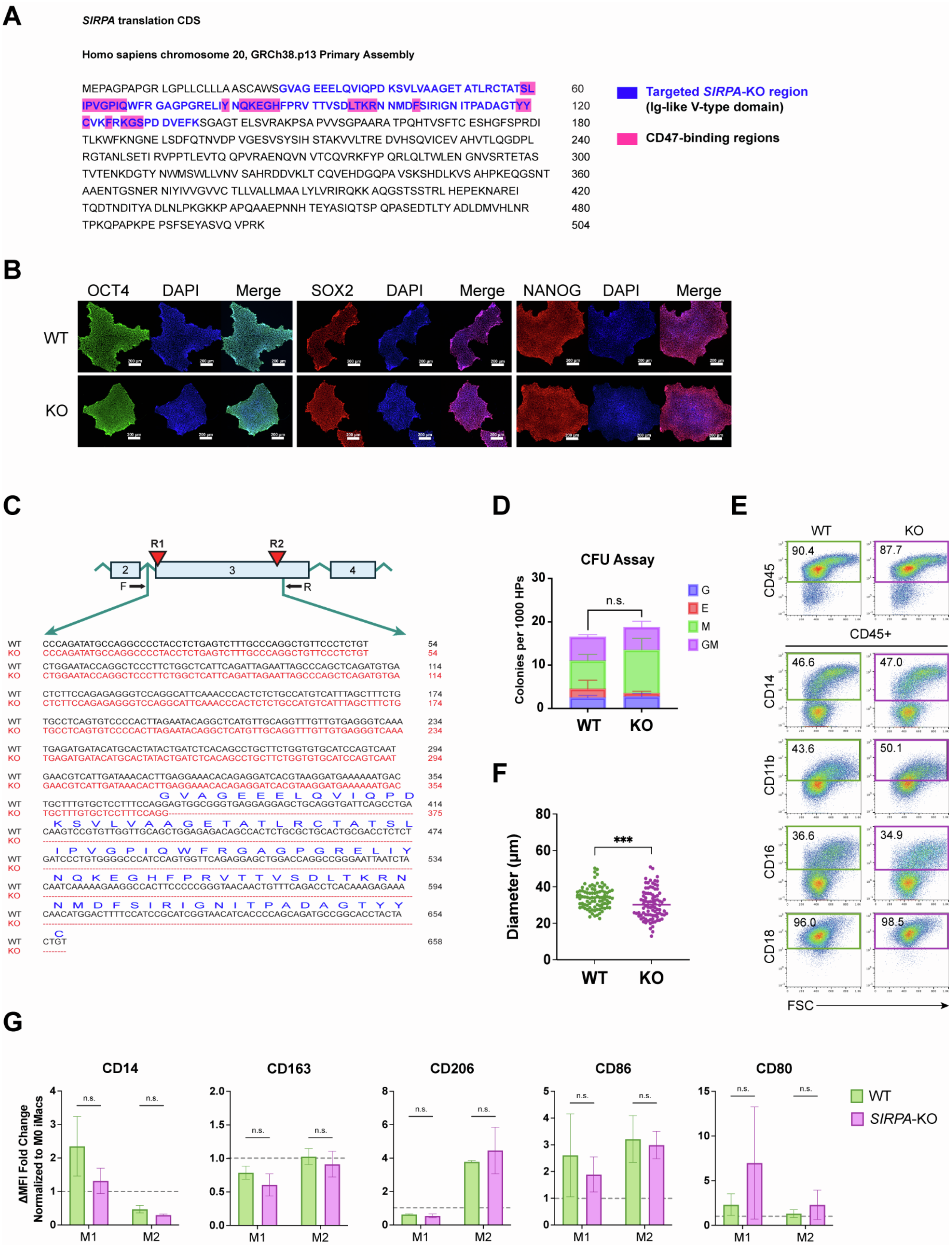
A
SIRPA translation CDS
Homo sapiens chromosome 20, GRCh38.p13 Primary Assembly
MEPAGPAPGR LGPLLCLLLA ASCAWSGVAG EEELQVIQPD KSVLVAAGET ATLRCTATSL 60
IPVGPIQWFR GAGPGRELIY NQKEGHFPRV TTVSDLTKRN NMDFSIRIGN ITPADAGTYY 120
CVKFRKGSPD DVEFKSGAGT ELSVRAKPSA PVVSGPAARA TPQHTVSFTC ESHGFSPRDI 180
TLKWFKNGNE LSDFQTNVDP VGESVSYSIH STAKVVLTRE DVHSQVICEV AHVTLQGDPL 240
RGTANLSETI RVPPTLEVTQ QPVRAENQVN VTCQVRKFYP QRLQLTWLEN GNVSRTETAS 300
TVTENKDGTY NWMSWLLVNV SAHRDDVKLT CQVEHDGQPA VSKSHDLKVS AHPKEQGSNT 360
AAENTGSNER NIYIVVGVVC TLLVALLMAA LYLVRIRQKK AQGSTSSTRL HEPEKNAREI 420
TQDTNDITYA DLNLPKGKKP APQAAEPNNH TEYASIQTSP QPASEDTLTY ADLDMVHLNR 480
TPKQPAPKPE PSFSEYASVQ VPRK 504
Targeted SIRPA-KO region
(Ig-like V-type domain)
CD47-binding regions
B
OCT4 DAPI Merge SOX2 DAPI Merge NANOG DAPI Merge
WT
KO
C
R1
R2
2
3
4
F
R
D
CFU Assay
Colonies per 1000 HPs
n.s.
G
E
M
GM
WT KO
E
WT KO
CD45
90.4
87.7
CD45+
CD14
46.6
47.0
CD11b
43.6
50.1
CD16
36.6
34.9
CD18
96.0
98.5
FSC
F
Diameter (μm)
***
WT KO
G
CD14
CD163
CD206
CD86
CD80
ΔMFI Fold Change
Normalized to M0 iMacs
n.s.
M1 M2
WT
SIRPA-KO

Figure S1. Characterization of SIRPA-KO iPSC line. (A) Human *SIRPA* gene translation CDS generated in Geneious Prime software. Amino acid sequence in blue text is the approximate knockout target region using two sgRNAs flanking *SIRPA* exon 3. Amino acids highlighted in pink represent CD47-binding motifs within SIRPα protein.[1,2] (B) Immunofluorescence of OCT4, SOX2, and NANOG expression on WT and *SIRPA*-KO (KO) iPSCs. (C) Nanopore sequencing of genomic PCR product from WT and *SIRPA*-KO iPSCs amplified with primers (black arrows) to target *SIRPA* exon 3 described in Table S2. Red triangles R1 and R2 indicate *SIRPA*-KO sgRNAs 1 and 2, respectively. Nucleotide sequence of WT iPSCs is denoted in black text, and SIRPA-KO iPSCs in red text. Predicted amino acid translation of WT exon 3 is denoted in large blue lettering. (D) Colony forming unit (CFU) assay from WT and *SIRPA*-KO iPSC-derived day 9 floating HPs. Data are represented as mean ± SEM (WT n=2; KO n=4) n.s. p>0.05, multiple unpaired t tests. (E) Flow cytometric analysis of myeloid cells after culture of day 9 floating HPs with M-CSF, IL-3 and IL-6 for 6 days. (F) WT and *SIRPA*-KO iPSC-derived macrophages (iMacs) were stained with Wright-Giemsa and imaged using brightfield microscopy. iMac diameters were measured as the maximum cell width (widest point) from brightfield images using Image J.JS analysis. Data are represented as mean and individual points (n=90 per group). ***p = 0.0004, Welch's t test. (G) Expression of CD14, CD163, CD206, CD86, and CD80 in WT and *SIRPA*-KO iMacs stimulated for 48 hours with IFN-γ + LPS (M1), IL-4 (M2), or unstimulated (M0) and unstained (Uns.) macrophages, which were used as a control. Data are represented as mean ± SEM (n=3), n.s. p>0.05, multiple paired t-tests.

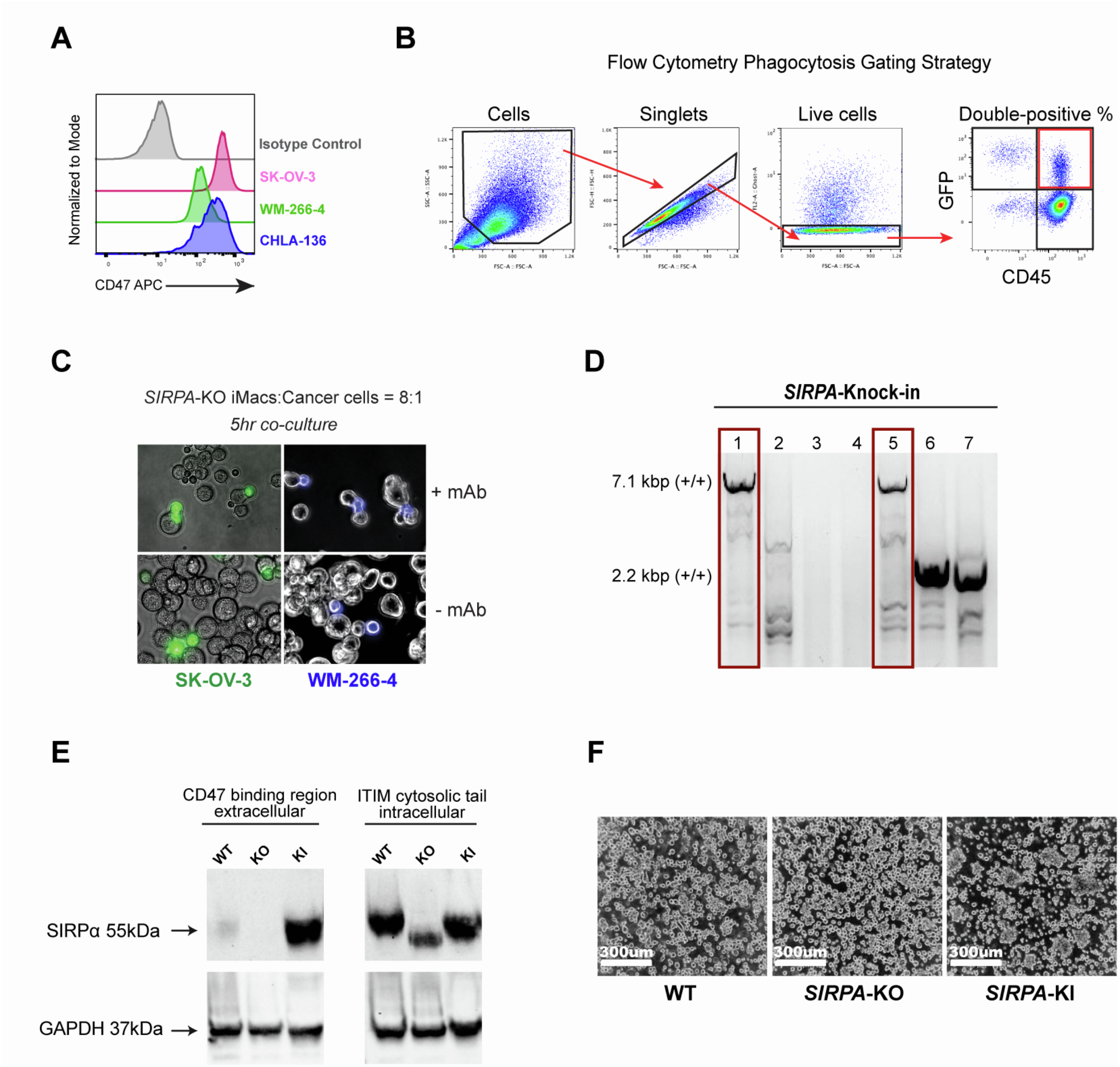

Figure S2. Characterization of tumor cell lines and *SIRPA* KO and Knock-in (KI) cell lines. (A) Flow cytometric analysis of human CD47 expression on cancer cell lines used in this study. **(B)** Representative flow cytometry gating schematic for antibody-dependent phagocytosis assay. Engulfment index was calculated as (# Double-positive GFP+CD45+ cells) / (Total # of GFP+ cells) x 100. **(C)** Fluorescence microscopy images of WT or *SIRPA*-KO iMacs co-cultured with various cancer cell lines with or without mAb; SK-OV-3 +/- anti-HER2, WM-266-4 +/- anti-GD3. **(D)** Genomic PCR of *SIRPA*-Knock-in within the AAVS1 locus of *SIRPA*-KO iPSCs. **(E)** Western blot of human SIRPα protein (55kDa) and GAPDH protein (37kDa) within WT, *SIRPA*-KO (KO), or *SIRPA*-KI (KI) iMacs. **(F)** Phase contrast microscopy images of WT, *SIRPA*-KO, or *SIRPA*-KI iMac cell cultures.

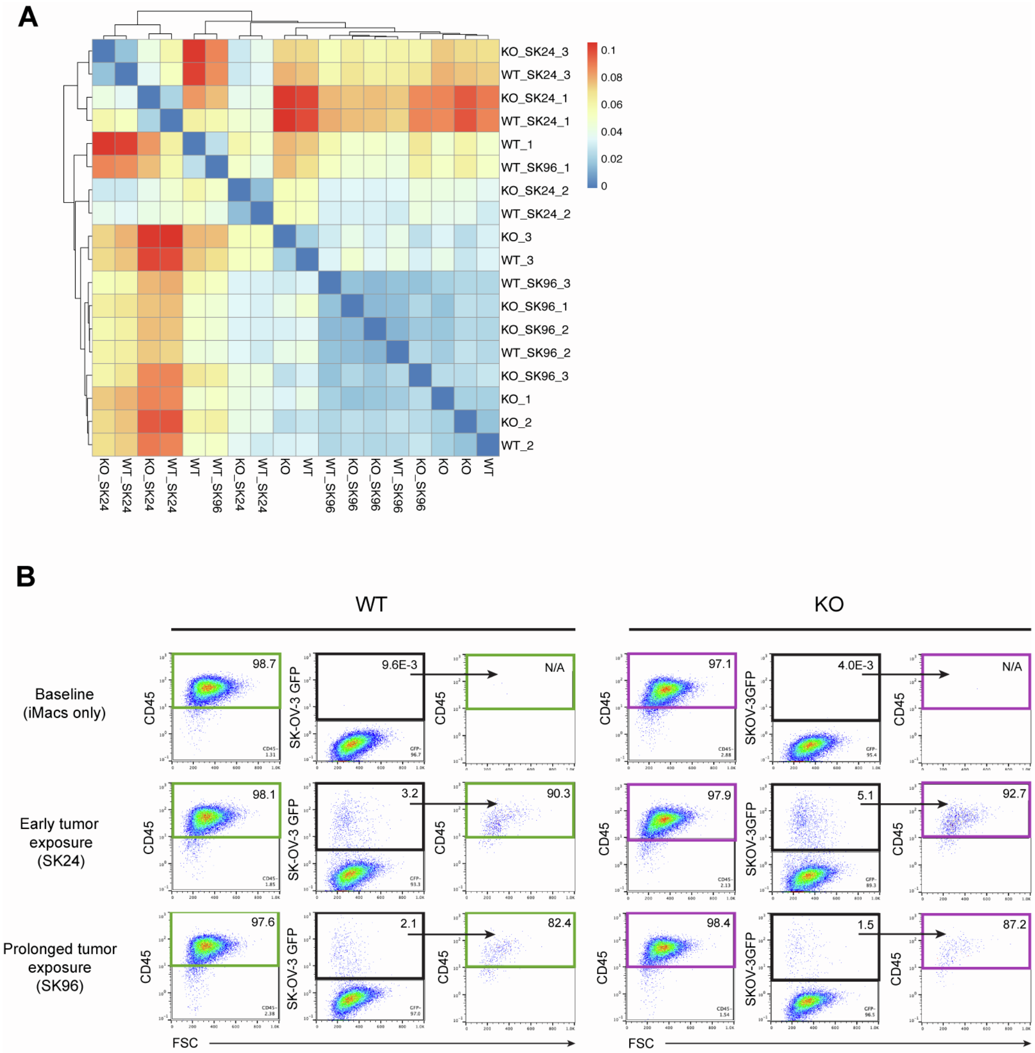

Figure S3. RNA-sequencing sample validation and quality control of WT and *SIRPA*-KO iMacs. **(A)** Sample level correlation heatmap of RNA-sequencing data with hierarchal clustering. Heatmap shows pairwise correlation differences (1 – correlation) between individual RNA-sequencing samples from WT or *SIRPA*-KO iMacs under baseline conditions, 'early' 24hr tumor exposure (SK24), or 'prolonged' 96hr tumor exposure (SK96). **(B)** Flow cytometric validation of CD45+ post-magnetic bead sorting of WT or *SIRPA*-KO iMacs under baseline conditions, 'early' 24hr tumor exposure (SK24), or 'prolonged' 96hr tumor exposure (SK96). Representative flow cytometry plots of each treatment group show CD45 and GFP expression after CD45+ magnetic bead sorting enrichment prior to total RNA isolation.

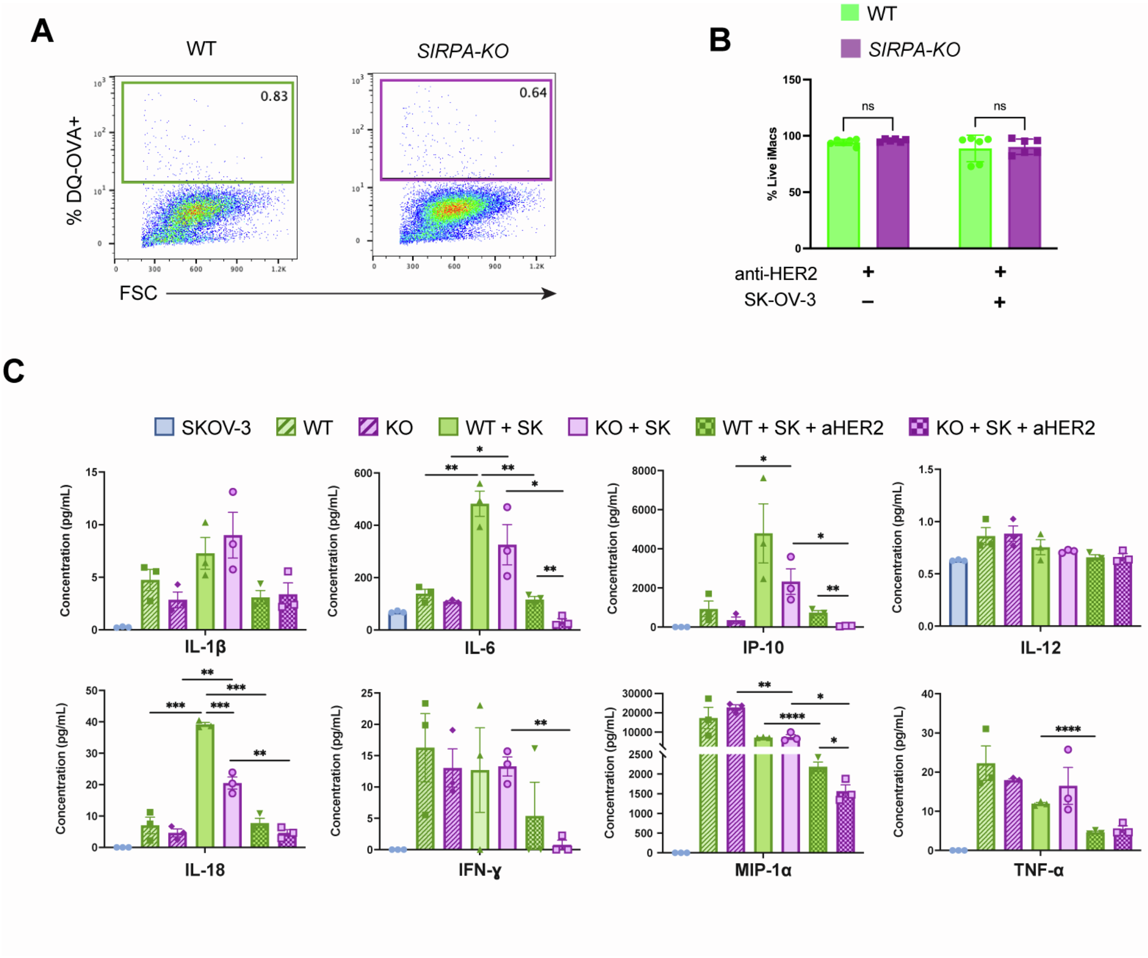

Figure S4. Characterization of *SIRPA*-KO iMacs after serial exposure to the tumor. (A) Representative dot plots show DQ-OVA fluorescence of iMacs from 96 hours serial tumor exposure following 30 minutes of exposure to DQ-OVA at 4^0C. **(B)** Viability of $CD45^+$gated iMacs from 96 hours serial tumor exposure cultures with and without the addition of anti-HER2 or SK-OV-3. **(C)** Secretome analysis of iMacs from 96 hours serial tumor exposure cultures. WT and *SIRPA*-KO iMacs were co-cultured with SK-OV-3 GFP-Luc2+ cancer cells +/- anti-HER2 at a 20:1 effector-to-target ratio. Every 24 hours, total media was replenished with fresh SK-OV-3 +/- anti-HER without disturbing the existing co-culture. At 96 hours, cell culture media of the co-cultures were collected for secretome analysis. Results are mean ± SEM (n=3); *p<0.05, **p<0.01, ***p<0.001, ****p<0.0001, student's t test.

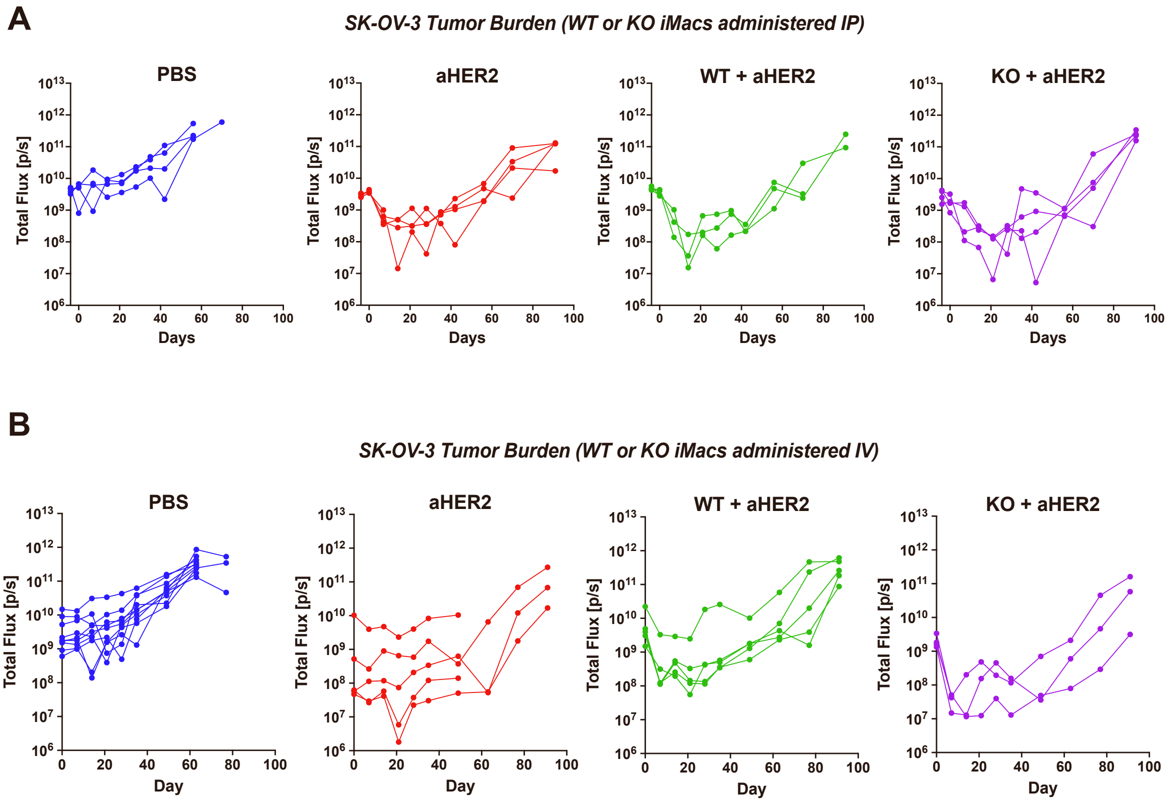

Figure S5. Quantification of tumor burden in individual mice. (A) Quantification of SK-OV-3 tumor xenografts over time for each treatment group after IP injection of iMacs. Results are total flux [photons/s] of tumor burden of each individual mouse from each group (n=4). **(B)** Quantification of SK-OV-3 tumor xenografts over time for each treatment group after IV injection of iMacs. Results are total flux [photons/s] of tumor burden of each individual mouse from each group (PBS n=8, anti-HER2 n=5, WT + aHER2 n=5, KO + aHER2 n=3).

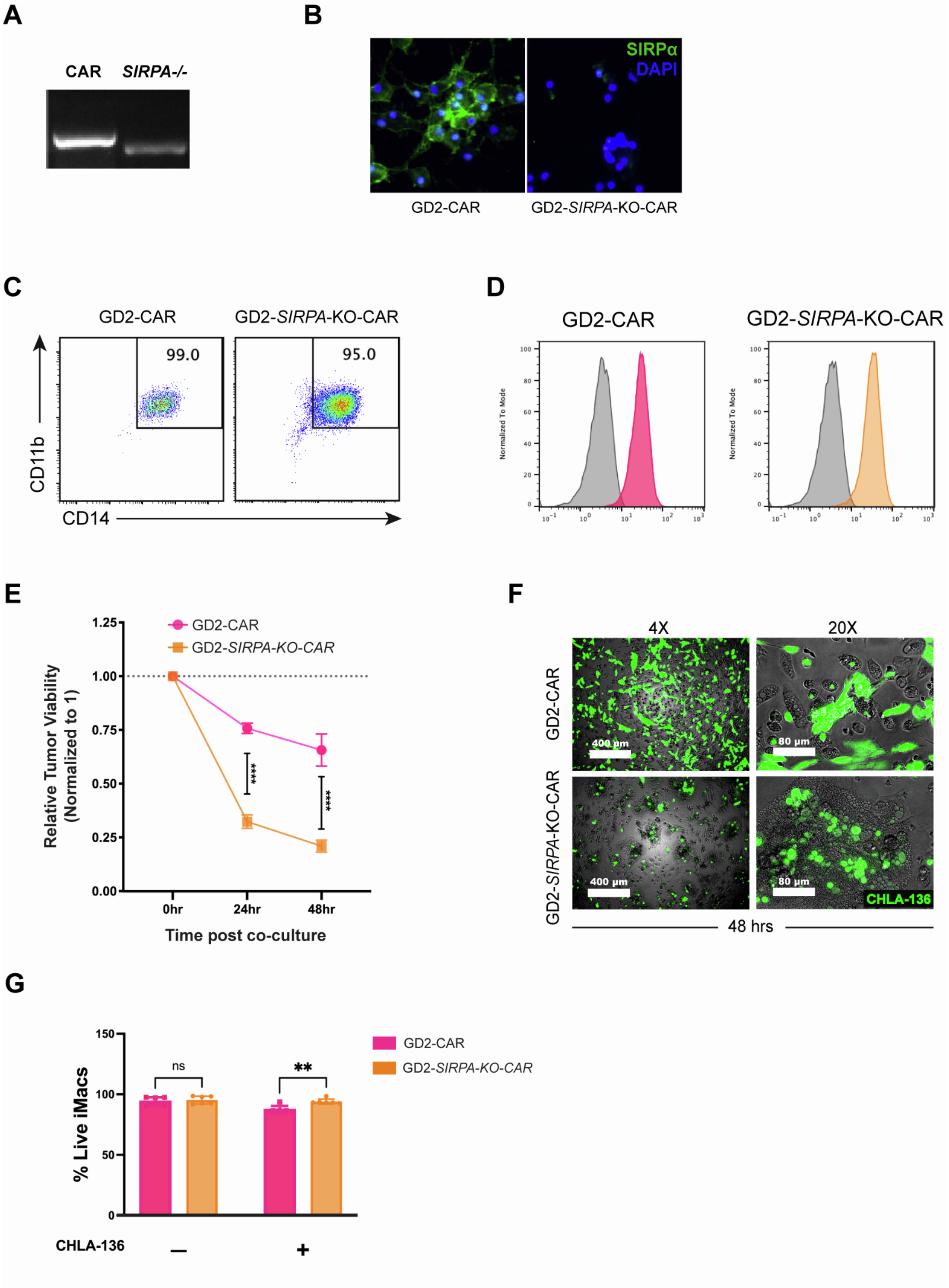

Figure S6. Characterization of the impact of *SIRPA* KO on GD2-CAR iMacs. (A) Genomic PCR of successful *SIRPA*-knockout in GD2-*SIRPA*-KO PBMC-3-1 iPSCs. **(B)** PBMC-3-1 GD2-CAR and GD2-

SIRPA-KO-CAR iMacs were stained for DAPI (blue) and anti-SIRPα antibody (green) and subject to fluorescence microscopy. **(C)** PBMC-3-1 GD2-CAR and GD2-*SIRPA*-KO-CAR iMacs were stained for CD14 and CD11b in flow cytometric analysis. **(D)** PBMC-3-1 WT (grey), GD2-CAR (pink) and GD2-*SIRPA*-KO-CAR (orange) iMacs were stained for an antibody that recognizes anti-GD2-CAR expression (1A7) in flow cytometric analysis. **(E)** IISH2i-BM9 CAR or GD2-*SIRPA*-KO iMacs were co-cultured with CHLA-136 GFP-Luc2+ neuroblastoma cells at 1:1 E:T ratio for 0, 24 or 48 hours. Results are shown as mean ± SEM (n=6); ****$p<0.0001$, two-way ANOVA. **(F)** Fluorescent microscopy images of GFP+ CHLA-136 viable cells during in vitro serial tumor exposure assay at 4X and 20X magnification. **(G)** Viability of CAR-iMacs in 96 hours serial tumor exposure cultures. Results are mean ± SE (n=6). **$p<0.01$.

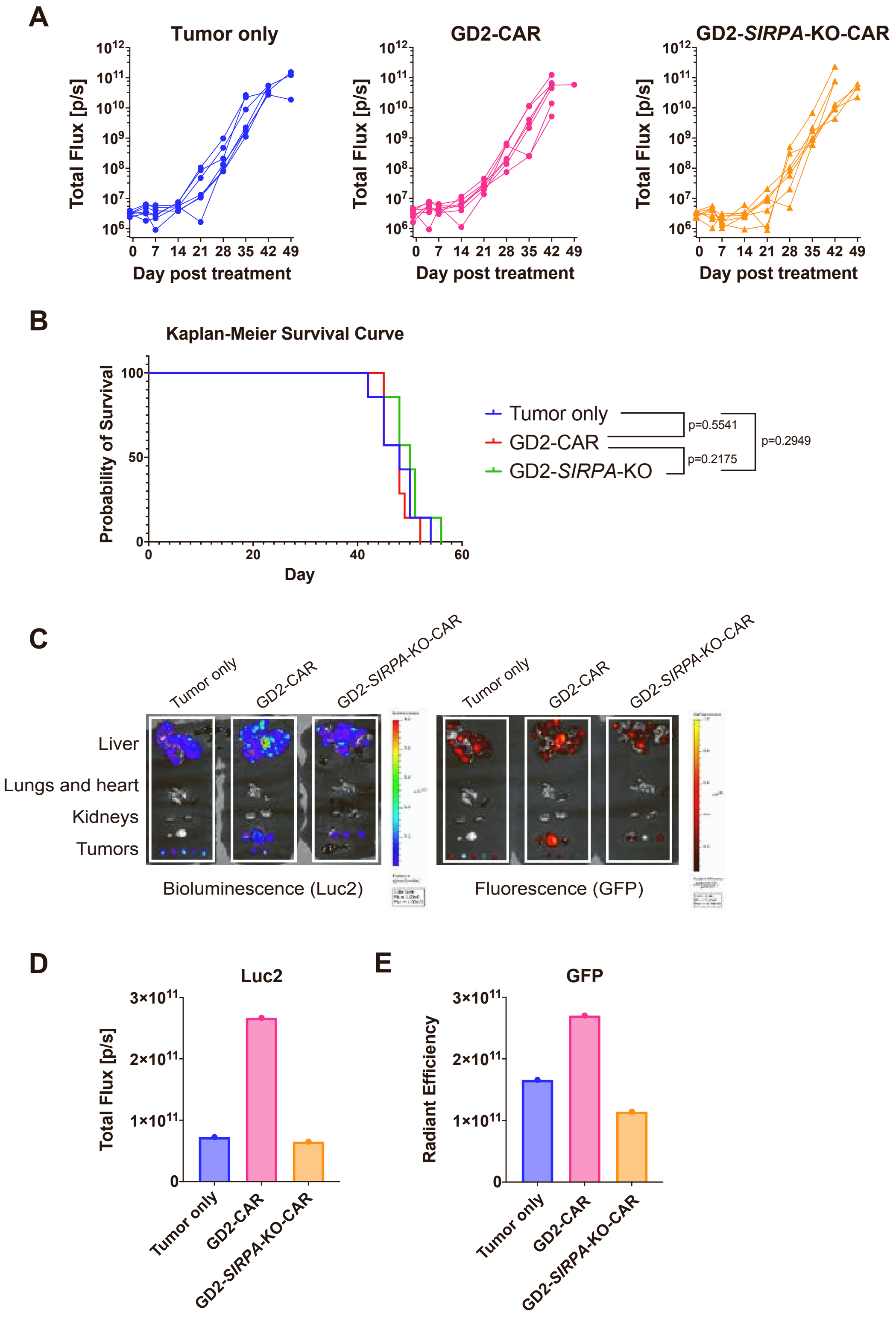
A
Tumor only
GD2-CAR
GD2-SIRPA-KO-CAR
Total Flux [p/s]
10^{6}
10^{7}
10^{8}
10^{9}
10^{10}
10^{11}
10^{12}
0 7 14 21 28 35 42 49
Day post treatment
B
Kaplan-Meier Survival Curve
Probability of Survival
100
50
0
0 20 40 60
Day
Tumor only
GD2-CAR
GD2-SIRPA-KO
p=0.5541
p=0.2175
p=0.2949
C
Tumor only
GD2-CAR
GD2-SIRPA-KO-CAR
Liver
Lungs and heart
Kidneys
Tumors
Bioluminescence (Luc2)
Fluorescence (GFP)
D
Luc2
Total Flux [p/s]
3×10^{11}
2×10^{11}
1×10^{11}
0
Tumor only
GD2-CAR
GD2-SIRPA-KO-CAR
E
GFP
Radiant Efficiency
3×10^{11}
2×10^{11}
1×10^{11}
0
Tumor only
GD2-CAR
GD2-SIRPA-KO-CAR

Figure S7. Evaluating GD2-*SIRPA*-KO-CAR iMacs in CHLA-136 xenograft model. (A) Quantification of CHLA-136 tumor xenografts over time for each treatment group, shown as individual mice. NC, negative control. Results are mean total flux [photons/s] ± SEM (n=7). (**B**) Kaplan-Meier Survival Curve of mice from Tumor only, GD2-CAR, and GD2-*SIRPA*-KO-CAR treatment groups using a log-rank test. **(C-E)** Results shown are representative of n=1 mouse from each group. **(C)** Representative images of GFP-Luc2+ CHLA-136 tumor metastasis in NCG-X mice. Organs were isolated upon end of survival on day 48 and imaged using IVIS Spectra and D-Luciferin substrate for bioluminescence (Luc2 expression) and fluorescence (GFP). **(D-E)** Bar graphs of **(D)** Luciferase (Luc2) Total Flux [p/s] and **(E)** GFP Radiant Efficiency of total organs, shown in white ROIs in **(C).**

SUPPLEMENTAL METHODS

SIRPA-KO iPSC PCR sequencing

Partial nanopore PCR sequence alignment of exon 3 of WT and SIRPA KO BM9 iPSCs. Genomic DNA was PCR-amplified, gel-purified, and sequenced using the Oxford Nanopore platform. The KO sequence is identical to WT up to the edited region, followed by a deletion of the downstream sequence.

WT sequence (exon 3)

CCCAGATATGCCAGGCCCCTACCTCTGAGTCTTTGCCCAGGCTGTTCCCTCTGTCTGGAATACC
AGGCTCCCTTCTGGCTCATTCAGATTAGAATTAGCCCAGCTCAGATGTGACTCTTCCAGAGAG
GGTCCAGGCATTCAAACCCACTCTCTGCCATGTCATTTAGCTTTCTGTGCCTCAGTGTCCCCAC
TTAGAATACAGGCTCATGTTGCAGGTTTGTTGTGAGGGTCAAATGAGATGATACATGCACTATA
CTGATCTCACAGCCTGCTTCTGGTGTGCATCCAGTCAATGAACGTCATTGATAAACACTTGAG
GAAACACAGAGGATCACGTAAGGATGAAAAAATGACTGCTTTGTGCTCCTTTCCAGGAGTGG
CGGGTGAGGAGGAGCTGCAGGTGATTCAGCCTGACAAGTCCGTGTTGGTTGCAGCTGGAGA
GACAGCCACTCTGCGCTGCACTGCGACCTCTCTGATCCCTGTGGGGCCCATCCAGTGGTTCAG
AGGAGCTGGACCAGGCCGGGAATTAATCTACAATCAAAAAGAAGGCCACTTCCCCCGGGTAA
CAACTGTTTCAGACCTCACAAAGAGAAACAACATGGACTTTTCCATCCGCATCGGTAACATCA
CCCCAGCAGATGCCGGCACCTACTACTGT

SIRPA-KO sequence (exon 3)

CCCAGATATGCCAGGCCCCTACCTCTGAGTCTTTGCCCAGGCTGTTCCCTCTGTCTGGAATACC
AGGCTCCCTTCTGGCTCATTCAGATTAGAATTAGCCCAGCTCAGATGTGACTCTTCCAGAGAG
GGTCCAGGCATTCAAACCCACTCTCTGCCATGTCATTTAGCTTTCTGTGCCTCAGTGTCCCCAC
TTAGAATACAGGCTCATGTTGCAGGTTTGTTGTGAGGGTCAAATGAGATGATACATGCACTATA
CTGATCTCACAGCCTGCTTCTGGTGTGCATCCAGTCAATGAACGTCATTGATAAACACTTGAG
GAAACACAGAGGATCACGTAAGGATGAAAAAATGACTGCTTTGTGCTCCTTTCCAGG

Cancer cell culture

SK-OV-3 cells were obtained from American Type Culture Collection (ATCC, Manassas, Virginia, U.S.) and sub-cultured according to ATCC recommendations using McCoy's 5A Medium (ATCC, 30-2007) supplemented with 10% Fetal Bovine Serum (FBS; R&D Systems #S12450). WM-266-4 cells were obtained by the Department of Dermatology of the University of Wisconsin-Madison and sub-cultured using McCoy's 5A Medium (ATCC, 30-2007) supplemented with 10% FBS. CHLA-136 cells were

obtained as a gift from the Children's Hospital of Los Angeles and sub-cultured using Iscove's Modified Dulbecco's Medium (IMDM, Gibco, 12200069) supplemented with 10% FBS.

CAR construct and generation of anti-GD2 CAR-iPSCs

GD2-OX40-CD28-ζ CAR sequence was kindly provided by Malcolm Brenner (Baylor College of Medicine),[3] was cloned into an AAVS1-DEST vector (Addgene 80490) and integrated into the AAVS1 locus of the PBMC-3-1 iPSC line using CRISPR-Cas9 as previously described.[4,5]

Flow cytometry

Cells (0.1-1 x 10^6) were resuspended in FACS buffer (PBS with 2% FBS and 0.1% sodium azide) with dilutions of antibodies and incubated at 4°C for 20 – 30 minutes. After washing, flow cytometric analysis was performed on a MACSQuant Analyzer 10 (Miltenyi Biotec). FlowJo software (Version 10, FlowJo LLC) was used for the data analysis. Antibodies used in this study are listed in Table S3.

Phagocytosis assays

iMacs were co-cultured with GFP-expressing cancer cells lines with or without 1μg/mL anti-HER2 or anti-GD3 mAbs, listed in Table S3, in IF9S media, then harvested and stained with anti-CD45 APC antibody (BD, 555483) for flow cytometric analysis. Cells were then washed with FACS buffer for 1-2 times and flow cytometric analysis was performed as above. Engulfment Index was calculated as: (# of DP CD45+GFP+) / (# of total GFP+ cells) x 100.

***In vitro* tumor cell growth assays**

iMacs were co-cultured with GFP- and luciferase-expressing cancer cell lines with or without mAb in respective cancer culture media. To visualize GFP+ cancer cell viability, co-cultures were subject to fluorescence microscopy (ECHO Revolve). For luciferase-based viability, D-luciferin substrate was added to co-cultures and bioluminescence was read at 562nm using a SpectraMax i3X plate reader, Molecular Devices). Untreated cancer cells were used as a spontaneous death control, cancer cells treated with lysis buffer was used as a maximum death control. Relative tumor growth, normalized to 1, was calculated as: (experimental – maximum cell death) / (spontaneous death - maximum cell death).

Serial challenges of iMacs with tumor

iMacs were co-cultured with unmodified or GFP+ Luc2+ cancer cells together in IF9S media (SK-OV-3) or IMDM media + 10% FBS (CHLA-136) at either 10:1 or 20:1 effector-to-target ratios in the presence of anti-HER2 mAb (2μg/mL) for SK-OV-3 or no antibody for CHLA-136. After 24 hours, cultures were assessed for 1) GFP+ tumor viability by fluorescence microscopy and 2) luciferase activity by adding D-luciferin potassium substrate (VivoGlo, Promega, PAP1041) to the co-culture and reading bioluminescence at 562nm on a SpectraMax i3X plate reader. Immediately afterward, the total media of the co-cultures was removed and replenished with 5000 fresh cancer cells and anti-HER2 (2μg /mL) in IF9S media (SK-OV-3) or 5000 fresh cancer cells (CHLA-136). After media replacement, co-cultures were placed back into a normoxic incubator for another 24 hours. This process was repeated three to four times, for a total of four to five tumor exposures over the course of 96 or 120 hours, depending on the specific assay. Expression of FcRs or GD2 CAR was evaluated by flow cytometry. To assess the impact of multiple tumor exposures on phagocytosis, iMacs were serially challenged with unmodified tumor cells four times. iMacs from 96 hour cultures were collected and incubated with GFP-Luc2 cancer cells for 2 hours, labeled with CD45 antibody, and assessed for phagocytosis by flow cytometry. Antigen uptake and processing by iMacs following multiple rounds of tumor exposure was assessed using a self-quenched conjugate of OVA (DQ™ Ovalbumin (D-12053); Molecular Probes) that exhibits fluorescence upon proteolytic degradation. Total iMacs were collected from 96-hour cultures, counted, replated into new 96 well flat-bottom plate and incubated with 100 μg/ml DQ-OVA for 30 mins at 37°C or 4°C (control for background fluorescence) in IF9S media. OVA proteolysis was evaluated by flow cytometry after gating for $CD45^+$ iMacs.

M1/M2 Polarization and Flow Cytometric Analysis

WT and *SIRPA*-KO iMacs were seeded in a 6-well tissue culture plate at 2 million iMacs/mL in IF9S media. For M1 polarization, 20ng/mL IFN-γ (Peprotech, 300-02) and 100ng/mL LPS (Sigma Aldrich, L4391-1MG) were added to the cell culture. For M2 polarization, 20ng/mL IL-4 (Peprotech, 200-04) was added to the cell culture. After 48 hours, M1-stimulated, M2-stimulated, and unstimulated iMacs were collected by incubating with 1X diluted TrypLE (TrypLE Select Enzyme 10X, no phenol red; Thermo Fisher Scientific, A1217701), followed by centrifugation and resuspension in FACS buffer. Cell cultures were then subject to antibody staining and flow cytometric analysis. Antibodies used in this study are listed in Table S3.

Secretome assay

Secretome assay was performed according to the manufacturer's instructions for a Meso Quickplex SQ 120 multiplex cytokine plate reader (U-PLEX assay, MesoScale Discovery).

Western Blot

For Western Blot experiments, iMacs were generated from iPSCs and harvested for analysis. The cells were lysed using Pierce IP lysis buffer with Pierce protease inhibitors (Thermo Fisher Scientific). Protein levels were quantified using the Pierce BCA Assay kit (Thermo Fisher Scientific) and normalized to 10µg of total protein prior to running on pre-cast 4-12% gradient SDS-PAGE gels and subsequent transfer to PVDF membranes using the Bio-Rad Trans-Blot Turbo System. The membrane was blocked with 5% Difco™ Skim Milk (BD, 232100) in TBST buffer (1%; Diluted from 10X TBS Bio-Rad #1706435 and Tween20 Bio-Rad #1610781) for human anti-SIRPα antibody at 55kDa (MyBioSource, MBS2026512, 3µg/mL) and anti-GAPDH at 37kDa (Santa Cruz Biotechnology, 1:5000) for probing. The membranes were incubated with primary antibodies overnight at 4ºC after blocking with mild agitation and were blotted with their corresponding HRP-linked secondary antibodies at room temperature for one hour. Primary and secondary antibodies were diluted in 1% milk TBST buffer. After probing, membranes were washed in 1% TBST buffer for 5 mins three times with mild agitation. Detection of protein on membrane was visualized using Pierce ECL Western Blotting Substrate (Thermo Fisher Scientific).

Immunostaining of pluripotent stem cell markers

The pluripotency markers of WT and *SIRPA*-KO iPSC cells were examined by immunofluorescence staining. The iPSC colonies were fixed with 4% paraformaldehyde (PFA) at room temperature (RT) for 15 minutes after being washed with PBS. The colonies were permeabilized with 0.1 Triton X in PBS for 10 minutes at RT after being washed three times with PBS. Cell colonies were blocked with 5% goat serum at room temperature for one hour after the three washes (1x PBS with 0.2% Tween 20). Following three rounds of washing with 1x PBS containing 0.2% Tween 20, the colonies were incubated for an overnight at 4°C using the primary antibodies OCT3/4 (sc-5279 Santa Cruz Biotechnology), SOX2 (#3579S Cell Signaling Technology), and NANOG (#4903S Cell Signaling Technology) at a 1:200 dilution in 1x PBS containing 1% goat serum. After three washes, the primary antibody was removed, and the colonies were incubated with secondary antibodies, goat anti-mouse IgG Alexa Fluor 488 (#A11001 Thermo Fisher Scientific) for OCT3/4 and goat anti-rabbit IgG Alexa Fluor 594 (#A11012 Thermo Fisher Scientific) for SOX2 and NANOG, at a dilution of 1:5000 in 1x PBS containing 1% goat serum for one hour at RT and put into the platform rocker. As a nuclear stain control, the colonies were stained with Hoechst #33342 (Thermo Fisher Scientific) at 1:1000 in 1x PBS for 10 minutes in the dark after three washes. Finally, a Nikon Eclipse Ti2 widefield fluorescent microscope was used to take pictures of the colonies after they had been washed three times with 1x PBS containing 0.2% Tween 20.

Immunostaining of SIRPα

For immunofluorescence experiments, GD2 CAR iMacs and GD2-*SIRPA*-KO CAR iMacs were generated from iPSCs and harvested for analysis. iMacs were then fixed in cold methanol for 10 mins at -20°C and then blocked and permeabilized with 2.5% donkey serum (Sigma-Aldrich D9663) and 0.2% Triton-X100

(Made from 100% Triton X-100; Sigma Aldrich X100) for 20 mins at room temperature. Human anti-SIRPα (MyBioSource, MBS2026512, 3μg/mL) was added to the iMacs and incubated for 2 hours at room temperature. Primary antibody was removed, and a secondary anti-Rabbit IgG Alexa Fluor 488 (Invitrogen, A-21206, 2μg/mL) was applied and incubated for 1 hour at room temperature. In between staining steps, iMacs were washed with phosphate buffered saline (PBS). DAPI was used as a nuclear stain control. Images were taken by Nikon eclipse Ti confocal microscope, cropped from original 20x images.

Generation and validation of *SIRPA*-KO-*AAVS1*-*SIRPA*-Knock-in (KI) hiPSC line

The AAVS1 donor plasmid (derived from Addgene #22075) is linearized at the cloning site, downstream of CAG promoter (Figure 2I). To obtain *SIRPA* complementary DNA (cDNA) from cells, high-quality RNA was isolated (Invitrogen, #2910133) from WT (BM9) iMacs, removing genomic DNA contamination, and synthesizing cDNA through reverse transcription (Qiagen #178027558). A small sequence overlapped with each end of the cloning site is added onto the *SIRPA* gene through PCR. The insert and the linearized vector, with overlapped sequences of 15 bp – 20 bp on both 5'- and 3'-end, respectively, are mixed in an appropriate ratio and incubated with recombinase Exnase (ABP Biosciences, #D017-02) at 50°C for 5 – 15 mins according to manufacturer's instructions. The product was transformed into competent cells, purified (IBI Scientific, #IB47171) and verified through sequencing (FASTA sequence attached). The AAVS1-CAG-*SIRPA* plasmid, sgRNA (GGGGCCACTAGGGACAGGAT) and Cas9 protein (PNA Bio #CP02) were co-transfected into singularized *SIRPA*-KO iPSCs using Lonza Amaxa and Human Stem Cell Nucleofector Starter Kit (Lonza, VPH-5002). Cells were selected with puromycin (0.5 mg/mL, Sigma) for a maximum of 5 days and resistant clones were screened by PCR and Western Blot experiment.

RNA Sequencing

WT and *SIRPA*-KO iMacs were either cultured alone for 24 hours, or co-cultured with SK-OV-3 cells (E:T = 10:1) and anti-HER2 (1μg/mL) for 24 hours or 96 hours. All cells were harvested and sorted for CD45+ cells using anti-human CD45 antibody and anti-CD45 microbeads (Miltenyi, MACS sorter). Total RNA was isolated from the CD45+ sorted populations by using the RNeasy Mini Kit (Qiagen, 74104) and quantified with TapeStation (GENEWIZ by Azenta). The RNA samples were rRNA depleted using QIAGEN FastSelect rRNA HMR Kit (Qiagen, Germantown, MD, USA). RNA sequencing was performed at Azenta Life Sciences. The kit NEBNext® Ultra™ II RNA Library Prep Kit for Illumina® (New England Biolabs, Ipswich, MA, USA) was used for library preparation following manufacture instructions. For each sample ~20 million paired end reads per sample were sequenced on Illumina NovaSeq Xplus.

Bioinformatic analysis of RNA-sequencing data

Bioinformatic analysis of transcriptomic data adhered to recommended ENCODE guidelines and best practices for RNA-Seq.[6] Alignment of adapter-trimmed (Skewer v0.1.123)[7] 2x150 (paired-end; PE) bp strand-specific Illumina reads to the *Homo sapiens* GRCh38.p10 genome (assembly accession NCBI:GCA_000001405.25) was achieved with the Spliced Transcripts Alignment to a Reference (STAR v2.7.10b) software,[8] a splice-junction aware aligner, using annotation provided by Ensembl. Expression estimation was performed with RSEM v1.3.1 (RNASeq by Expectation Maximization).[9] To test for differential gene expression among individual group contrasts, expected read counts obtained from RSEM were used as input into edgeR (3.42.2).[10] Inter-sample normalization was achieved with the trimmed mean of M-values (TMM)[11] method. Statistical significance of the negative-binomial regression test was adjusted with a Benjamini-Hochberg false discovery rate (FDR) correction at the 5% level.[12] Prior to statistical analysis with edgeR, independent filtering was applied and required genes to have a count-per-million (CPM) above k in n samples, where k is determined by minimum read count (10 reads) and by the sample library sizes where n is determined by the number of biological replicates in each group. The validity of the Benjamini-Hochberg FDR multiple testing procedure was evaluated by inspection of the uncorrected p-value distribution. Gene set enrichment analysis (GSEA) was performed following criteria outlined by Subramanian et al., 2005.[13]

Table S1. Medium components in IF9S media.

Medium Components	Day 0-2	Day 2-4	Day 4-9	Day 9-15	Day 15-19	Vendor	Catalog #
IF9S*	x	x	x	x	x	N/A	N/A
FGF2 (50ng/mL)	x	x	x			Peprotech	100-18B
BMP4 (50ng/mL)	x					Peprotech	120-05
Activin A (15ng/mL)	x					Peprotech	120-14E
LiCl (2mM)	x					Sigma-Aldrich	203637-10G
Y-27632 (10µM)	x					Tocris	1254
VEGF (50ng/mL)		x	x			Peprotech	100-20
SB431542 (5µM)		x				Biogems	3014193
SCF (50ng/mL)			x			Peprotech	300-07
TPO (50ng/mL)			x			Peprotech	300-18
IL-3 (10ng/mL)			x	x		Peprotech	200-03
IL-6 (50ng/mL)			x	x		Peprotech	200-06
M-CSF (80ng/mL)				x	x	Peprotech	300-25

*IF9S medium components are previously defined by Uenishi et al., 2014.[14]

Table S2. PCR primers and synthetic guide (sg) RNAs used in this study.

**Name**	**Primer or sgRNA**	**Sequence**
SIRPA intron 2_F	primer	AATCTTAACACCTTGTACAGCCCCA
SIRPA exon 3_R	primer	AGTGCCTGCTCCAGACTTAAA
SIRPA sgRNA_1	sgRNA	GTGCTCCTTTCCAGGAGTGG
SIRPA sgRNA_2	sgRNA	ACTTAAACTCCACGTCATCG

Table S3. Antibodies used in this study.

Antibody Target	Conjugate	Vendor	Catalog #	Application
CD11b	APC	Miltenyi	130-091-241	Flow cytometry
CD11b	PE-Cy5	BD Biosciences	555389	Flow cytometry
CD14	PE	BD Biosciences	555398	Flow cytometry
CD16	PE	BD Biosciences	555407	Flow cytometry
CD18	APC	BD	551060	Flow cytometry
CD32	APC	BD Biosciences	559769	Flow cytometry
CD41a	PE	BD Biosciences	555467	Flow cytometry
CD43	FITC	BD Biosciences	555475	Flow cytometry
CD45	APC	BD Biosciences	555485	Flow cytometry
CD45	BV421	BD Biosciences	563880	Flow cytometry
CD47	APC	Biolegend	323123	Flow cytometry
CD47	PE	Biolegend	323108	Flow cytometry
CD64	FITC	BD Biosciences	555527	Flow cytometry
CD80	PE-Cy5	BD Biosciences	559370	Flow cytometry
CD86	APC	Miltenyi	130-116-161	Flow cytometry
CD163	BV421	Biolegend	333610	Flow cytometry
CD206	FITC	Biolegend	321104	Flow cytometry
CD235a	PE	BD Biosciences	561051	Flow cytometry
HLA-DR	APC	BD Biosciences	559868	Flow cytometry
Anti-14G2a [1A7] *	APC	NCI Repository	NA	Flow cytometry
Anti-GD3 (R24)	Unconjugated	NCI Repository	NA	Functional studies
Anti-GD2 (Ch14.18)	Unconjugated	NCI Repository	NA	Functional studies
Herceptin (anti-HER2)	Unconjugated	Genetech, Inc	NA	Functional studies
SIRPα	Unconjugated	MyBioSource	MBS2026512	Western blot

*Anti-14G2a [1A7] APC-conjugated antibody was generated in house using unconjugated Anti-14G2a [1A7] antibody and the Abcam APC Conjugation Kit – Lightning Link #ab201807

REFERENCES

1. Hatherley, D., Graham, S.C., Turner, J., Harlos, K., Stuart, D.I., and Barclay, A.N. (2008). Paired Receptor Specificity Explained by Structures of Signal Regulatory Proteins Alone and Complexed with CD47. Molecular Cell *31*, 266–277. https://doi.org/10.1016/j.molcel.2008.05.026.

2. Hatherley, D., Harlos, K., Dunlop, D.C., Stuart, D.I., and Barclay, A.N. (2007). The Structure of the Macrophage Signal Regulatory Protein α (SIRPα) Inhibitory Receptor Reveals a Binding Face Reminiscent of That Used by T Cell Receptors*. Journal of Biological Chemistry *282*, 14567–14575. https://doi.org/10.1074/jbc.M611511200.

3. Louis, C.U., Savoldo, B., Dotti, G., Pule, M., Yvon, E., Myers, G.D., Rossig, C., Russell, H.V., Diouf, O., Liu, E., et al. (2011). Antitumor activity and long-term fate of chimeric antigen receptor-positive T cells in patients with neuroblastoma. Blood *118*, 6050–6056. https://doi.org/10.1182/blood-2011-05-354449.

4. Oceguera-Yanez, F., Kim, S.-I., Matsumoto, T., Tan, G.W., Xiang, L., Hatani, T., Kondo, T., Ikeya, M., Yoshida, Y., Inoue, H., et al. (2016). Engineering the AAVS1 locus for consistent and scalable transgene expression in human iPSCs and their differentiated derivatives. Methods *101*, 43–55. https://doi.org/10.1016/j.ymeth.2015.12.012.

5. Zhang, J., Webster, S., Duffin, B., Bernstein, M.N., Steill, J., Swanson, S., Forsberg, M.H., Bolin, J., Brown, M.E., Majumder, A., et al. (2023). Generation of anti-GD2 CAR macrophages from human pluripotent stem cells for cancer immunotherapies. Stem Cell Reports *18*, 585–596. https://doi.org/10.1016/j.stemcr.2022.12.012.

6. Hitz, B.C., Rowe, L.D., Podduturi, N.R., Glick, D.I., Baymuradov, U.K., Malladi, V.S., Chan, E.T., Davidson, J.M., Gabdank, I., Narayanan, A.K., et al. (2016). SnoVault and encodeD: A novel object-based storage system and applications to ENCODE metadata. Preprint, https://doi.org/10.1101/044578 https://doi.org/10.1101/044578.

7. Jiang, H., Lei, R., Ding, S.-W., and Zhu, S. (2014). Skewer: a fast and accurate adapter trimmer for next-generation sequencing paired-end reads. BMC Bioinformatics *15*, 182. https://doi.org/10.1186/1471-2105-15-182.

8. Dobin, A., Davis, C.A., Schlesinger, F., Drenkow, J., Zaleski, C., Jha, S., Batut, P., Chaisson, M., and Gingeras, T.R. (2013). STAR: ultrafast universal RNA-seq aligner. Bioinformatics *29*, 15–21. https://doi.org/10.1093/bioinformatics/bts635.

9. Li, B., and Dewey, C.N. (2011). RSEM: accurate transcript quantification from RNA-Seq data with or without a reference genome. BMC Bioinformatics *12*, 323. https://doi.org/10.1186/1471-2105-12-323.

10. Robinson, M.D., McCarthy, D.J., and Smyth, G.K. (2010). edgeR: a Bioconductor package for differential expression analysis of digital gene expression data. Bioinformatics *26*, 139–140. https://doi.org/10.1093/bioinformatics/btp616.

11. Robinson, M.D., and Oshlack, A. (2010). A scaling normalization method for differential expression analysis of RNA-seq data. Genome Biology *11*, R25. https://doi.org/10.1186/gb-2010-11-3-r25.

12. Reiner, A., Yekutieli, D., and Benjamini, Y. (2003). Identifying differentially expressed genes using false discovery rate controlling procedures. Bioinformatics *19*, 368–375. https://doi.org/10.1093/bioinformatics/btf877.

13. Subramanian, A., Tamayo, P., Mootha, V.K., Mukherjee, S., Ebert, B.L., Gillette, M.A., Paulovich, A., Pomeroy, S.L., Golub, T.R., Lander, E.S., et al. (2005). Gene set enrichment analysis: A knowledge-based approach for interpreting genome-wide expression profiles. Proceedings of the National Academy of Sciences *102*, 15545–15550. https://doi.org/10.1073/pnas.0506580102.

14. Uenishi, G., Theisen, D., Lee, J.-H., Kumar, A., Raymond, M., Vodyanik, M., Swanson, S., Stewart, R., Thomson, J., and Slukvin, I. (2014). Tenascin C Promotes Hematoendothelial Development and T Lymphoid Commitment from Human Pluripotent Stem Cells in Chemically Defined Conditions. Stem Cell Reports *3*, 1073–1084. https://doi.org/10.1016/j.stemcr.2014.09.014.
