# Supplementary material for: SIRPα ablated iPSC-derived macrophages resist hypophagia and enhance mAb-dependent and CAR-mediated cytotoxicity of solid tumors
Source: Mol Ther Oncol. 2026 May 20;34(2):201240. doi: 10.1016/j.omton.2026.201240 (PMC13264185; doi:10.1016/j.omton.2026.201240)
Supplement: Document S2. Article plus supplemental information [file mmc2.pdf]

Molecular Therapy
Oncology
Original Article

SIRPα ablated iPSC-derived macrophages resist hypophagia and enhance mAb-dependent and CAR-mediated cytotoxicity of solid tumors

Portia R. Smith,[1] Md Ehsanul Kabir,[1,2] Jue Zhang,[3] John P. Maufort,[1,3] Matthew H. Forsberg,[4,5] Divine M. Sedzro,[1] Mark Berres,[6] James A. Thomson,[3] Christian M. Capitini,[4,5] and Igor I. Slukvin[1,2,5,7]

[1]Wisconsin National Primate Research Center, University of Wisconsin, Madison, WI 53715, USA; [2]Department of Pathology and Laboratory Medicine, University of Wisconsin School of Medicine and Public Health, Madison, WI 53792, USA; [3]Morgridge Institute for Research, Madison, WI 53715, USA; [4]Department of Pediatrics, University of Wisconsin School of Medicine and Public Health, Madison, WI 53792, USA; [5]Carbone Cancer Center, University of Wisconsin School of Medicine and Public Health, Madison, WI 53792, USA; [6]Bioinformatics Resource Center, University of Wisconsin, Madison, WI 53715, USA; [7]Department of Cell and Regenerative Biology, School of Medicine and Public Health, University of Wisconsin, Madison, WI 53707, USA

The SIRPα-CD47 "don't eat me" checkpoint axis plays a critical role in shaping antitumor activities of macrophages within the tumor microenvironment (TME). However, targeting this axis with anti-CD47 antibodies to enhance antitumor responses in clinical trials has been challenging. Here, we demonstrated that *SIRPA*-knockout (KO) iPSC-derived macrophages (iMacs) exhibit superior antitumor activity against various CD47-expressing tumors *in vitro* when combined with cancer-targeted monoclonal antibodies (mAbs) or chimeric antigen receptors (CARs). Moreover, *SIRPA*-KO protected macrophages from mAb- and CAR-driven hypophagia, enabling efficient tumoricidal effects even after serial tumor exposures. Retention of phagocytic activities in *SIRPA*-KO iMacs was associated with heightened surface expression of Fc receptors and GD2-CAR compared to their *SIRPA*-expressing counterparts. Despite the powerful impact of *SIRPA*-KO on iMac antitumor activities *in vitro*, only modest efficacy was observed in human xenograft mouse models of SK-OV3 ovarian carcinoma and CHLA-163 neuroblastoma treated with mAb or CAR-iMac therapy, indicating further engineering or combinatorial therapeutic strategies are needed for potent *in vivo* antitumor efficacy. Together, these findings identify SIRPα as a regulator in macrophage hypophagia and underscore the advantages of using *SIRPA*-KO macrophage therapeutic strategies to modulate the SIRPα-CD47 checkpoint to unleash macrophage antitumor activity within the TME.

INTRODUCTION

Macrophages represent the most abundant innate immune cells within the tumor microenvironment (TME). While tumor-associated macrophages (TAMs) have the potential to contribute to antitumor immunity, they often play an adverse role in solid tumors by promoting tumor growth, stimulating angiogenesis, suppressing antitumor activity from neighboring immune cells, and facilitating metastasis.[1,2] A key mechanism cancer cells use to exploit macrophages within the TME is the overexpression of the "don't eat me" receptor CD47, recognized by the myeloid-specific ligand signal regulatory protein alpha (SIRPα). Under physiological conditions, CD47 is ubiquitously expressed by healthy cells to inhibit unnecessary phagocytosis from macrophages by inhibiting cytoskeletal rearrangement necessary for phagocytosis.[3–8] By exploiting this mechanism, CD47-expressing tumors effectively block macrophage-driven phagocytosis, even in the presence of pro-phagocytic signals such as cancer-opsonizing antibodies or cancer "eat me" signals such as calreticulin.[9] Overexpression of CD47 has been previously linked to poor prognosis in numerous hematological cancers,[10] as well as solid tumors, including breast,[11] ovarian,[12] endometrial,[13] gastric,[14,15] non-small cell lung cancer,[16] and clear cell renal carcinoma.[17] As a result, CD47 holds significant therapeutic potential across a wide range of malignancies.

Blockade of CD47 and SIRPα has shown great efficacy against blood cancers but often requires dual-antibody blockade to achieve substantial tumor-killing effects.[18–23] CD47 blockade can also lead to extensive depletion of red blood cells (RBCs), potentially causing severe anemia in patients.[19,20,23] Furthermore, due to the intricate network of molecular interactions that CD47 participates in, including the high-affinity binding to thrombospondin-1 (TSP) and *cis* and *trans* binding partner interactions with numerous cell surface receptors, targeting CD47 therapeutically has proven challenging.[24,25] Thus, genetically engineered cellular therapies that disrupt the CD47/SIRPα axis offer an appealing alternative to antibody-based therapeutic approaches.

Received 11 December 2025; accepted 15 May 2026;
https://doi.org/10.1016/j.omton.2026.201240.

Correspondence: Dr. Igor I. Slukvin, Department of Pathology and Laboratory Medicine, Wisconsin National Primate Research Center, University of Wisconsin, 1220 Capitol Court, Madison, WI 53715, USA.
E-mail: islukvin@wisc.edu

Published by Elsevier Inc. on behalf of The American Society of Gene and Cell Therapy.
This is an open access article under the CC BY license (http://creativecommons.org/licenses/by/4.0/).

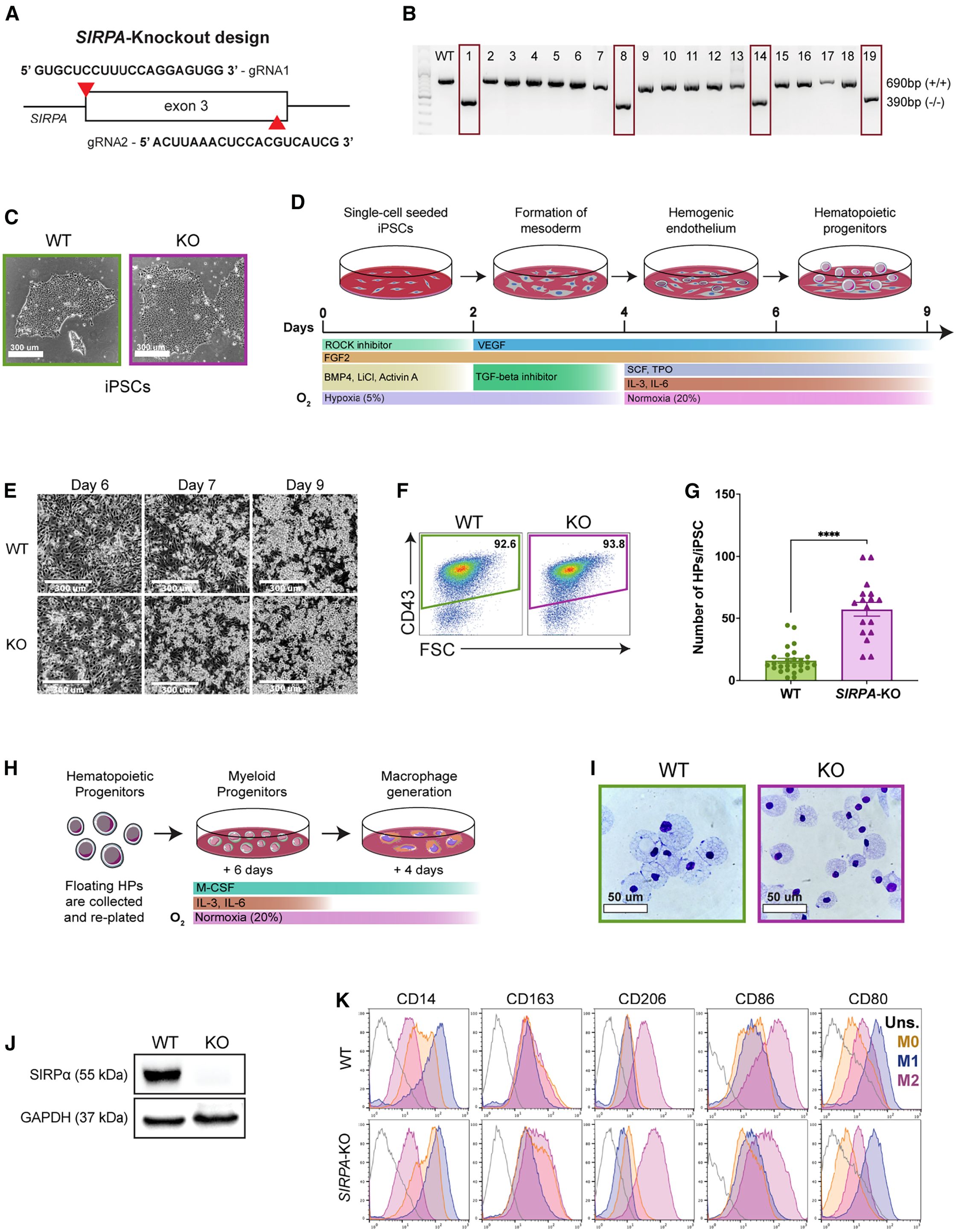

Figure 1. Knocking out *SIRPA* in human iPSCs and subsequent differentiation into macrophages

(A) Schematic for *SIRPA* gene knockout targeting exon 3.

(B) Genomic PCR analysis of iPSC clones collected from RNP-transfected cultures after targeting *SIRPA*.

(C) *SIRPA*-knockout (KO) iPSCs retain typical pluripotent stem cell morphology.

(D) Schematic for generating hematopoietic progenitors (HPs) from iPSCs using IF9S media and supplementation of various cytokines for 9 days.

(E) Phase contrast microscopy images of WT and KO cell cultures undergoing endothelial-to-hematopoietic transition and HP formation during days 6–9 of culture.

(legend continued on next page)

Macrophages are a promising immunotherapy platform given their unique ability to infiltrate tumors, modulate the tumor microenvironment, and be produced off-the-shelf for allogeneic use without risk of causing graft-versus-host disease to the recipient. Recently, our group and others have demonstrated the feasibility of targeting solid tumors with induced pluripotent stem cell (iPSC)-derived macrophages (iMacs) expressing chimeric antigen receptors (CARs).[26–28] However, these studies revealed only modest tumor reduction by CAR macrophages (CAR-Ms) during *in vivo* challenges. Given the importance of the "don't eat me" CD47-SIRPα pathway in suppressing macrophage-driven phagocytosis, we sought to investigate the antitumor efficacy of ablating SIRPα in iMacs in conjunction with pro-phagocytic molecules.

To this end, we utilized human iPSCs as a clonal and renewable source to generate macrophages with uniform multiplex gene edits. First, we demonstrated that iMacs with *SIRPA*-knockout (KO) exhibit superior *in vitro* antitumor activity and resist mAb-driven hypophagia-related exhaustion against CD47-expressing solid tumors, which was associated with better retention of Fc receptors as compared to WT iMacs. Furthermore, we showed that even a single injection of *SIRPA*-KO iMacs administered with anti-HER2 showed tumor retardation and significantly improved the survival of human ovarian cancer xenograft mice. When we combined *SIRPA*-KO with knockin of an anti-GD2-CAR, we revealed anti-GD2-*SIRPA*-KO-CAR-iMacs possess substantially improved antitumor responses against GD2-expressing solid tumor cell lines *in vitro* by alleviating CAR down-regulation and resisting CAR-mediated exhaustion during serial tumor exposure. Furthermore, our *in vitro* studies revealed that anti-GD2-CAR iMacs alone promote tumor growth, underscoring the necessity for *SIRPA*-KO and inhibition of pro-tumoral pathways in CAR macrophages. Additionally, during an *in vivo* neuroblastoma tumor challenge, we found anti-GD2-*SIRPA*-KO-CAR-iMacs significantly reduced tumor burden upon initial treatment compared to anti-GD2-CAR iMacs. Thus, in this study, we have demonstrated *SIRPA*-KO significantly improves the antitumor activity and prevents hypophagia-related exhaustion of iMacs against solid tumors in both mAb-driven and CAR-mediated contexts. Moreover, we have identified SIRPα as a key regulator of macrophage hypophagia and have established a multiplex genetic editing approach for the "off-the-shelf" generation of potent CAR iMac cellular therapy with limited pro-tumoral effects for treatment of CD47-expressing solid tumors.

RESULTS

Generation of *SIRPA*-knockout iPSC-derived macrophages

To generate a *SIRPA*-KO iPSC line, IISH2i-BM9 iPSCs derived from human bone marrow were edited using CRISPR-Cas9 to excise exon 3 of the *SIRPA* gene containing multiple CD47-binding motifs (Figures 1A–1C; S1A, S1B, and S1C).[29–32] Previously, a serum-free, xeno-free, component-defined 2D method was developed by our group for *in vitro* hematopoietic differentiation that utilizes morphogen-driven formation of the hemogenic endothelium by day 5 followed by generation of multipotent hematopoietic progenitors (HPs) with lymphoid and myeloid potential on day 8–9 (Figure 1D).[33] Throughout the differentiation, *SIRPA*-KO cells displayed similar morphology to wild-type (WT) iPSCs, exhibiting the endothelial-to-hematopoietic transition beginning on day 5 and continuing through days 6–9 during *in vitro* hematopoiesis to produce multipotent HPs, with over 90% of the cell culture expressing CD43 (Figures 1E and 1F). However, the number of HPs derived per one iPSC was found to be significantly higher in *SIRPA*-KO cell cultures compared to WT, suggesting a possible alternative role of SIRPα signaling during blood formation (Figure 1G). Nonetheless, when the multipotency of HPs was assessed in a colony-forming unit (CFU) assay, *SIRPA*-KO cell cultures generated similar numbers of granulocyte (G), erythroid (E), macrophage (M), and granulocyte-macrophage (GM) colonies as WT, indicating the ablation of SIRPα does not skew hematopoietic lineage commitment of progenitors (Figure S1D).

To generate macrophages, we continued differentiation of HPs with IF9S media supplemented with myeloid-supportive cytokines for 6 days (Figure 1H). Cells from *SIRPA*-KO and WT cultures displayed a similar phenotype with marked expression of CD45, CD14, CD11b, CD16, and CD18, signifying ablating SIRPα does not affect the development of the myeloid-cell lineage (Figure S1E). Moreover, after treatment with additional M-CSF for 4 days to drive macrophage generation (Figure 1H), both *SIRPA*-KO and WT cell lines possessed near-pure populations of iMacs with an appropriate morphology displaying large diameter and vacuolated cytoplasm, with *SIRPA*-KO iMacs possessing slightly smaller diameters (Figure 1I; Figure S1F). Ablation of SIRPα protein expression was validated by immunoblotting terminally differentiated WT and *SIRPA*-KO iMacs using an anti-human SIRPα antibody targeting the extracellular CD47-binding region of the SIRPα receptor (Figure 1J). To evaluate the plasticity of *SIRPA*-KO iMacs, day 19 iMacs were treated with M1-promoting LPS + IFN-γ or M2-promoting IL-4 for 48 h

(F) Expression of CD43 in WT and *SIRPA*-KO day 9 floating HPs was analyzed by flow cytometry.

(G) The yield of HPs from WT and *SIRPA*-KO cultures on day 9 of differentiation. Results are shown as mean ± SEM (WT $n = 27$, KO $n = 17$). ****$p < 0.0001$, Welch's t test.

(H) Schematic for generation of macrophages from floating HPs.

(I) Morphology of WT and *SIRPA*-KO iPSC-derived macrophages (iMacs). Cells were stained with Wright-Giemsa and imaged using brightfield microscopy.

(J) Western blot of WT and *SIRPA*-KO iMacs shows lack of SIRPα protein production in KO cells. Anti-human SIRPα antibody recognizes extracellular CD47 binding region (55 kDa). Glyceraldehyde-3-phosphate dehydrogenase (GAPDH) was used as a loading control (37 kDa).

(K) Expression of CD14, CD163, CD206, CD86, and CD80 in WT and *SIRPA*-KO iMacs stimulated for 48 h with IFN-γ + LPS (M1) or IL-4 (M2) or unstimulated (M0) and unstained (Uns.) macrophages were used as a control.

Analysis is representative of $n = 3$.

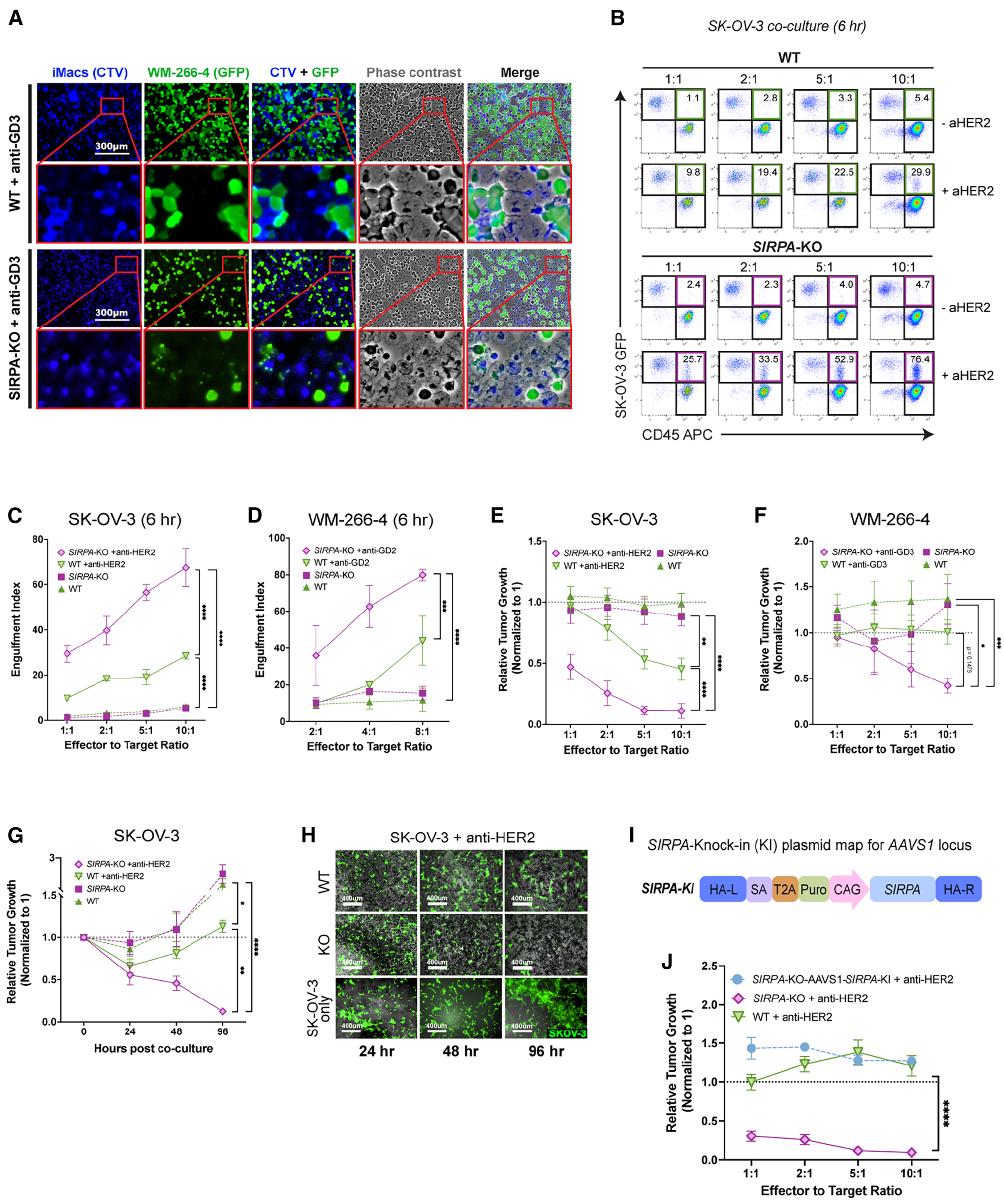

Figure 2. *SIRPA*-KO iMacs possess superior antibody-dependent antitumor capacity *in vitro*

(A) Visualization of phagocytosis by WT or *SIRPA*-KO iMacs by fluorescent and phase contrast microscopy. iMacs were stained with Cell Trace Violet (CTV), then co-cultured with WM-266-4 GFP+Luc2+ cancer cells +/− anti-GD3 at a 1:1 effector-to-target ratio. After 5 h, cytospins were made. The first row is 10× magnification, and the second row is zoomed in from the selected ROI in the red box depicted in the first row.

(B–D) Quantification of iMac ADCP using flow cytometry. (B–C) iMacs were cultured with SK-OV-3 GFP-Luc2+ cancer cells +/− anti-HER2 for 6 h or (D) with WM-266-4 GFP-Luc2+ cancer cells +/− anti-GD2 for 6 h at indicated E:T ratios. After 6 h, cells were analyzed by flow cytometry. (C–D) Engulfment index was calculated as (# double-positive GFP+CD45+ cells) / (total # of GFP+ cells) × 100. Results are mean ± SEM (n = 3); $^{**}p$ = 0.0011, $^{***}p$ = 0.0005, $^{****}p < 0.0001$, two-way ANOVA.

(E–F) Luciferase-based assay was used to quantify tumor cell growth. WT or *SIRPA*-KO iMacs were co-cultured for 48 h with (E) SK-OV-3 GFP-Luc2+ cancer cells +/− anti-HER2 or (F) WM-266-4 GFP-Luc2+ cancer cells +/− anti-GD3 at indicated E:T ratios. (E-F) Results are shown as mean ± SEM (n = 3), $^{*}p$ = 0.0488, $^{**}p$ = 0.0014, $^{***}p$ = 0.0005, $^{****}p < 0.0001$, two-way ANOVA.

(legend continued on next page)

and analyzed via flow cytometry. Unpolarized (M0), classically polarized (M1), and alternatively polarized (M2) *SIRPA*-KO iMacs highly resembled the differential expression of common macrophage markers in comparison to WT iMacs without any statistical differences (Figure 1K; Figure S1G). Overall, the differential expression of macrophage polarization markers in *SIRPA*-KO iMacs demonstrates *SIRPA*-KO iMacs' phenotypic plasticity in response to fluctuations of pro- and anti-inflammatory signaling.

SIRPA-KO iMacs possess superior antibody-dependent phagocytosis and cytotoxicity of solid tumor cancer cells *in vitro*

Given the established role of SIRPα in CD47-mediated inhibition of phagocytosis triggered by pro-phagocytic stimuli,[3,4,7] we tested the impact of *SIRPA*-KO on antibody-dependent cellular phagocytosis (ADCP) of CD47-expressing solid tumors (Figure S2A), including SK-OV-3 ovarian cancer and WM-266-4 melanoma expressing GFP-Luc2. Co-culture of *SIRPA*-KO iMacs with tumors in the presence of mAb triggered ADCP as determined by observing engulfed particles of GFP+ WM-266-4 within *SIRPA*-KO iMacs by immunofluorescent imaging after a 5-h co-culture (Figure 2A). Moreover, the percentages of WM-266-4 and SK-OV-3 phagocytosed by *SIRPA*-KO iMacs in the presence of anti-GD2 or anti-HER2 mAb, calculated by a defined flow cytometry gating strategy to gate out doublets, were significantly higher as compared with WT iMacs (Figure S2B; Figures 2B–2D). However, co-culturing *SIRPA*-KO iMacs with WM-266-4 or SK-OV-3 cells without any mAb did not trigger interactions between iMacs and tumors or produce any antitumor response, indicating that the lack of SIRPα does not trigger phagocytosis of CD47-expressing cancer cells without a prophagocytic signal (Figures 2B–2D; Figure S2C).

To assess overall tumor cell growth control, *SIRPA*-KO or WT iMacs and luciferase-expressing cancer cell lines were co-cultured for 48 h at varying effector-to-target ratios with or without mAb. *SIRPA*-KO iMacs demonstrated significantly heightened tumor cell growth control against both SK-OV-3 and WM-266-4 cancer cells compared to WT iMacs (Figures 2E and 2F). When challenged with SK-OV-3, *SIRPA*-KO iMacs reached a saturation point at a 5:1 effector-to-target ratio, killing up to 90% of target cells, whereas efficacy against WM-266-4 was more limited, reaching around 50% cytotoxicity (Figures 2E and 2F). Since the TME can affect macrophage polarization and inhibit antitumor activities, we sought to assess *SIRPA*-KO iMacs' antitumor responses in the presence of anti-HER2 mAb over longer durations of time (24, 48, and 96 h). Thus, we found that *SIRPA*-KO iMacs stimulated with a single dose of anti-HER2 continually eliminated SK-OV-3 tumors over the entirety of the 96 h, whereas WT iMacs reached peak cytotoxicity at around 30% and were unable to control the outgrowth of SK-OV-3 by 48 h (Figures 2G and 2H). To ensure that the superior antitumor activity of iMacs was truly related to *SIRPA* deletion rather than a potential off-target effect, we re-established SIRPα expression in the *SIRPA*-KO iPSC line by inserting the *SIRPA* gene into the *AAVS1* locus (Figure 2I; Figure S2D–F). Indeed, iMacs generated from *SIRPA*-KO-*AAVS1*-*SIRPA*-knockin (KI) iPSCs rescued the phenotype of WT iMacs and were unable to control SK-OV-3 tumor growth (Figure 2J). These results indicate that SIRPα ablation in macrophages significantly enhances their antibody (Ab)-mediated antitumor functions against CD47-expressing solid tumor cell lines, including phagocytosis and sustained inhibition of tumor growth.

SIRPA-KO iMacs possess transcriptional changes after prolonged tumor exposure

To explore transcriptomic differences between WT and *SIRPA*-KO iMacs before and after tumor exposure, we performed bulk RNA sequencing of WT and *SIRPA*-KO iMacs either cultured alone, with SK-OV-3 and anti-HER2 for 24 h, or with SK-OV-3 and anti-HER2 for 96 h (Figure 3A). In this manner, we sought to analyze the distinction between iMacs' transcriptomic phenotype at 24 and 96 h post-tumor exposure. iMacs isolated from cultures showed more than 97% purity, with almost all residual GFP^+ cells residing within the $CD45^+$ population (Figure S3B), confirming the absence of contaminating SK-OV-3 cells. Upon global transcriptomic analysis, we observed that WT and *SIRPA*-KO iMacs clustered closer together at baseline (iMacs alone) and after 96 h than after early tumor exposure at 24 h (Figure 3B; Figure S3A). The increased variance at 24 h likely reflects the presence of incompletely digested SK-OV-3 transcripts persisting during early tumor exposure, a phenomenon commonly observed in the field.[34]

At baseline prior to tumor exposure, we found only a small number of genes were differentially expressed by *SIRPA*-KO iMacs, including the significant downregulation of MHC class II-associated genes *HLA-DRA*, *HLA-DPB1*, *CD74*, and *CIITA*, and genes associated with M2-TAM phenotype *MMP9*,[35–37] *IL4I1*,[38,39] and *POTEE*,[40] by *SIRPA*-KO iMacs (Figure 3C). Due to the high number of transcripts associated with SK-OV-3 tumor at "early tumor exposure," we found no significant difference between WT and *SIRPA*-KO iMacs' transcriptome in that same 24-h condition (Figure 3C). However, we observed downregulation of *HLA-DRA*, *IL4I1*, and *POTEE* expression in *SIRPA*-KO vs. WT iMacs 96 h after exposure to SK-OV-3 and anti-HER2 (Figure 3C).

Upon further Gene Set Variation Analysis (GSVA), we verified minimal differences in transcriptomic patterns between WT and *SIRPA*-KO iMacs at baseline and 24 h after tumor exposure (Figure 3D). However, 96 h post-exposure, the transcriptomic patterns between

(G–H) Time kinetics of tumor growth at a 10:1 E:T ratio. (G) Results are mean ± SEM (n = 6); *p = 0.0153, **p = 0.0013, ****p < 0.001, two-way ANOVA. (H) Images depicting GFP+ SK-OV-3 cell viability in cultures with iMacs and mAb were taken via fluorescence microscopy at 24, 48 and 96 h.

(I) Schematic representation of the *SIRPA* knockin (KI) molecule construct for the *AAVS1* safe harbor locus.

(J) Tumor cell growth assay for 48 h at indicated E:T ratios demonstrates restoration of the checkpoint response following expression of SIRPα in *SIRPA*-KO cells. Results are shown as mean ± SEM (n = 3); ****p < 0.0001, two-way ANOVA.

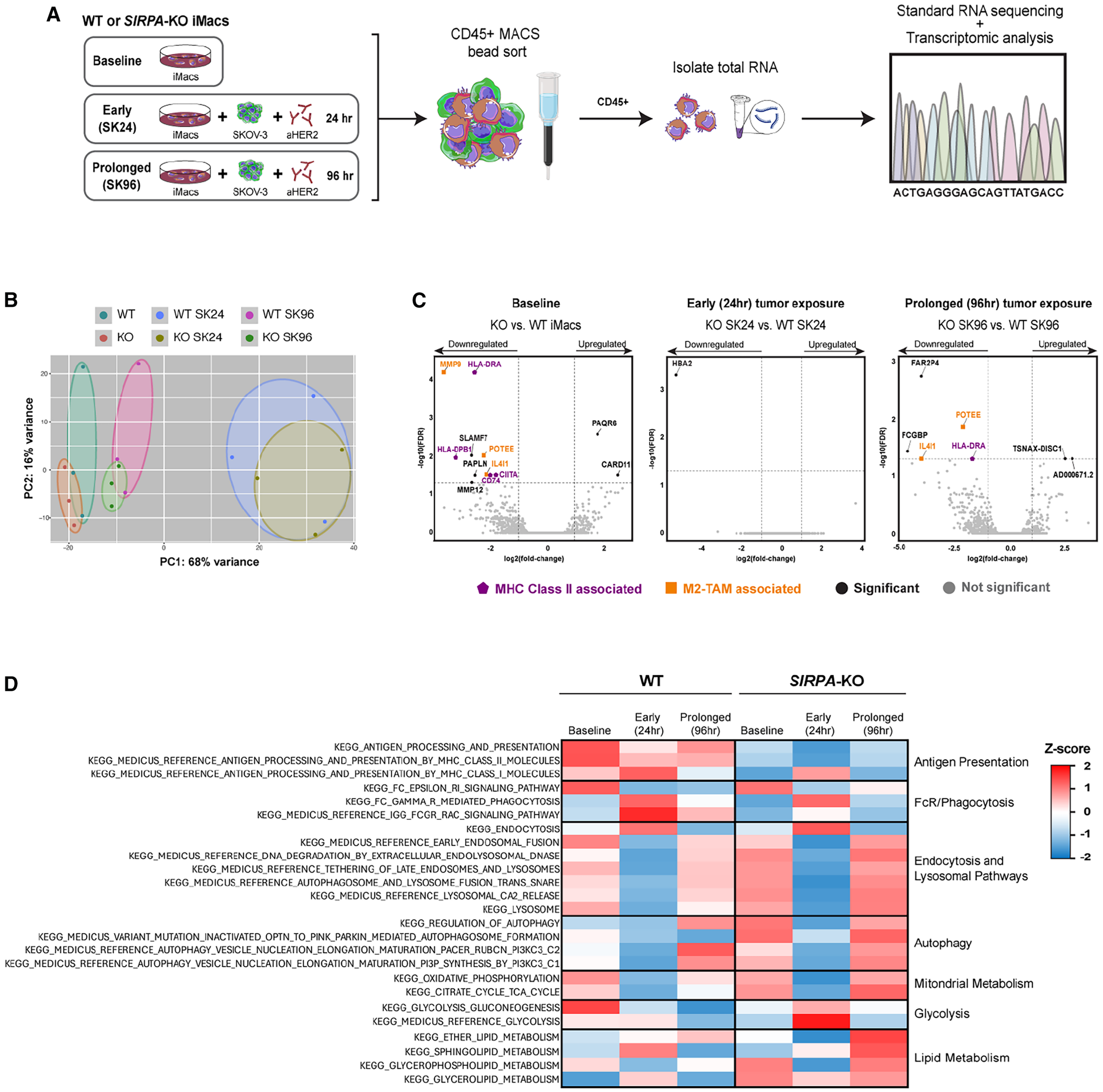

Figure 3. Ablating SIRPα alters transcriptome of iMacs after prolonged tumor and mAb exposure

(A) Schematic of preparation of WT and *SIRPA*-KO iMacs for bulk RNA sequencing. WT and *SIRPA*-KO iMacs were either cultured alone for 24 h (Group 1), with SK-OV-3 and anti-HER2 for 24 h (SK24; Group 2), or with SK-OV-3 and anti-HER2 for 96 h (SK96; Group 3). In short, cell cultures of all groups were collected at specified time points, iMacs were isolated by CD45^{+} MACS bead sorting, and total RNA was isolated from the subsequent CD45+ cell cultures of all groups. Bulk RNA sequencing was then performed on total RNA isolates after rRNA depletion. All data represented are n = 3 independent iMac differentiations and tumor co-cultures per group.

(B) Wild-type (WT) and *SIRPA*-KO (KO) iMacs were cultured alone in media or co-cultured with SK-OV-3 ovarian cancer (SK) for 24 h or 96 h (SK24; SK96) in the presence of anti-HER2 mAb. Plot shows PC1 vs. PC2 variance plots of WT, KO, WT SK24, KO SK24, WT 96, and SK96 global transcriptomic differences.

(C) Volcano plots depicting differentially expressed genes of "Baseline": *SIRPA*-KO (KO) iMacs vs. WT alone, "Early" (24 h) tumor exposure: *SIRPA*-KO iMacs vs. WT cultured with SK-OV-3 and anti-HER2 for 24 h (KO SK24 vs. WT SK24) and "Prolonged (96 h) tumor exposure": *SIRPA*-KO iMacs vs. WT cultured with SK-OV-3 and anti-HER2 for 96 h (KO SK96 vs. WT SK96). Plots depict differentially expressed gene (DEG) expression from *SIRPA*-KO cell cultures in comparison to WT. Genes highlighted in purple are significant and associated with MHC class II antigen presentation, genes highlighted in orange are significant and associated with pro-tumoral macrophages, genes in black are significant, and genes in gray are not significant.

(legend continued on next page)

WT and *SIRPA*-KO iMacs considerably diverged in many gene pathways involving macrophage-related effector functions and metabolism (Figure 3D). GSVA revealed substantial downregulation of pathway activity in genes associated with antigen processing and presentation, including MHC class I- and II-related genes, in *SIRPA*-KO iMacs across all treatments, consistent with our previous findings (Figure 3C). We also found that 96 h after tumor exposure, *SIRPA*-KO iMacs are highly enriched in gene sets associated with endocytosis, lysosomal pathways, and autophagy (Figure 3D), consistent with enhanced ADCP activity in *SIRPA*-KO iMacs. Finally, SIRPA-KO iMacs show substantial differences in metabolic activity after prolonged tumor exposure, including enrichment in mitochondrial- and lipid-related metabolism and a spike in activity of glycolysis- and glucogenesis-related gene set pathways at 24 h post-tumor exposure compared to WT (Figure 3D). Overall, these findings underscore that *SIRPA*-KO iMacs are enriched for gene pathway activity related to downstream processing of phagocytic content and metabolic activity after prolonged tumor exposure.

SIRPα ablation in iMacs reverses mAb-mediated hypophagia-related exhaustion

Macrophages exposed to mAb-opsonized target cells display an initial, rapid burst of ADCP, lasting less than an hour, followed by a pronounced decline in phagocytic activity persisting for days, even with subsequent exposure to mAb-opsonized targets as a result of downregulated FcR.[41] This markedly diminished capacity of ADCP, referred to as hypophagia, impairs macrophage-mediated clearance of mAb-opsonized target cells and can be considered a form of macrophage exhaustion that hinders antitumor function. To evaluate the effect of SIRPα ablation on iMac exhaustion, we assessed the cytotoxicity of iMacs following serial tumor exposures of SK-OV-3-GFP-Luc2+ cells in the presence of anti-HER2 mAb (Figure 4A). We found that *SIRPA*-KO iMacs retained a significantly heightened capacity for tumor cell growth control against SK-OV-3 throughout the five tumor exposures, unlike WT iMacs, which only retained effective control up to 72 h (Figures 4B and 4C). Addition of anti-HER2 alone to SK-OV-3 did not control tumor growth, indicating tumor killing was due to the presence of *SIRPA*-KO iMacs and anti-HER2 together (Figures 4B and 4C). To validate that the ablation of SIRPα is truly preventing hypophagia, the chronic and pronounced decline of ADCP in macrophages, WT and *SIRPA*-KO iMacs were isolated after 96 h of serial exposure to an unmodified tumor lacking GFP and re-challenged with fresh SK-OV-3-GFP-Luc2+ and anti-HER2 for a 2-h phagocytosis assay (Figure 4D). As expected, we found that *SIRPA*-KO iMacs had a considerably higher capacity for ADCP, engulfing 3-fold more GFP$^+$ tumor targets than WT iMacs (Figure 4E).

Previous studies with lymphoma cells have shown that loss and proteolytic degradation of activating FcRs are primary causes of hypophagia.[41] Indeed, when we compared expression of FcRI (CD64), FcRII (CD32), and FcRIII (CD16) at 96 h post-serial tumor exposure in WT iMacs, we observed their downregulation, with CD32 showing the sharpest decrease. In contrast, *SIRPA*-KO iMacs showed greater retention of all FcRs, with CD16 even displaying upregulation (Figure 4F). In addition, DQ-OVA assay demonstrated the impaired overall antigen-processing ability of WT compared to *SIRPA*-KO iMacs after serial tumor exposure (Figure 4G; Figure S4A). No differences in iMac viability were observed between the groups (Figure S4B).

To determine whether resistance to exhaustion in *SIRPA*-KO iMacs could be attributed to cytokines, we performed secretome analysis of iMacs serially exposed to SK-OV-3 tumor and anti-HER2 four times for 96 h. These studies revealed that cytokines associated with proinflammatory immunity, such as IL-6, IP-10, IL-18, IFN-γ, and MIP-1α, were all significantly diminished in the secretome of *SIRPA*-KO iMacs compared to WT iMacs (Figure S4C). These findings suggest that cytokines are unlikely to account for the better tumor control observed in *SIRPA*-KO iMacs and that exhausted WT iMacs activate pro-inflammatory pathways to overcome diminished antitumor efficacy during chronic tumor and mAb exposure.

Together, these data indicate *SIRPA*-KO iMacs not only possess superior antitumor activity due to the interruption in the “don’t eat me” CD47-SIRPα pathway, but that SIRPα serves as a key regulator of hypophagia and macrophage exhaustion in the context of chronic tumor and mAb exposure. Furthermore, ablation of *SIRPA* in iMacs reverses hypophagia-related exhaustion and sustains FcR expression, thereby amplifying their tumoricidal potential.

SIRPA-KO iMacs administered with HER2 antibody improves survival of mice with SK-OV-3 xenograft

To test the efficacy of *SIRPA*-KO iMacs *in vivo*, we engrafted NSG female mice with human SK-OV-3 ovarian cancer cells using an intraperitoneal (IP) injection to generate a disseminated metastasis model (Figure 5A). After administration of a single dose of iMacs and anti-HER2 mAb via IP injections 5 days after engraftment, we observed no significant differences in SK-OV-3 tumor burden reduction among the anti-HER2 alone, WT iMacs and anti-HER2, and *SIRPA*-KO and anti-HER2 treatment groups (Figures 5B and 5C; Figure S5A). However, treatment with just a single injection of *SIRPA*-KO iMacs and anti-HER2 significantly prolonged survival more than the other treatment groups, indicating a potential difference in the anti-solid tumor activities of *SIRPA*-KO iMacs in comparison to WT iMacs (Figure 5D).

(D) Gene Set Variation Analysis (GSVA) heatmap of selected macrophage-related functions. Heatmap from red to blue shows z score-normalized GSVA enrichment scores for selected pathways related to antigen presentation, Fc receptor (FcR) and phagocytosis, endocytosis and lysosomal pathways, autophagy, mitochondrial metabolism, glycolysis, and lipid metabolism across WT and SIRPA-KO iMacs under baseline conditions, and after “early” (24 h) or “prolonged” (96 h) SK-OV-3 tumor and anti-HER2 exposure. *Z* score normalization was performed across conditions for each gene set.

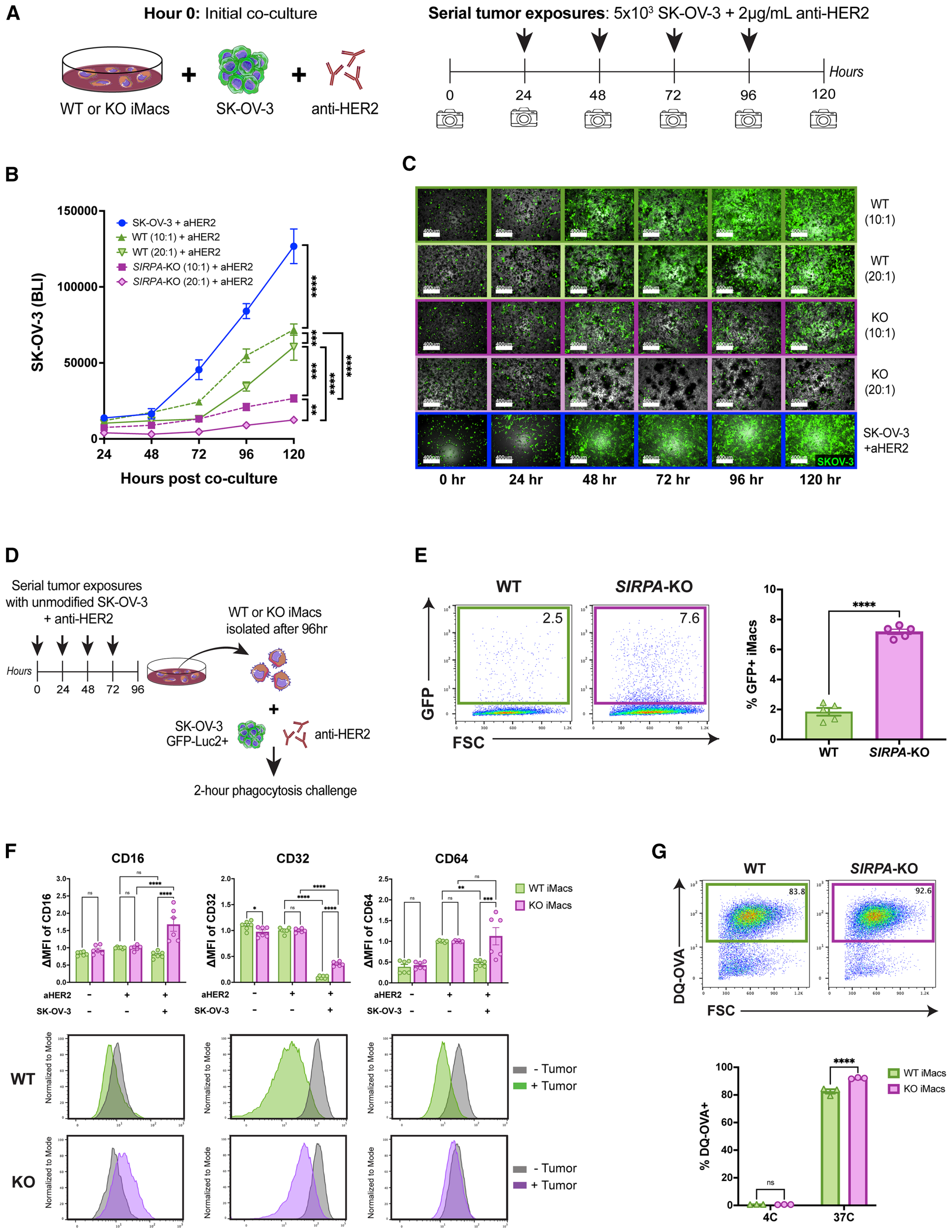

Figure 4. *SIRPA*-KO iMacs resist mAb-driven macrophage exhaustion during serial tumor exposures

(A) Schematic for *in vitro* serial tumor exposure assay. WT and *SIRPA*-KO iMacs were co-cultured with SK-OV-3 GFP-Luc2+ cancer cells + anti-HER2 at an initial 10:1 or 20:1 E:T ratio. Every 24 h, total media were replenished with fresh SK-OV-3 +/− anti-HER2 without disturbing the existing co-culture.

(B) Luciferase assay was used to detect SK-OV-3 tumor cell growth by bioluminescence (BLI) during *in vitro* serial tumor exposure assay. Results are mean ± SEM ($n = 6$); $^{**}p = 0.0034$, $^{***}p < 0.001$, $^{****}p < 0.0001$, two-way ANOVA.

(C) Fluorescence microscopy images of GFP+ SK-OV-3 viable cells during *in vitro* serial tumor exposure assay.

(legend continued on next page)

To assess whether an antitumor effect can be improved by injecting iMacs intravenously (IV), we treated mice 5 days after SK-OV-3 engraftment with either anti-HER2 alone, WT iMacs and anti-HER2, or *SIRPA*-KO iMacs and anti-HER2, followed by a second dose of anti-HER2 two weeks after the initial treatment (Figure 5E). In contrast to the previous experiment, *SIRPA*-KO iMacs administered IV with anti-HER2 immediately reduced tumor burden within one week compared to other treatment groups and continued to suppress tumor growth through 50 days (Figures 5F and 5G; Figure S5B). The IV injection of *SIRPA*-KO iMacs and anti-HER2 resulted in the longest median survival time for SK-OV-3-engrafted NSG mice compared to other groups, supporting our previous results in *SIRPA*-KO iMacs' capacity in reducing tumor (Figure 5H). Taken together, we have demonstrated that combination therapy using *SIRPA*-KO iMacs and anti-HER2 improves survival of SK-OV-3-engrafted mice, regardless of administration method. However, Ab-directed antitumor effects of *SIRPA*-KO iMacs were modest, suggesting that solely ablating SIRPα in macrophages is not sufficient to drive iMac-mediated clearance of ovarian tumors in a host lacking lymphoid cells.

Ablating SIRPα in CAR-iMacs overcomes exhaustion and markedly enhances their antitumor activities *in vitro*

In prior studies, CAR expression in iMacs markedly upregulated *SIRPA* expression after exposure to tumor.[28] Thus, we explored whether ablation of SIRPα would bolster the tumor-killing potential by anti-disialoganglioside (GD2) CAR-expressing iMacs against CD47$^+$ solid tumors. The GD2 antigen is highly expressed in a variety of pediatric and adult tumors, including melanoma, neuroblastoma, high-grade glioma, osteosarcoma, triple-negative breast cancer, and non-small cell lung carcinoma, while its expression in normal post-natal tissues is low and limited to peripheral nerve pain fibers, making it an ideal tumor-antigen target.[42] Following knockin of the PBMC-3-1 iPSC line with a third-generation anti-GD2-CAR inserted into the *AAVS1* locus (Figure 6A) and knockout of *SIRPA* (GD2-*SIRPA*-KO-CAR-iPSCs; Figure S6A–D), we evaluated antitumor cytotoxicity of GD2-*SIRPA*-KO-CAR-iMacs against GD2-expressing CHLA-136 GFP-Luc2+ neuroblastoma and GD2-negative SK-OV-3 ovarian carcinoma. We found GD2-*SIRPA*-KO-CAR-iMacs possess significantly heightened antitumor cytotoxicity against CHLA-136 neuroblastoma than GD2-CAR-iMacs, while both fail to kill GD2-negative SK-OV-3 tumor across all ratios, indicating specificity to GD2-expressing target cells (Figures 6B and 6C). To validate the ablation of SIRPα produces a robust antitumor effect across CAR iMacs and not just a function of iPSC variability, we generated GD2-CAR and GD2-*SIRPA*-KO-CAR iMacs from a second human PSC line, IISH2i-BM9 iPSCs, and confirmed that knocking out *SIRPA* in GD2-CAR iMacs significantly enhances their antitumor cytotoxicity against CHLA-136, regardless of parent iPSC line (Figure S6E).

Due to the TME's ability to inhibit antitumor functions of macrophages, we sought to evaluate the capacity of GD2-*SIRPA*-KO-CAR-iMacs to sustain antitumor activities over longer durations of time during a challenge with GD2-expressing cancer cell lines (Figure S2A): CHLA-136 neuroblastoma, and WM-266-4 melanoma, for 96 h. We found GD2-*SIRPA*-KO-CAR-iMacs possess a significantly heightened burst of initial cytotoxicity against CHLA-136 compared to CAR-iMacs, then continually inhibit tumor growth for 96 h until nearly 99% of the tumor cells are eliminated (Figure 6D). In contrast, GD2-CAR-iMacs with intact SIRPα were unable to reduce CHLA-136 viability until 96 h (Figure 6D). Additionally, GD2-*SIRPA*-KO-CAR-iMacs significantly reduced WM-266-4 melanoma by 96 h compared to GD2-CAR-iMacs (Figure 6E), demonstrating the ablation of SIRPα improves antitumor efficacy of CAR-iMacs against multiple GD2-expressing solid tumor cell lines. Evaluation of the secretome of iMacs revealed that GD2-*SIRPA*-KO-CAR-iMacs secrete significantly lower levels of IL-1β, IL-18, IFN-γ, MIP-1α, and TNF-α than GD2-CAR-iMacs without any stimuli (Figure 6F). However, after exposure to tumor for only 24 h, GD2-*SIRPA*-KO-CAR-iMac cultures possessed significantly higher levels of pro-inflammatory cytokines IL-1β, IL-6, and TNF-α in comparison to GD2-CAR-iMacs (Figure 6F), indicating that the strong initial burst of tumor killing observed from GD2-*SIRPA*-KO-CAR-iMacs in Figure 6B may be correlated with the activation of pro-inflammatory signaling pathways in an antigen-dependent inducible fashion. Additionally, while GD2-CAR-iMacs express some moderate levels of pro-inflammatory cytokines without stimuli, there was no significant increase in cytokine secretion levels after their exposure to tumor, which may be an indication of their poor tumor-killing capacity (Figure 6F).

Given that SIRPα ablation protects macrophages from mAb-driven hypophagia-related exhaustion (Figure 4), we wanted to investigate whether serially exposing GD2-CAR-iMacs to GD2-expressing tumors induces CAR-related exhaustion and whether SIRPα ablation affects this process. To do so, GD2-CAR or GD2-*SIRPA*-KO-CAR-iMacs were initially co-cultured at a 10:1 effector-to-target ratio with CD47-expressing CHLA-136 neuroblastoma for 24 h and were re-exposed five times to additional CHLA-136 cells every

(D) Schematic for 2-h phagocytosis challenge post 96-h serial tumor exposure assay.

(E) WT and *SIRPA*-KO iMacs from 96-h serial tumor exposure cultures were subjected to a 2-h phagocytosis assay with fresh GFP-expressing SK-OV-3 and anti-HER2 mAb, then assessed for the percentage (%) of GFP-expressing iMacs within the CD45$^+$-gated cells; results are mean ± SEM ($n = 5$); ****$p < 0.0001$, unpaired t test.

(F) WT and *SIRPA*-KO iMacs were subject to an *in vitro* serial unmodified tumor exposure assay as described in (A) for 96 h, with or without anti-HER2 or SK-OV-3 target cells, then isolated for flow cytometric analysis of CD16, CD32, and CD64 expression in CD45$^+$-gated cells. All geometric MFI values of WT and *SIRPA*-KO iMacs were normalized to their respective WT or *SIRPA*-KO iMacs with Ab only at 96 h. Results are mean ± SEM ($n = 6$); **$p = 0.0029$, ***$p = 0.0002$, ****$p < 0.0001$, two-way ANOVA.

(G) Flow cytometric analysis of DQ-OVA uptake and digestion by iMacs from 96-h serial tumor exposure cultures. Results are mean ± SEM ($n = 3$), ****$p < 0.0001$, multiple unpaired t tests.

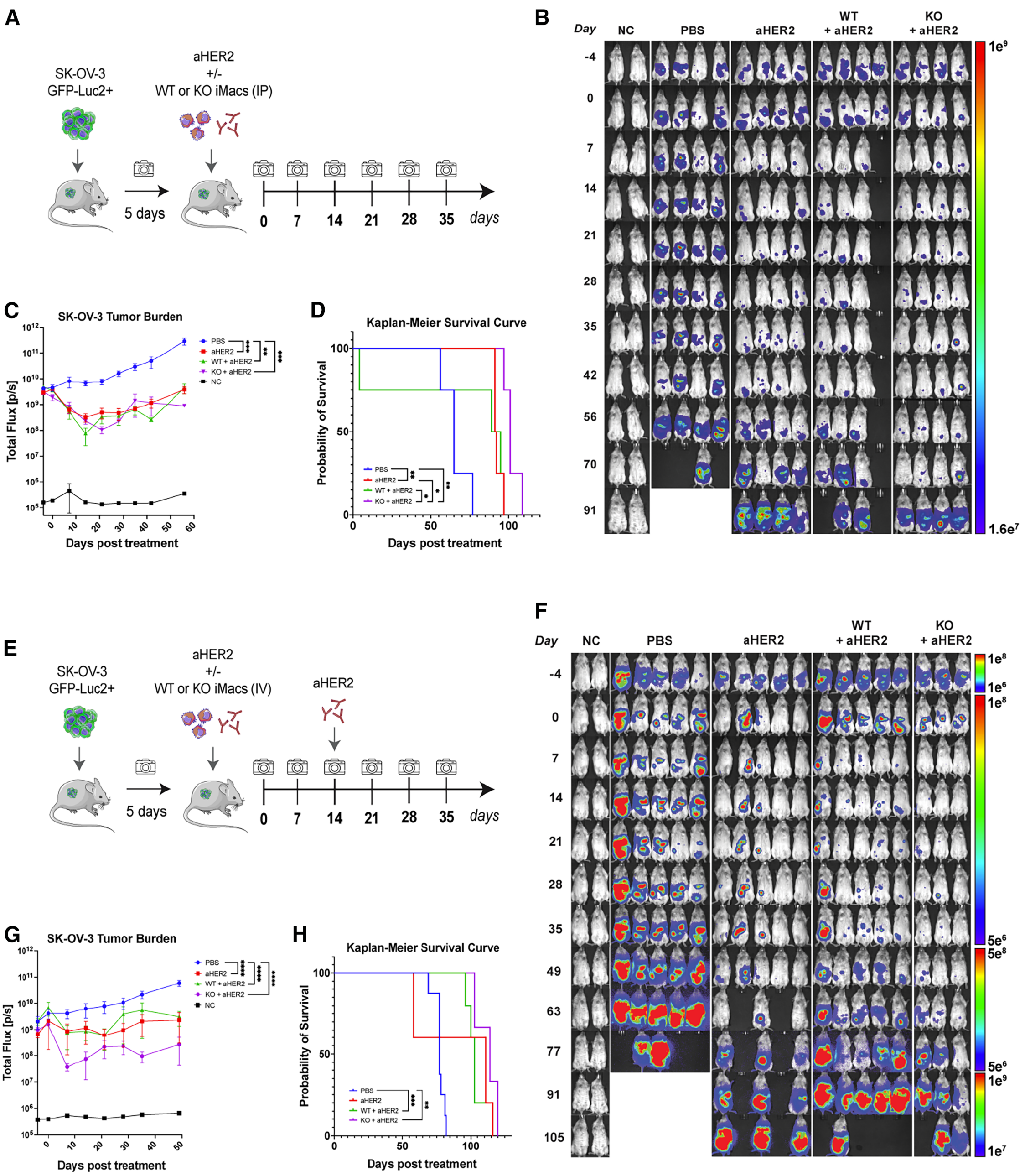

Figure 5. *SIRPA*-KO iMacs + anti-HER2 show little efficacy against an *in vivo* SK-OV-3 tumor model of peritoneal metastases

(A) Schematic of *in vivo* SK-OV-3 tumor model establishment. Female NSG mice were engrafted with 7.5×10^5 SK-OV-3 GFP-Luc2+ cancer cells via IP injection and 5 days later, treated with either 100 μg anti-HER2 alone or with 7×10^6 WT or *SIRPA*-KO iMacs via IP injection.

(B) Bioluminescent images of tumor xenografts over time for each treatment group. NC, negative control.

(C) Quantification of SK-OV-3 tumor xenografts over time for each treatment group. NC, negative control. Results are mean total flux (photons/s) ± SEM (NC $n = 2$, PBS $n = 4$, aHER2 $n = 4$, WT + aHER2 $n = 4$, KO + aHER2 $n = 4$); $^{**}p = 0.002$, $^{***}p < 0.001$, two-way ANOVA.

(D) Kaplan-Meier survival analysis of mice for each treatment group from (C) as analyzed using the log rank test: $^{*}p = 0.0169$, $^{**}p = 0.0069$.

(E) Schematic of *in vivo* SK-OV-3 tumor model establishment. Female NSG mice were engrafted with 7.5×10^5 SK-OV-3 GFP-Luc2+ cancer cells via IP injection and 5 days later, treated with either 50 μg aHER2 alone via IP injection or with 5×10^6 WT or *SIRPA*-KO iMacs via IV injection. On day 14, an additional 50 μg aHER2 was given via IP injection.

(legend continued on next page)

24 h (Figure 7A). We found that GD2-CAR-iMacs failed to clear tumor cells within 24 h and promoted tumor growth throughout the remainder of the tumor exposures (Figures 7B and 7C), consistent with our previous findings that GD2-CAR-iMacs possessed significantly delayed cytotoxicity during a single tumor exposure (Figure 6D). Conversely, we found GD2-*SIRPA*-KO-CAR-iMacs immediately killed approximately 80% of the CHLA-136 tumor upon initial exposure at 24 h and continually sequestered tumor growth for the entire five tumor exposures (Figures 7B and 7C). Interestingly, the significant reduction in CHLA-136 tumor growth in GD2-*SIRPA*-KO-CAR-iMac co-cultures was also accompanied by the formation of macrophage clusters engulfing GFP-positive tumor cells (Figure S6F). This phenomenon was first observed after the second tumor exposure at 48 h and persisted after every subsequent tumor exposure.

Previously, we found that *SIRPA*-KO iMacs are resistant to mAb-induced hypophagia and possess heightened phagocytic capacity after serial tumor exposure in comparison to WT iMacs. To elucidate if CAR-driven hypophagia is affected by SIRPα in the same manner, CAR iMacs that were serially exposed to unmodified CHLA-136 cells for 96 h were re-challenged with fresh GFP-Luc2+ CHLA-136 tumor targets for 2 h and analyzed for phagocytosis (Figure 7D). Indeed, we confirmed significantly heightened capacity for phagocytosis of GFP-Luc2+ CHLA-136 by GD2-*SIRPA*-KO-CAR iMacs compared to SIRP-intact GD2-CAR iMacs (Figure 7E), demonstrating SIRPα's role in modulating hypophagia in multiple contexts.

Given the impact of *SIRPA* on surface Fc receptor expression, we sought to determine whether the improved tumor-killing in SIRPα-ablated GD2-CAR iMacs is due to better retention of surface GD2-CAR. As predicted, after 96 h of serial tumor exposures, GD2-*SIRPA*-KO-CAR iMacs possessed increased surface CAR expression compared to GD2-CAR iMacs (Figure 7F) and exhibited better viability (Figure S6G), despite slight reduction in the uptake and proteolytic degradation of DQ-OVA and (Figure 7G). These results suggest that SIRPα ablation reverses CAR-driven hypophagia through the improved maintenance of surface CAR expression on iMacs and enhances the tumor-killing ability of CAR-iMacs even after multiple rounds of tumor exposure.

SIRPα ablation in GD2-CAR-iMacs reduces metastatic neuroblastoma burden *in vivo*

To evaluate the efficacy of GD2-*SIRPA*-KO-CAR-iMacs *in vivo*, we engrafted male and female NCG-X mice with human CHLA-136 neuroblastoma through IV injection to generate a disseminated metastasis model (Figure 8A). We found GD2-*SIRPA*-KO-CAR-iMacs significantly delayed tumor burden 1 week after initial administration and continually delayed tumor growth significantly more than GD2-CAR-iMacs by day 21 (Figures 8B and 8C; Figure S7A). However, treatment did not have a significant impact on survival (Figure S7B) nor long-term tumor control (Figures 8B and 8C; Figure S7C–E). Overall, ablating SIRPα within CAR-iMacs significantly improved their CAR-dependent antitumor capacity *in vitro* against GD2-expressing solid tumor cancers, enhanced pro-inflammatory cytokine expression, and mitigated neuroblastoma tumor burden xenograft *in vivo*, but additional treatments may be needed to prolong survival in highly aggressive, metastatic settings.

DISCUSSION

Recent advances in cellular immunotherapy have led to significant progress in treating many types of cancer, particularly CAR-T cell therapies for the treatment of hematological cancers. However, these therapies have shown limited efficacy against advanced-stage solid tumors and highlight the urgent need for novel therapeutic approaches specifically designed to target solid tumors and molecular pathways within the TME. Previous studies have shown that macrophages from $SIRPA^{-/-}$ mice exhibit enhanced phagocytic capacity against CD47-expressing target cells.[43,44] Additionally, silencing SIRPα with a short hairpin RNA or with built-in CD47 blocker bolsters the antitumor effects of somatic CAR-macrophages.[45,46] Our study adds to these approaches by discovering the anti-hypophagic effect of *SIRPA* knockout in iMacs and demonstrating the utility of using uniformly edited human *SIRPA*-KO iPSC-derived iMacs as an "off-the-shelf" platform to target solid tumors.

We revealed that iMacs derived from *SIRPA*-KO human iPSCs possess superior capacity for mAb-driven phagocytosis and cytotoxicity against various solid tumor cell lines *in vitro* and continually suppress tumor growth compared to WT iMacs. In addition, *SIRPA*-KO iMacs demonstrated their safety against $CD47^{+}$ cells without directed stimuli; circumventing the complex and off-target effects observed while using CD47-blocking antibodies. In contrast to significant upregulation of pro-inflammatory signature in somatic macrophages following introduction of CD47 blockers using adenovirus,[47] we found only minor differentially expressed genes between *SIRPA*-KO and WT iMacs. However, these included significant downregulation of three M2-TAM, pro-tumorigenic genes: *IL4I1*,[38,39] *POTEE*,[40] and *MMP9*,[35–37] in *SIRPA*-KO iMacs. IL4I1 activates the aryl hydrocarbon receptor, suppresses adaptive immunity, and promotes cancer cell motility.[39] POTEE may play a role in the regulation of macrophage survival and invasion through mTORC2 activation.[40] MMP9 is implicated in promotion of tumor growth and metastasis.[48] Thus, downregulation of pro-tumorigenic genes could provide an additional advantage for using *SIRPA*-KO iMacs for cellular immunotherapy. Additionally, further transcriptomic investigation using GSVA revealed that *SIRPA*-KO iMacs exhibit enriched activity across gene pathways associated with mitochondrial

(F) Bioluminescent images of tumor xenografts over time for each treatment group. NC, negative control.
(G) Quantification of SK-OV-3 tumor xenografts over time for each treatment group. NC, negative control. Results are mean total flux (photons/s) ± SEM (NC $n = 2$, PBS $n = 8$, aHER2 $n = 5$, WT + aHER2 $n = 5$, KO + aHER2 $n = 3$); ****$p < 0.0001$, two-way ANOVA.
(H) Kaplan-Meier survival analysis of mice for each treatment group from (G) as analyzed using the log rank test: **$p = 0.0066$, ***$p = 0.0007$.

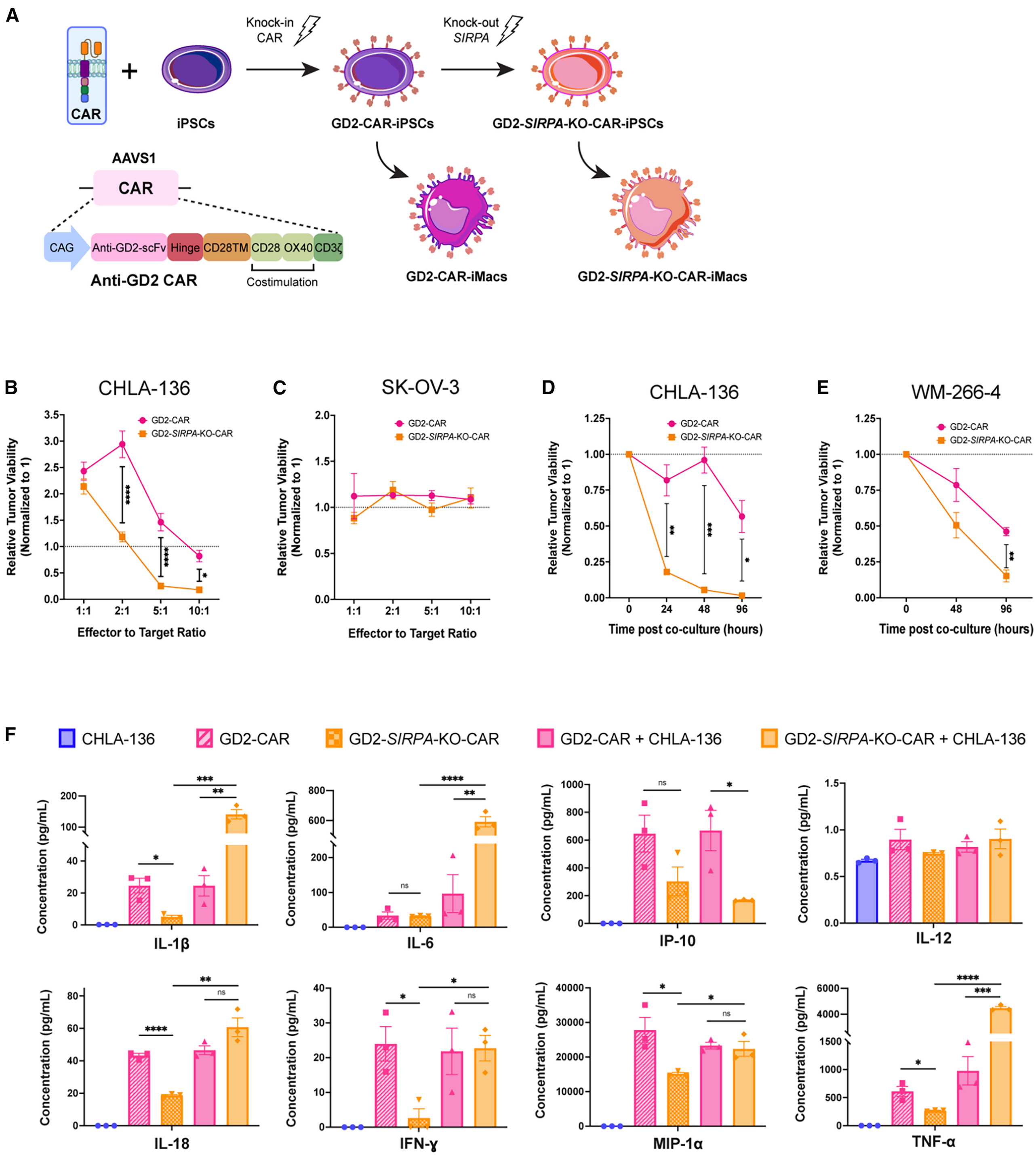

Figure 6. Ablating SIRPα potentiates the tumor-killing capacity of GD2-CAR-iMacs *in vitro*

(A) Schematic of the generation of GD2 CAR (CAR) iMacs and anti-GD2 CAR *SIRPA*-KO (GD2-*SIRPA*-KO) iMacs.

(B–C) Luciferase cytotoxicity assay was used to quantify *in vitro* tumor viability using various effector-to-target ratios. PBMC-3-1 CAR or GD2-*SIRPA*-KO iMacs were co-cultured with (B) CHLA-136 GFP-Luc2+ neuroblastoma cells or (C) SK-OV-3 GFP-Luc2+ cancer cells at indicated E:T ratios for 24 h. Data are mean ± SEM (CHLA-136 $n = 6$, SK-OV-3 $n = 3$); *$p < 0.05$, ****$p < 0.0001$, two-way ANOVA.

(legend continued on next page)

and lipid metabolism, endocytosis, lysosomal pathways, and autophagy following tumor exposure, consistent with the sustained antibody-dependent antitumoral effects and resistance to hypophagia observed *in vitro*.

Immune cell exhaustion following chronic exposure to antigens leads to progressive loss of function and inability to eliminate cancers or infectious agents. Although most immune cell exhaustion studies have primarily been focused on impaired function of T cells, studies by Pinney et al., 2020,[41] revealed exhaustion of phagocytic function of macrophages (hypophagia) following exposure to opsonized lymphoma cells. Macrophages participating in ADCP experience an initial short burst of phagocytosis followed by a rapid and long-term decline in phagocytotic capacity. This decline was associated with the loss of surface Fc receptors and a diminished expression of phosphorylated Syk that can last for several days.[41] Another study found exhaustion of Ly6C^{hi} monocytes after repeat exposures of LPS elevated NAD+, compromised mitochondrial function, and elevated cellular ROS.[49] In this study, we revealed, similarly to the prior report with lymphoma cells,[41] that WT-iMacs experience severely diminished ADCP and tumor cell growth control following serial exposures of ovarian cancer cells, which was associated with loss of surface expression of Fc receptors. In contrast, *SIRPA-KO* iMacs maintained better FcR expression. We also provided evidence of antibody-mediated exhaustion in CAR-iMac-mediated antitumor responses following serial exposures to neuroblastoma. By repeatedly challenging iMacs with CHLA-136 neuroblastoma *in vitro*, we observed that GD2-CAR-iMacs promoted tumor growth over time, while GD2-*SIRPA*-KO-CAR-iMacs consistently eliminated tumor cells and heightened GD2-CAR expression, indicating that SIRPα ablation allows for better maintenance of surface CAR expression and tumoricidal potential during chronic tumor exposure.

Multiple groups, including ours, have already demonstrated the feasibility and safety of using CAR-Ms for treating solid tumors. However, given their limited efficacy against these tumors, there is a need to target immunosuppressive molecular pathways within the TME that significantly inhibit their antitumor potential. When analyzing transcriptomic differences between anti-GD2-CAR-Ms and unmodified macrophages in a previous study by our group, we noticed a vast upregulation of *SIRPA* after exposure to GD2-expressing tumor cell line.[28] This upregulation of *SIRPA* may contribute to intensified CD47-SIRPα signaling, which subsequently may result in the diminished antitumor capacity of CAR-Ms. Therefore, in this study, we explored how the ablation of SIRPα in anti-GD2-CAR-expressing iMacs influences their antitumor capacity against GD2-expressing solid tumor malignancies, including neuroblastoma, the most common extracranial solid tumor malignancy among pediatric patients with high risk of relapse and mortality.[50,51] To this end, we found GD2-*SIRPA*-KO-CAR-iMacs possess superior cytotoxicity against GD2-expressing CHLA-136 neuroblastoma, emphasized with a rapid, initial clearing of tumor cells accompanied with the elevated secretion of pro-inflammatory cytokines IL-1β, IL-6, and TNF-α in a highly inducible manner.

Despite the marked effect of SIRPα ablation on the antitumor activity of iMacs *in vitro*, we observed only a modest effect of *SIRPA*-KO iMacs on tumor growth *in vivo*. During an *in vivo* xenograft mouse model of ovarian carcinoma peritoneal metastases, *SIRPA*-KO iMacs administered via IP with anti-HER2 mAb sustained the longest survival but did not reduce tumor burden in comparison to anti-HER2 mAb alone. However, when *SIRPA*-KO iMacs were administered IV with anti-HER2 mAb, tumor burden was reduced immediately in comparison to other groups, suggesting the route of iMac delivery drives antitumor activity *in vivo*. We also found that GD2-CAR-iMacs failed to control tumor growth in a xenograft mouse model of an aggressive, metastatic GD2+ neuroblastoma, while GD2-*SIRPA*-KO-CAR-iMacs significantly delayed neuroblastoma growth during the initial weeks post-treatment. However, long-term tumor delay was minimal, and there was no survival benefit compared to *SIRPA*-intact GD2-CAR iMacs; thus, combination with other neuroblastoma-directed therapies, like radiopharmaceutical therapy with ^{131}I-MIBG or other agents, may be needed.

Consistent with our observations, limited antitumor effects with CAR-iMacs have been observed in mice with pancreatic cancer and CD19-expressing tumor cells.[52,53] Improvement of CAR-iMac antitumor effects has been demonstrated after serial administration, with the first dose applied shortly after tumor injection.[52] Studies by Shen et al. revealed that tumoricidal activities of CAR-iMacs can be enforced by activation of IFN-γ signaling, with a crucial role of T cell activation noted.[53] Further longitudinal *in vivo* studies investigating CAR-iMac distribution, persistence, and intratumoral “disappearance,” along with their pro- or anti-tumoral phenotypes and secretome, will be paramount to better optimize therapeutic dosing of iMac therapies for solid tumors. In addition, recent studies demonstrated the essential role of antigen cross-presentation following treatment of tumors in immunocompetent mice with syngeneic somatic CAR-macrophages with built-in CD47 blocker using adenovirus.[47] Thus, further testing iMac therapies in humanized mouse models, along with optimization of iMac dosage, route, and frequency of administration, would be necessary to fully elucidate their utility for cancer immunotherapy and to establish their potential to activate the adaptive immune system.

(D–E) Luciferase assay was used to quantify *in vitro* tumor viability over time. PBMC-3-1 CAR or GD2-*SIRPA*-KO-CAR-iMacs were co-cultured with (D) CHLA-136 GFP-Luc2+ neuroblastoma cells or (E) WM-266-4 GFP-Luc2+ cancer cells for either 24, 48, or 96 h at a 10:1 E:T ratio. Results are mean ± SEM (n = 6); *$p < 0.05$, **$p < 0.01$, ***$p < 0.001$, two-way ANOVA.

(F) Secretome analysis of iMacs after co-culture with CHLA-136. PBMC-3-1 CAR and GD2-*SIRPA*-KO-CAR-iMacs were either cultured alone or co-cultured with CHLA-136 GFP-Luc2+ cancer cells at a 10:1 E:T ratio. After 24 h, cell culture medium was collected for secretome analysis. Results are mean ± SEM (n = 3); *$p < 0.05$, **$p < 0.01$, ***$p < 0.001$, ****$p < 0.0001$, Student’s t test.

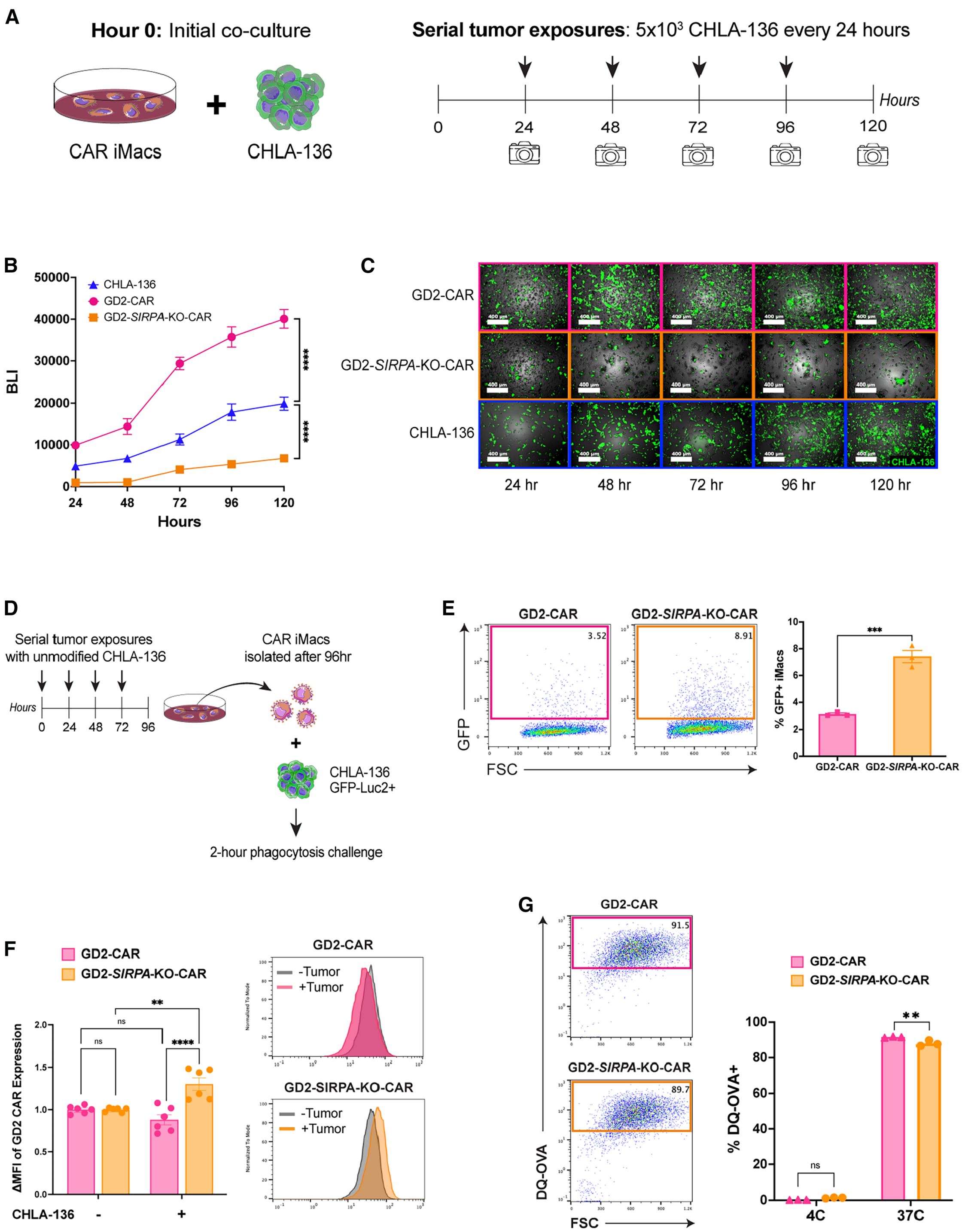

Figure 7. Ablating SIRPα in GD2-CAR-iMacs reverses CAR-mediated exhaustion

(A) Schematic for *in vitro* serial tumor exposure assay. PBMC-3-1 CAR and GD2-*SIRPA*-KO-CAR-iMacs were co-cultured with CHLA-136 GFP-Luc2+ cancer cells at an initial 10:1 E:T ratio. Every 24 h, total media were replenished with fresh CHLA-136 cells without disturbing the existing co-culture.

(B) Luciferase assay was used to detect tumor growth during *in vitro* serial tumor exposure assay at an initial 10:1 E:T ratio. Results are mean ± SEM (n = 6); ****$p < 0.0001$, two-way ANOVA.

(C) Fluorescence microscopy of GFP+ CHLA-136 neuroblastoma cells during exhaustion assay with PBMC-3-1 GD2-CAR or GD2-*SIRPA*-KO-CAR iMacs.

(D) Schematic for 2-h phagocytosis challenge post 96-h serial tumor exposure assay.

(legend continued on next page)

In conclusion, our study demonstrates the feasibility of enhancing tumoricidal activities of iPSC-derived macrophages against solid tumors through the targeted ablation of the "don't eat me" receptor SIRPα. Importantly, we revealed ablating SIRPα safeguards macrophages from hypophagic-related exhaustion against solid tumors, in both the context of mAb-driven and CAR-induced hypophagia, by the sustained expression of Fc receptors or CARs. To this end, we showed SIRPα-ablated iMacs resist exhaustion during multiple tumor exposures, enabling continuous tumor elimination and resistance to developing tumor growth-promoting activities. Furthermore, we have highlighted the utility and versatility of multiplex genetically engineered iPSC-derived SIRPα-ablated macrophages as a relevant "off-the-shelf" cellular therapy product with translational potential for the treatment of advanced adult and pediatric solid tumors.

METHODS

Human iPSC maintenance and *in vitro* hematopoietic differentiation

SIRPA-KO and WT BM9 (IISH2i-BM9-PCBC human iPSC line from WiCell) and PBMC-3-1 hiPSCs were maintained and passaged on Cultrex in mTeSR Plus media (WiCell).[28,54] Hematopoietic differentiation of *SIRPA*-KO and WT iPSCs was performed on collagen IV (ColIV)-coated plates in IF9S chemically defined serum-free medium as previously described.[33] GD2-*SIRPA-KO*-CAR and GD2-CAR iPSCs were derived from the PBMC-3-1 iPSCs or IISH2i-BM9 iPSCs. Hematopoietic differentiation of GD2-*SIRPA-KO*-CAR and GD2-CAR iPSCs was performed using a slightly modified differentiation protocol, as described previously.[28]

Generation of iPSC-macrophages

Floating hematopoietic cells from *SIRPA*-KO and WT iPSC-derived cell cultures were collected at Day 9 of differentiation and subsequently cultured in IF9S medium, as previously described,[33] with 80 ng/mL M-CSF, 50 ng/mL IL-6, and 10 ng/mL IL-3 on ultra-low attachment plates for 6 days. Fresh IF9S media (2 mL/well) was replenished after 3 days. All cells were collected after 6 days and resuspended in IF9S medium supplemented with 80 ng/mL M-CSF on uncoated tissue culture 6-well plates for an additional 4 days. Hematopoietic progenitors from GD2-*SIRPA*-KO-CAR and GD2-CAR iPSCs were induced into iMacs by treatment with E6 media, as previously described,[28] supplemented with 10% FBS, 20 ng/mL M-CSF, 10 ng/mL IL-3, and 20 ng/mL IL-6 for 3 days on uncoated tissue culture plates. All cells were collected and resuspended in fresh E6 media supplemented with 10% FBS and 100 ng/mL M-CSF for an additional 7–15 days. All cytokines are listed in Table S1.

SIRPA knockout in hiPSCs

The *SIRPA* gene was targeted for knockout at exon 3 using two sgRNAs (Synthego), listed in Table S2. Singularized iPSCs (1×10^5 cells) were electroporated with 5 mg Cas9 protein (PNA Bio #CP02) and both sgRNAs (2.5 mg of each sgRNA) using Lonza Amaxa (Program B16) and Human Stem Cell Nucleofector Starter Kit (Lonza, VPH-5002). After electroporation, cells were serially diluted into 6 well plates containing mTeSR+ media (WiCell) and CloneR supplement (STEMCELL Technologies). After 2 days, CloneR was removed, and 5–7 days later, single-cell colonies were selected and expanded for genotyping and further use.

In vivo mouse xenograft experiments

All animal experiments were performed under approval from UW-Madison, Institutional Review Board. To establish the SK-OV-3 human ovarian carcinoma xenograft mouse model, female NSG mice at 6–12 weeks old (The Jackson Laboratory) were injected with 7.5×10^6 GFP- and luciferase-expressing SK-OV-3 cells via IP injection 5 days prior to treatment. To establish the human neuroblastoma xenograft mouse model, male and female NCG-X mice at 6–12 weeks old (The Jackson Laboratory) were injected with 6×10^5 GFP- and luciferase-expressing CHLA-136 cells via tail vein IV injection 2 days prior to treatment. To assess bioluminescence of tumor burden, mice were anesthetized with isoflurane, injected with D-luciferin, and imaged by an In Vivo Imaging System (IVIS) Spectrum (PerkinElmer). Images of mice and total flux [photons/s] of tumor bioluminescence were analyzed using Living Image software. Survival time reflects the time required for animals to succumb due to tumor burden or at the time of euthanasia due to moribund status.

Statistical analysis

Data were analyzed using GraphPad Prism version 9 (GraphPad Software Inc.) and Microsoft Excel (Microsoft Corporation). Tests for statistical significance included two-sided *t* tests for paired analyses, one-way ANOVAs, and two-way ANOVAs for experiments with multiple comparisons of variables or grouped variables, accompanied by the Tukey and Sidak post hoc tests, as inferred to be most appropriate by the software.

DATA AND CODE AVAILABILITY

RNAseq data are available at GEO under the accession number GSE285814. All iPSC lines generated in this study are available from the lead contact with a completed materials transfer agreement. All other data needed to evaluate the conclusions of this paper are available in the main text or the supplementary materials.

ACKNOWLEDGMENTS

We acknowledge generous support from a St. Baldrick's Empowering Immunotherapies for Childhood Cancer grant (M.H.F. and C.M.C.) and the MACC Fund (C.M.C.). We

(E) Representative dot plot and bar graph showing phagocytosis of GFP-expressing CHLA-136 by $CD45^+$-gated CAR-iMacs. Results are mean ± SEM ($n = 3$ from two independent experiments); ***$p = 0.008$, unpaired *t* test.

(F) Surface expression of GD2-CAR 96 h after serial exposure to CHLA-136. All geometric MFI values of CAR iMacs were calculated on the $CD45^+$ population and normalized to their respective PBMC-3-1 GD2-CAR or GD2-*SIRPA*-KO-CAR iMacs alone at 96 h. Results are mean ± SEM ($n = 3$); **$p = 0.0014$, ****$p < 0.0001$, two-way ANOVA.

(G) Flow cytometric analysis of DQ-OVA uptake and digestion by PBMC-3-1 GD2-CAR or GD2-*SIRPA*-KO-CAR iMacs from 96-h serial tumor exposure cultures. Results are mean ± SEM ($n = 3$); **$p = 0.0014$, multiple paired *t* tests.

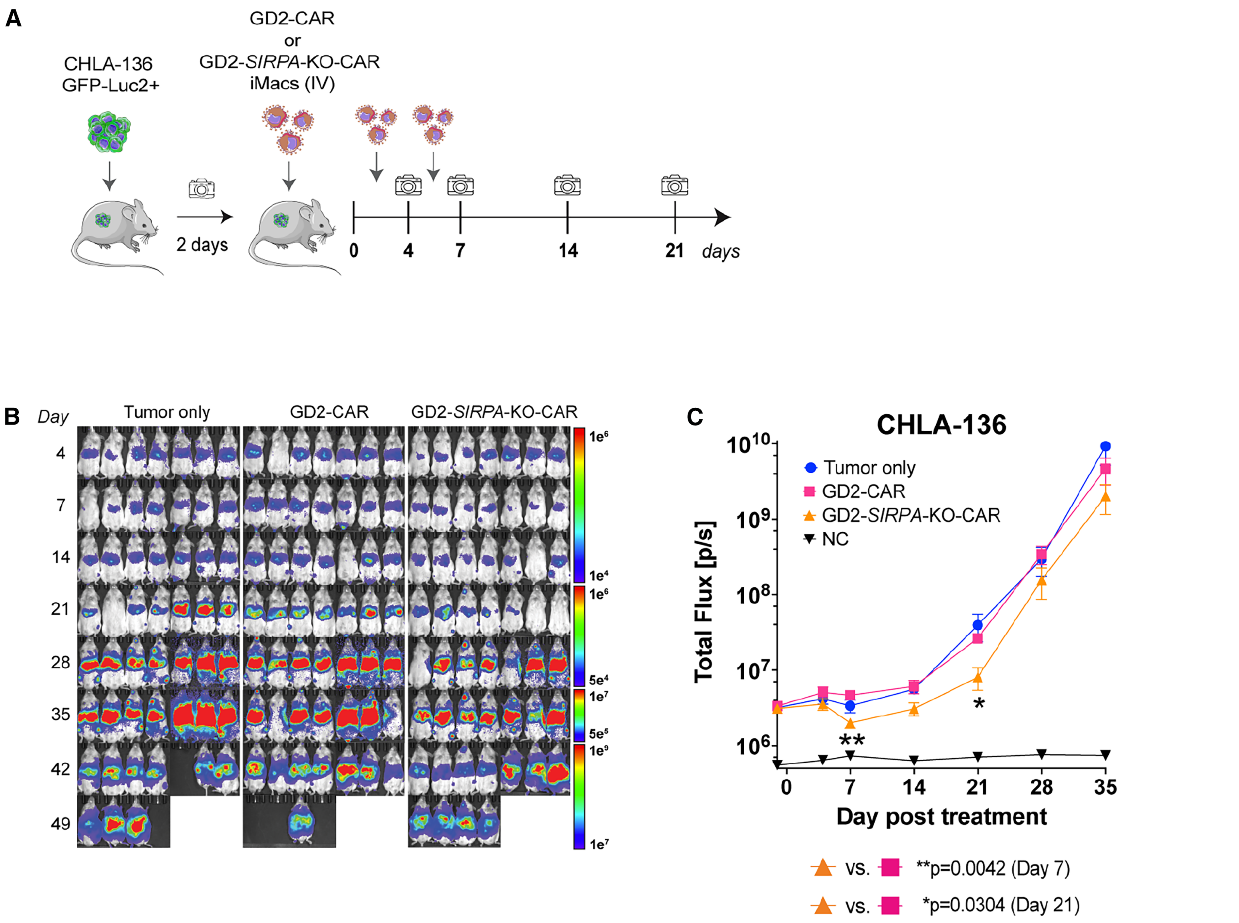

Figure 8. GD2-*SIRPA*-KO-CAR-iMacs delay tumor growth of highly metastatic neuroblastoma-engrafted mice

(A) Schematic of *in vivo* CHLA-136 tumor model establishment to generate disseminated metastases. Male and female NCG-x mice were engrafted with 6×10^5 CHLA-136 GFP-Luc2+ cancer cells via IV injection and 2 days later, treated with 5×10^6 PBMC-3-1 GD2-CAR-iMacs or GD2-*SIRPA*-KO-CAR-iMacs via IV injections on days 0, 3, and 6.

(B) Bioluminescent images of tumor xenografts over time for each treatment group. NC, negative control.

(C) Quantification of CHLA-136 tumor xenografts over time for each treatment group. NC, negative control. Results are mean total flux (photons/s) ± SEM ($n = 7$); **p*, 0.0304, ***p*, 0.0042, two-way ANOVA.

thank the National Cancer Institute Biological Resources Branch for providing 1A7 anti-14G2a antibody for the detection of GD2 CAR expression, Dr. Malcolm Brenner (Baylor College of Medicine) for sharing the GD2 CAR sequence, Dr. Vijayasaradhi Setaluri (University of Wisconsin) for providing the WM266-4 cell line, Dr. Shahab Asgharzadeh (Children's Hospital Los Angeles) for providing the CHLA-136 neuroblastoma cell line, and Ashley Weichmann (Small Animal Imaging & Radiotherapy Facility, UW-Madison) for assistance with bioluminescent imaging. The contents of this article do not necessarily reflect the views or policies of the Department of Health and Human Services nor does the mention of trade names, commercial products, or organizations imply endorsement by the US Government. This work was supported by funds from NIH/NHLBI R01 HL142665 and NIH/NHLBI U01 HL134655. WNPRC facilities are supported by NIH/OD P51 OD011106. The Flow Cytometry Laboratory, Small Animal Imaging and Radiotherapy Facility, and Data Science Resources are supported by Cancer Center support grant P30CA014520 to the University of Wisconsin (UW) Carbone Cancer Center.

AUTHOR CONTRIBUTIONS

P.R.S. designed, conducted, and analyzed experiments; interpreted experimental data; created figures; and wrote the manuscript. M.E.K. performed differentiation and flow cytometric analysis of Fc receptors and GD2-CAR, analyzed data, and generated figures. J.Z. designed and generated the GD2-CAR and GD2-*SIRPA*-KO CAR iPSC lines and performed differentiation of these cell lines. J.P.M. performed differentiation studies with CAR and CS cell lines. M.H.F. performed secretome assays and analyzed data. D.M.S. designed and generated *SIRPA*-KO-*AAVS1*-*SIRPA*-Knockin (KI) iPSCs and performed differentiation and immunoblotting. M.B. performed bioinformatics analysis and contributed to manuscript writing. J.A.T. assisted with the generation of iPSCs and engineering of CAR-iPSCs. C.M.C. assisted with the experimental design and manuscript editing. I.I.S. conceptualized, led, and supervised the studies; analyzed and interpreted data; and edited the manuscript.

DECLARATION OF INTERESTS

C.M.C. receives an honorarium for advisory board membership for Bayer, Nektar Therapeutics, and Novartis and has an equity interest in Elephas for advisory board membership. I.I.S. receives consultancy fees and holds an equity interest in Umoja Biopharma. WARF has filed patent applications based on this work, on which P.R.S., J.Z., J.A.T., and I.I.S. are listed as inventors.

SUPPLEMENTAL INFORMATION

Supplemental information can be found online at https://doi.org/10.1016/j.omton.2026.201240.

REFERENCES

1. Sica, A., Larghi, P., Mancino, A., Rubino, L., Porta, C., Totaro, M.G., Rimoldi, M., Biswas, S.K., Allavena, P., and Mantovani, A. (2008). Macrophage polarization in tumour progression. Semin. Cancer Biol. *18*, 349–355. https://doi.org/10.1016/j.semcancer.2008.03.004.

2. Christofides, A., Strauss, L., Yeo, A., Cao, C., Charest, A., and Boussiotis, V.A. (2022). The complex role of tumor-infiltrating macrophages. Nat. Immunol. *23*, 1148–1156. https://doi.org/10.1038/s41590-022-01267-2.

3. Oldenborg, P.-A., Gresham, H.D., and Lindberg, F.P. (2001). Cd47-Signal Regulatory Protein α (Sirpα) Regulates Fcγ and Complement Receptor–Mediated Phagocytosis. J. Exp. Med. *193*, 855–862.

4. Willingham, S.B., Volkmer, J.-P., Gentles, A.J., Sahoo, D., Dalerba, P., Mitra, S.S., Wang, J., Contreras-Trujillo, H., Martin, R., Cohen, J.D., et al. (2012). The CD47-signal regulatory protein alpha (SIRPa) interaction is a therapeutic target for human solid tumors. PNAS *109*, 6662–6667. https://doi.org/10.1073/pnas.1121623109.

5. Morrissey, M.A., Kern, N., and Vale, R.D. (2020). CD47 Ligation Repositions the Inhibitory Receptor SIRPA to Suppress Integrin Activation and Phagocytosis. Immunity *53*, 290–302.e6. https://doi.org/10.1016/j.immuni.2020.07.008.

6. Murata, Y., Saito, Y., Kotani, T., and Matozaki, T. (2018). CD47-signal regulatory protein α signaling system and its application to cancer immunotherapy. Cancer Sci. *109*, 2349–2357. https://doi.org/10.1111/cas.13663.

7. Okazawa, H., Motegi, S.i., Ohyama, N., Ohnishi, H., Tomizawa, T., Kaneko, Y., Oldenborg, P.-A., Ishikawa, O., and Matozaki, T. (2005). Negative regulation of phagocytosis in macrophages by the CD47-SHPS-1 system. J. Immunol. *174*, 2004–2011. https://doi.org/10.4049/jimmunol.174.4.2004.

8. Qin, J.-M., Yan, H.-X., Liu, S.-Q., Wan, X.-W., Zeng, J.-Z., Cao, H.-F., Qiu, X.-H., Wu, M.-C., and Wang, H.-Y. (2006). Negatively regulating mechanism of Sirpalpha1 in hepatocellular carcinoma: an experimental study. Hepatobiliary Pancreat. Dis. Int. *5*, 246–251.

9. Li, W. (2012). Eat-me signals: Keys to molecular phagocyte biology and "appetite" control. J. Cell. Physiol. *227*, 1291–1297. https://doi.org/10.1002/jcp.22815.

10. Eladl, E., Tremblay-LeMay, R., Rastgoo, N., Musani, R., Chen, W., Liu, A., and Chang, H. (2020). Role of CD47 in Hematological Malignancies. J. Hematol. Oncol. *13*, 96. https://doi.org/10.1186/s13045-020-00930-1.

11. Yuan, J., He, H., Chen, C., Wu, J., Rao, J., and Yan, H. (2019). Combined high expression of CD47 and CD68 is a novel prognostic factor for breast cancer patients. Cancer Cell Int. *19*, 238. https://doi.org/10.1186/s12935-019-0957-0.

12. Li, Y., Lu, S., Xu, Y., Qiu, C., Jin, C., Wang, Y., Liu, Z., and Kong, B. (2017). Overexpression of CD47 predicts poor prognosis and promotes cancer cell invasion in high-grade serous ovarian carcinoma. Am. J. Transl. Res. *9*, 2901–2910.

13. Yang, M., Jiang, C., Li, L., Xing, H., and Hong, L. (2022). Expression of CD47 in Endometrial Cancer and Its Clinicopathological Significance. J. Oncol. *2022*, 1–10. https://doi.org/10.1155/2022/7188972.

14. Yoshida, K., Tsujimoto, H., Matsumura, K., Kinoshita, M., Takahata, R., Matsumoto, Y., Hiraki, S., Ono, S., Seki, S., Yamamoto, J., and Hase, K. (2015). CD47 is an adverse prognostic factor and a therapeutic target in gastric cancer. Cancer Med. *4*, 1322–1333. https://doi.org/10.1002/cam4.478.

15. Shi, M., Gu, Y., Jin, K., Fang, H., Chen, Y., Cao, Y., Liu, X., Lv, K., He, X., Lin, C., et al. (2021). CD47 expression in gastric cancer clinical correlates and association with macrophage infiltration. Cancer Immunol. Immunother. *70*, 1831–1840. https://doi.org/10.1007/s00262-020-02806-2.

16. Xu, Y., Li, J., Tong, B., Chen, M., Liu, X., Zhong, W., Zhao, J., and Wang, M. (2020). Positive tumour CD47 expression is an independent prognostic factor for recurrence in resected non-small cell lung cancer. ESMO Open *5*, e000823. https://doi.org/10.1136/esmoopen-2020-000823.

17. Park, H., Jee, S., Bang, S., Son, H., Cha, H., Myung, J., Sim, J., Kim, Y., Paik, S., and Kim, H. (2022). CD47 Expression Predicts Unfavorable Prognosis in Clear Cell Renal Cell Carcinoma after Curative Resection. Diagnostics (Basel) *12*, 2291. https://doi.org/10.3390/diagnostics12102291.

18. Chao, M.P., Alizadeh, A.A., Tang, C., Myklebust, J.H., Varghese, B., Gill, S., Jan, M., Cha, A.C., Chan, C.K., Tan, B.T., et al. (2010). Anti-CD47 antibody synergizes with rituximab to promote phagocytosis and eradicate non-Hodgkin lymphoma. Cell *142*, 699–713. https://doi.org/10.1016/j.cell.2010.07.044.

19. Sikic, B.I., Lakhani, N., Patnaik, A., Shah, S.A., Chandana, S.R., Rasco, D., Colevas, A.D., O'Rourke, T., Narayanan, S., Papadopoulos, K., et al. (2019). First-in-Human, First-in-Class Phase I Trial of the Anti-CD47 Antibody Hu5F9-G4 in Patients With Advanced Cancers. J. Clin. Oncol. *37*, 946–953. https://doi.org/10.1200/JCO.18.02018.

20. Xu, Z., Gao, J., Yao, J., Yang, T., Wang, D., Dai, C., and Ding, Y. (2021). Preclinical efficacy and toxicity studies of a highly specific chimeric anti-CD47 antibody. FEBS Open Bio *11*, 813–825. https://doi.org/10.1002/2211-5463.13084.

21. Petrova, P.S., Viller, N.N., Wong, M., Pang, X., Lin, G.H.Y., Dodge, K., Chai, V., Chen, H., Lee, V., House, V., et al. (2017). TTI-621 (SIRPαFc): A CD47-Blocking Innate Immune Checkpoint Inhibitor with Broad Antitumor Activity and Minimal Erythrocyte Binding. Clin. Cancer Res. *23*, 1068–1079. https://doi.org/10.1158/1078-0432.CCR-16-1700.

22. Barkal, A.A., Brewer, R.E., Markovic, M., Kowarsky, M., Barkal, S.A., Zaro, B.W., Krishnan, V., Hatakeyama, J., Dorigo, O., Barkal, L.J., and Weissman, I.L. (2019). CD24 signalling through macrophage Siglec-10 is a target for cancer immunotherapy. Nature *572*, 392–396. https://doi.org/10.1038/s41586-019-1456-0.

23. Advani, R., Flinn, I., Popplewell, L., Forero, A., Bartlett, N.L., Ghosh, N., Kline, J., Roschewski, M., LaCasce, A., Collins, G.P., et al. (2018). CD47 Blockade by Hu5F9-G4 and Rituximab in Non-Hodgkin's Lymphoma. N. Engl. J. Med. *379*, 1711–1721. https://doi.org/10.1056/NEJMoa1807315.

24. Isenberg, J.S., Romeo, M.J., Yu, C., Yu, C.K., Nghiem, K., Monsale, J., Rick, M.E., Wink, D.A., Frazier, W.A., and Roberts, D.D. (2008). Thrombospondin-1 stimulates platelet aggregation by blocking the antithrombotic activity of nitric oxide/cGMP signaling. Blood *111*, 613–623. https://doi.org/10.1182/blood-2007-06-098392.

25. Roberts, D.D., Miller, T.W., Rogers, N.M., Yao, M., and Isenberg, J.S. (2012). The matricellular protein thrombospondin-1 globally regulates cardiovascular function and responses to stress via CD47. Matrix Biol. *31*, 162–169. https://doi.org/10.1016/j.matbio.2012.01.005.

26. Klichinsky, M., Ruella, M., Shestova, O., Lu, X.M., Best, A., Zeeman, M., Schmierer, M., Gabrusiewicz, K., Anderson, N.R., Petty, N.E., et al. (2020). Human chimeric antigen receptor macrophages for cancer immunotherapy. Nat. Biotechnol. *38*, 947–953. https://doi.org/10.1038/s41587-020-0462-y.

27. Zhang, L., Tian, L., Dai, X., Yu, H., Wang, J., Lei, A., Zhu, M., Xu, J., Zhao, W., Zhu, Y., et al. (2020). Pluripotent stem cell-derived CAR-macrophage cells with antigen-dependent anti-cancer cell functions. J. Hematol. Oncol. *13*, 153. https://doi.org/10.1186/s13045-020-00983-2.

28. Zhang, J., Webster, S., Duffin, B., Bernstein, M.N., Steill, J., Swanson, S., Forsberg, M.H., Bolin, J., Brown, M.E., Majumder, A., et al. (2023). Generation of anti-GD2 CAR macrophages from human pluripotent stem cells for cancer immunotherapies. Stem Cell Rep. *18*, 585–596. https://doi.org/10.1016/j.stemcr.2022.12.012.

29. Hatherley, D., Graham, S.C., Turner, J., Harlos, K., Stuart, D.I., and Barclay, A.N. (2008). Paired Receptor Specificity Explained by Structures of Signal Regulatory Proteins Alone and Complexed with CD47. Mol. Cell *31*, 266–277. https://doi.org/10.1016/j.molcel.2008.05.026.

30. Barclay, A.N., and Van den Berg, T.K. (2014). The interaction between signal regulatory protein alpha (SIRPα) and CD47: structure, function, and therapeutic target. Annu. Rev. Immunol. *32*, 25–50. https://doi.org/10.1146/annurev-immunol-032713-120142.

31. Hatherley, D., Harlos, K., Dunlop, D.C., Stuart, D.I., and Barclay, A.N. (2007). The Structure of the Macrophage Signal Regulatory Protein α (SIRPα) Inhibitory Receptor Reveals a Binding Face Reminiscent of That Used by T Cell Receptors. J. Biol. Chem. *282*, 14567–14575. https://doi.org/10.1074/jbc.M611511200.

32. Huang, B., Bai, Z., Ye, X., Zhou, C., Xie, X., Zhong, Y., Lin, K., and Ma, L. (2021). Structural analysis and binding sites of inhibitors targeting the CD47/SIRPα interaction in anticancer therapy. Comput. Struct. Biotechnol. J. *19*, 5494–5503. https://doi.org/10.1016/j.csbj.2021.09.036.

33. Uenishi, G., Theisen, D., Lee, J.-H., Kumar, A., Raymond, M., Vodyanik, M., Swanson, S., Stewart, R., Thomson, J., and Slukvin, I. (2014). Tenascin C Promotes Hematoendothelial Development and T Lymphoid Commitment from Human Pluripotent Stem Cells in Chemically Defined Conditions. Stem Cell Rep. *3*, 1073–1084. https://doi.org/10.1016/j.stemcr.2014.09.014.

34. Hume, D.A., Millard, S.M., and Pettit, A.R. (2023). Macrophage heterogeneity in the single-cell era: facts and artifacts. Blood *142*, 1339–1347. https://doi.org/10.1182/blood.2023020597.

35. Liu, L., Ye, Y., and Zhu, X. (2019). MMP-9 secreted by tumor associated macrophages promoted gastric cancer metastasis through a PI3K/AKT/Snail pathway. Biomed. Pharmacother. *117*, 109096. https://doi.org/10.1016/j.biopha.2019.109096.

36. Pelekanou, V., Villarroel-Espindola, F., Schalper, K.A., Pusztai, L., and Rimm, D.L. (2018). CD68, CD163, and matrix metalloproteinase 9 (MMP-9) co-localization in breast tumor microenvironment predicts survival differently in ER-positive and -negative cancers. Breast Cancer Res. *20*, 154. https://doi.org/10.1186/s13058-018-1076-x.

37. Xu, J., Yu, Y., He, X., Niu, N., Li, X., Zhang, R., Hu, J., Ma, J., Yu, X., Sun, Y., et al. (2019). Tumor-associated macrophages induce invasion and poor prognosis in human gastric cancer in a cyclooxygenase-2/MMP9-dependent manner. Am. J. Transl. Res. *11*, 6040.

38. Yue, Y., Huang, W., Liang, J., Guo, J., Ji, J., Yao, Y., Zheng, M., Cai, Z., Lu, L., and Wang, J. (2015). IL4I1 Is a Novel Regulator of M2 Macrophage Polarization That Can Inhibit T Cell Activation via L-Tryptophan and Arginine Depletion and IL-10 Production. PLoS ONE *10*, e0142979. https://doi.org/10.1371/journal.pone.0142979.

39. Sadik, A., Somarribas Patterson, L.F., Öztürk, S., Mohapatra, S.R., Panitz, V., Secker, P.F., Pfänder, P., Loth, S., Salem, H., Prentzell, M.T., et al. (2020). IL4I1 Is a Metabolic Immune Checkpoint that Activates the AHR and Promotes Tumor Progression. Cell *182*, 1252–1270.e34. https://doi.org/10.1016/j.cell.2020.07.038.

40. Vekariya, U., Rawat, K., Saxena, R., and Tripathi, R.K. (2019). Identification of MΦ specific POTEE expression: Its role in mTORC2 activation via protein-protein interaction in TAMs. Cell. Immunol. *335*, 30–40. https://doi.org/10.1016/j.cellimm.2018.10.010.

41. Pinney, J.J., Rivera-Escalera, F., Chu, C.C., Whitehead, H.E., VanDerMeid, K.R., Nelson, A.M., Barbeau, M.C., Zent, C.S., and Elliott, M.R. (2020). Macrophage hypophagia as a mechanism of innate immune exhaustion in mAb-induced cell clearance. Blood *136*, 2065–2079. https://doi.org/10.1182/blood.2020005571.

42. Nazha, B., Inal, C., and Owonikoko, T.K. (2020). Disialoganglioside GD2 Expression in Solid Tumors and Role as a Target for Cancer Therapy. Front. Oncol. *10*, 1000. https://doi.org/10.3389/fonc.2020.01000.

43. Alvey, C.M., Spinler, K.R., Irianto, J., Pfeifer, C.R., Hayes, B., Xia, Y., Cho, S., Dingal, P.C.P.D., Hsu, J., Smith, L., et al. (2017). SIRPA-Inhibited, Marrow-Derived Macrophages Engorge, Accumulate, and Differentiate in Antibody-Targeted Regression of Solid Tumors. Curr. Biol. *27*, 2065–2077.e6. https://doi.org/10.1016/j.cub.2017.06.005.

44. Bian, Z., Shi, L., Kidder, K., Zen, K., Garnett-Benson, C., and Liu, Y. (2021). Intratumoral SIRPα-deficient macrophages activate tumor antigen-specific cytotoxic T cells under radiotherapy. Nat. Commun. *12*, 3229. https://doi.org/10.1038/s41467-021-23442-z.

45. Zhang, H., Huo, Y., Zheng, W., Li, P., Li, H., Zhang, L., Sa, L., He, Y., Zhao, Z., Shi, C., et al. (2024). Silencing of SIRPα enhances the antitumor efficacy of CAR-M in solid tumors. Cell. Mol. Immunol. *21*, 1335–1349. https://doi.org/10.1038/s41423-024-01220-3.

46. Chen, S., Wang, Y., Dang, J., Song, N., Chen, X., Wang, J., Huang, G.N., Brown, C.E., Yu, J., Weissman, I.L., et al. (2025). CAR macrophages with built-In CD47 blocker combat tumor antigen heterogeneity and activate T cells via cross-presentation. Nat. Commun. *16*, 4069. https://doi.org/10.1038/s41467-025-59326-9.

47. Huang, Y., Lv, S.-Q., Liu, P.-Y., Ye, Z.-L., Yang, H., Li, L.-F., Zhu, H.-L., Wang, Y., Cui, L.-Z., Jiang, D.-Q., et al. (2020). A SIRPα-Fc fusion protein enhances the antitumor effect of oncolytic adenovirus against ovarian cancer. Mol. Oncol. *14*, 657–668. https://doi.org/10.1002/1878-0261.12628.

48. Augoff, K., Hryniewicz-Jankowska, A., Tabola, R., and Stach, K. (2022). MMP9: A Tough Target for Targeted Therapy for Cancer. Cancers *14*, 1847. https://doi.org/10.3390/cancers14071847.

49. Pradhan, K., Yi, Z., Geng, S., and Li, L. (2021). Development of Exhausted Memory Monocytes and Underlying Mechanisms. Front. Immunol. *12*, 778830. https://doi.org/10.3389/fimmu.2021.778830.

50. Liu, S., Yin, W., Lin, Y., Huang, S., Xue, S., Sun, G., and Wang, C. (2023). Metastasis pattern and prognosis in children with neuroblastoma. World J. Surg. Oncol. *21*, 130. https://doi.org/10.1186/s12957-023-03011-y.

51. Louis, C.U., and Shohet, J.M. (2015). Neuroblastoma: Molecular Pathogenesis and Therapy. Annu. Rev. Med. *66*, 49–63. https://doi.org/10.1146/annurev-med-011514-023121.

52. Shah, Z., Tian, L., Li, Z., Jin, L., Zhang, J., Li, Z., Barr, T., Tang, H., Feng, M., Caligiuri, M.A., and Yu, J. (2024). Human anti-PSCA CAR macrophages possess potent antitumor activity against pancreatic cancer. Cell Stem Cell *31*, 803–817.e6. https://doi.org/10.1016/j.stem.2024.03.018.

53. Shen, J., Lyu, S., Xu, Y., Zhang, S., Li, L., Li, J., Mou, J., Xie, L., Tang, K., Wen, W., et al. (2024). Activating innate immune responses repolarizes hPSC-derived CAR macrophages to improve anti-tumor activity. Cell Stem Cell *31*, 1003–1019.e9. https://doi.org/10.1016/j.stem.2024.04.012.

54. Hu, K., Yu, J., Suknuntha, K., Tian, S., Montgomery, K., Choi, K.-D., Stewart, R., Thomson, J.A., and Slukvin, I.I. (2011). Efficient generation of transgene-free induced pluripotent stem cells from normal and neoplastic bone marrow and cord blood mononuclear cells. Blood *117*, e109–e119. https://doi.org/10.1182/blood-2010-07-298331.

OMTON, Volume *34*

Supplemental information

SIRPα ablated iPSC-derived macrophages resist hypophagia and enhance mAb-dependent and CAR-mediated cytotoxicity of solid tumors

Portia R. Smith, Md Ehsanul Kabir, Jue Zhang, John P. Maufort, Matthew H. Forsberg, Divine M. Sedzro, Mark Berres, James A. Thomson, Christian M. Capitini, and Igor I. Slukvin

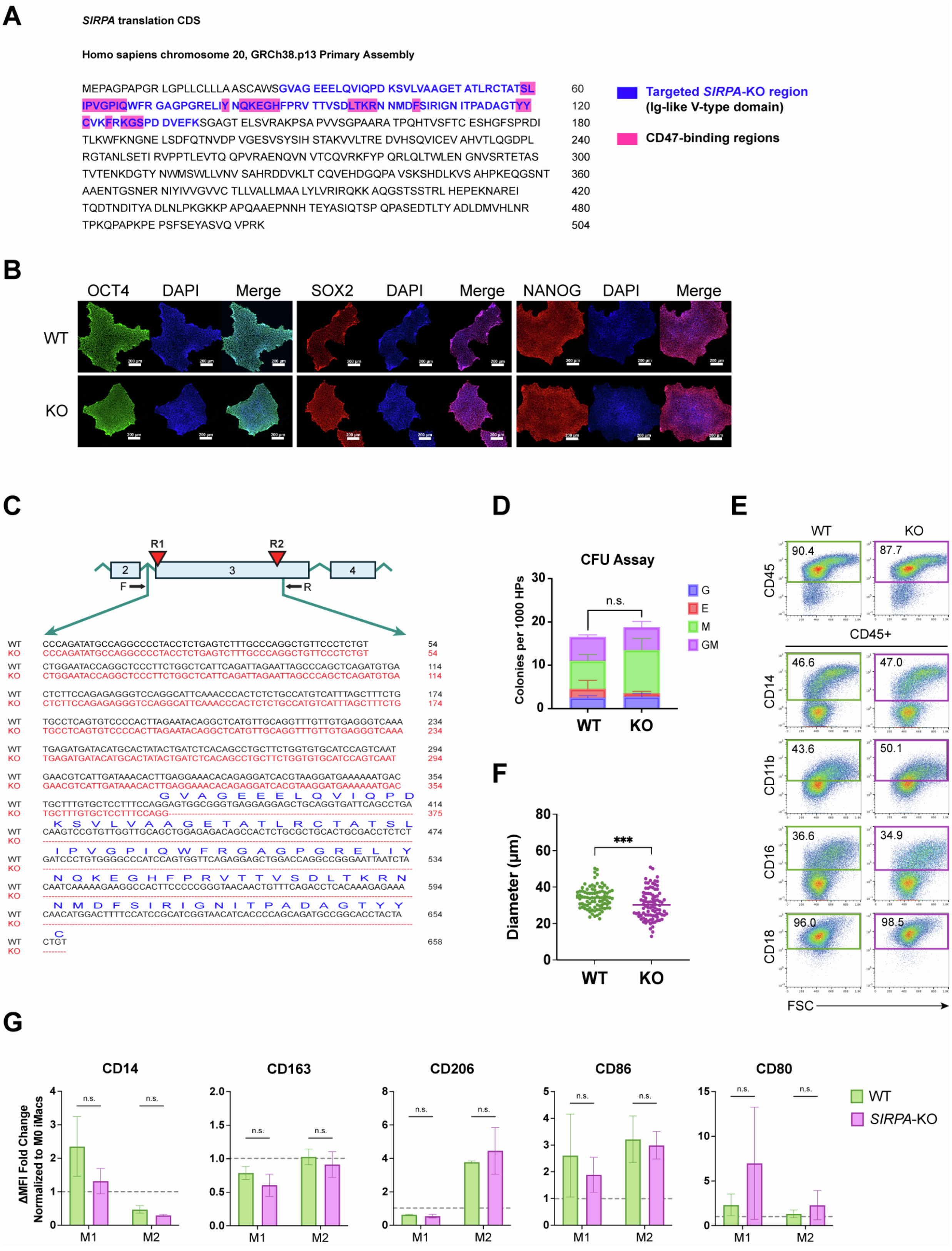
A
SIRPA translation CDS
Homo sapiens chromosome 20, GRCh38.p13 Primary Assembly
MEPAGPAPGR LGPLLCLLLA ASCAWSGVAG EEELQVIQPD KSVLVAAGET ATLRCTATSL 60
IPVGPIQWFR GAGPGRELIY NQKEGHFPRV TTVSDLTKRN NMDFSIRIGN ITPADAGTYY 120
CVKFRKGSPD DVEFKSGAGT ELSVRAKPSA PVVSGPAARA TPQHTVSFTC ESHGFSPRDI 180
TLKWFKNGNE LSDFQTNVDP VGESVSYSIH STAKVVLTRE DVHSQVICEV AHVTLQGDPL 240
RGTANLSETI RVPPTLEVTQ QPVRAENQVN VTCQVRKFYP QRLQLTWLEN GNVSRTETAS 300
TVTENKDGTY NWMSWLLVNV SAHRDDVKLT CQVEHDGQPA VSKSHDLKVS AHPKEQGSNT 360
AAENTGSNER NIYIVVGVVC TLLVALLMAA LYLVRIRQKK AQGSTSSTRL HEPEKNAREI 420
TQDTNDITYA DLNLPKGKKP APQAAEPNNH TEYASIQTSP QPASEDTLTY ADLDMVHLNR 480
TPKQPAPKPE PSFSEYASVQ VPRK 504
Targeted SIRPA-KO region (Ig-like V-type domain)
CD47-binding regions
B
OCT4 DAPI Merge SOX2 DAPI Merge NANOG DAPI Merge
WT
KO
C
R1 R2
2 3 4
F R
D
CFU Assay
Colonies per 1000 HPs
n.s.
G E M GM
WT KO
E
WT KO
CD45 90.4 87.7
CD45+
CD14 46.6 47.0
CD11b 43.6 50.1
CD16 36.6 34.9
CD18 96.0 98.5
FSC
F
Diameter (µm)
***
WT KO
G
CD14 CD163 CD206 CD86 CD80
ΔMFI Fold Change Normalized to M0 iMacs
n.s.
M1 M2
WT
SIRPA-KO

Figure S1. Characterization of SIRPA-KO iPSC line. (A) Human *SIRPA* gene translation CDS generated in Geneious Prime software. Amino acid sequence in blue text is the approximate knockout target region using two sgRNAs flanking *SIRPA* exon 3. Amino acids highlighted in pink represent CD47-binding motifs within SIRPα protein.[1,2] (B) Immunofluorescence of OCT4, SOX2, and NANOG expression on WT and *SIRPA*-KO (KO) iPSCs. (C) Nanopore sequencing of genomic PCR product from WT and *SIRPA*-KO iPSCs amplified with primers (black arrows) to target *SIRPA* exon 3 described in Table S2. Red triangles R1 and R2 indicate *SIRPA*-KO sgRNAs 1 and 2, respectively. Nucleotide sequence of WT iPSCs is denoted in black text, and SIRPA-KO iPSCs in red text. Predicted amino acid translation of WT exon 3 is denoted in large blue lettering. (D) Colony forming unit (CFU) assay from WT and *SIRPA*-KO iPSC-derived day 9 floating HPs. Data are represented as mean ± SEM (WT n=2; KO n=4) n.s. $p>0.05$, multiple unpaired t tests. (E) Flow cytometric analysis of myeloid cells after culture of day 9 floating HPs with M-CSF, IL-3 and IL-6 for 6 days. (F) WT and *SIRPA*-KO iPSC-derived macrophages (iMacs) were stained with Wright-Giemsa and imaged using brightfield microscopy. iMac diameters were measured as the maximum cell width (widest point) from brightfield images using Image J.JS analysis. Data are represented as mean and individual points (n=90 per group). ***$p = 0.0004$, Welch's t test. (G) Expression of CD14, CD163, CD206, CD86, and CD80 in WT and *SIRPA*-KO iMacs stimulated for 48 hours with IFN-γ + LPS (M1), IL-4 (M2), or unstimulated (M0) and unstained (Uns.) macrophages, which were used as a control. Data are represented as mean ± SEM (n=3), n.s. $p>0.05$, multiple paired t-tests.

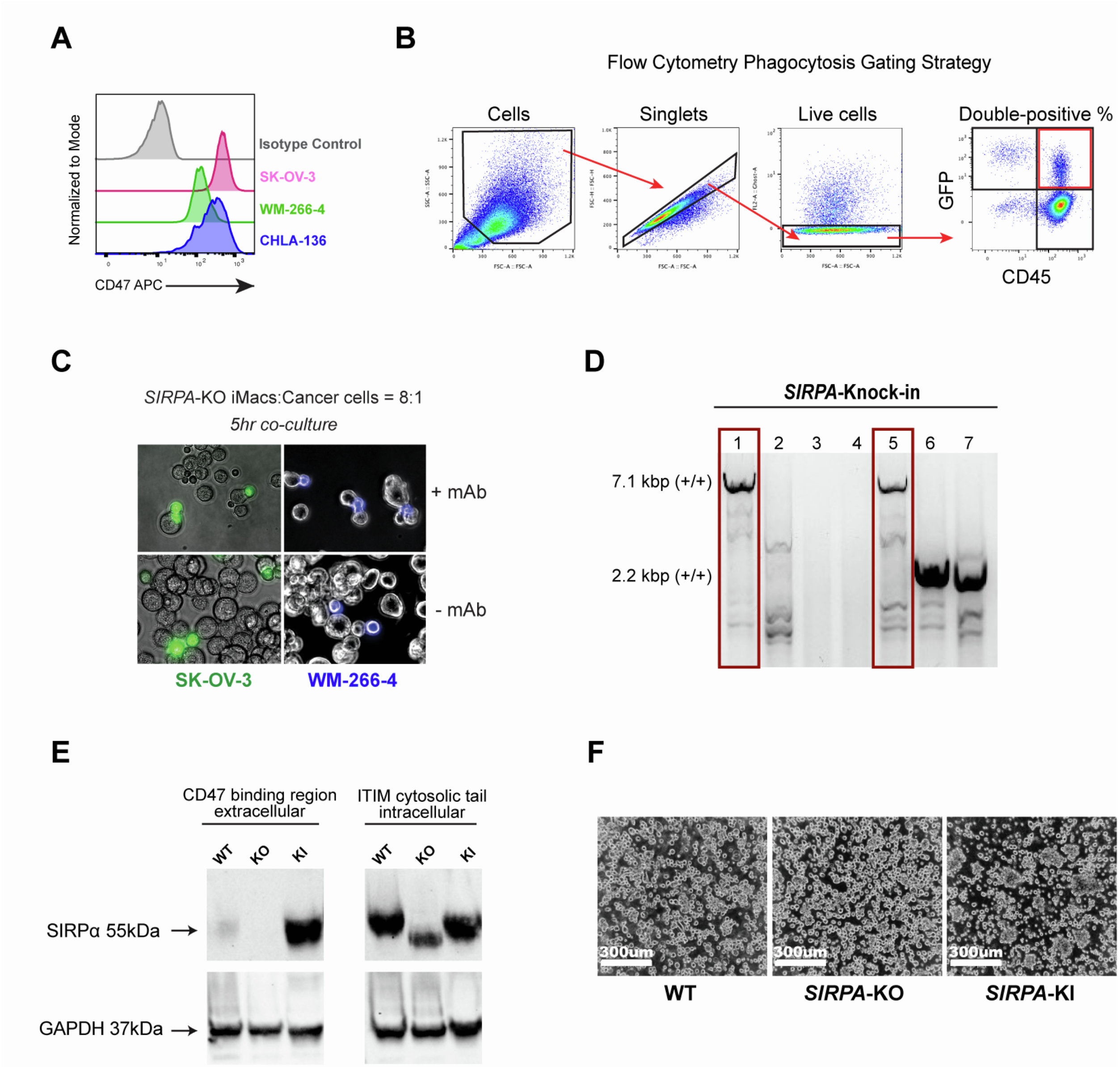

Figure S2. Characterization of tumor cell lines and *SIRPA* KO and Knock-in (KI) cell lines. (A) Flow cytometric analysis of human CD47 expression on cancer cell lines used in this study. **(B)** Representative flow cytometry gating schematic for antibody-dependent phagocytosis assay. Engulfment index was calculated as (# Double-positive GFP+CD45+ cells) / (Total # of GFP+ cells) x 100. **(C)** Fluorescence microscopy images of WT or *SIRPA*-KO iMacs co-cultured with various cancer cell lines with or without mAb; SK-OV-3 +/- anti-HER2, WM-266-4 +/- anti-GD3. **(D)** Genomic PCR of *SIRPA*-Knock-in within the AAVS1 locus of *SIRPA*-KO iPSCs. **(E)** Western blot of human SIRPα protein (55kDa) and GAPDH protein (37kDa) within WT, *SIRPA*-KO (KO), or *SIRPA*-KI (KI) iMacs. **(F)** Phase contrast microscopy images of WT, *SIRPA*-KO, or *SIRPA*-KI iMac cell cultures.

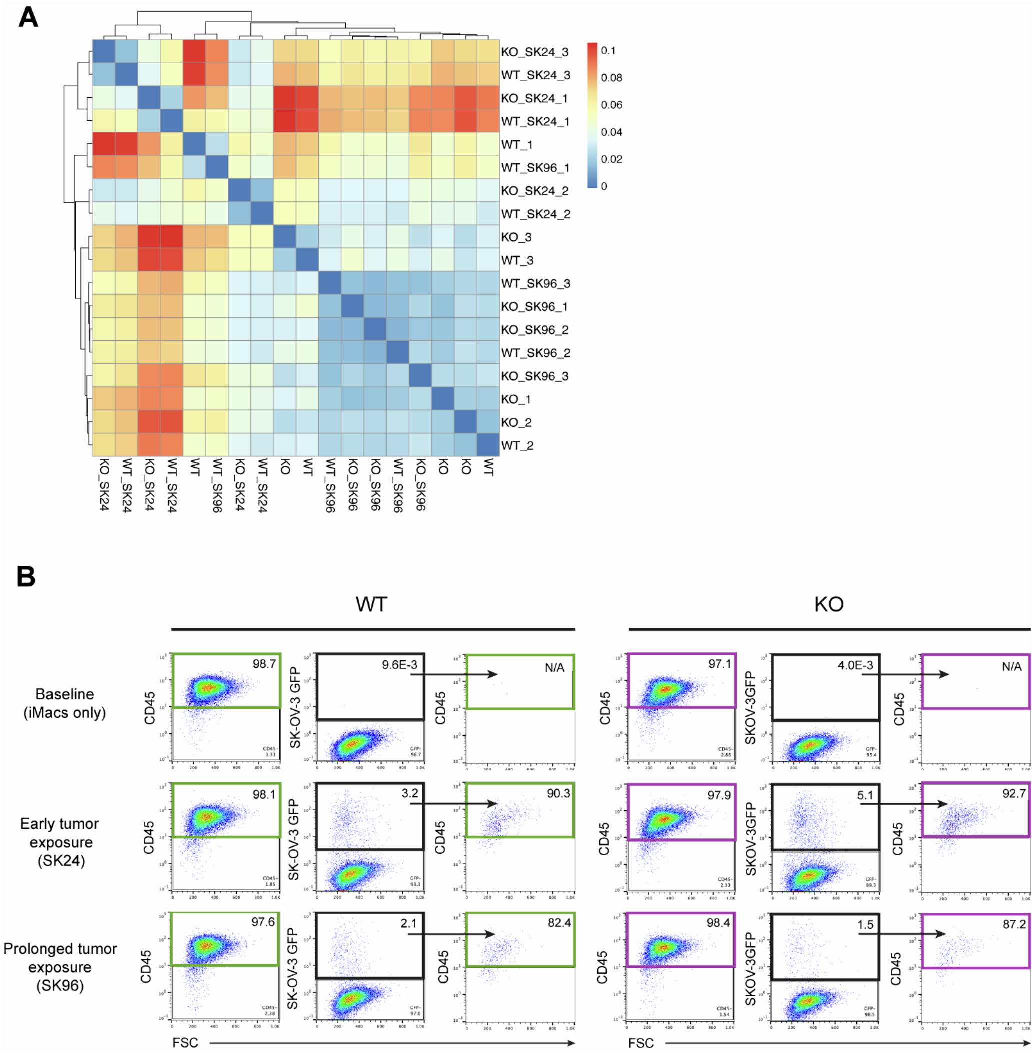

Figure S3. RNA-sequencing sample validation and quality control of WT and *SIRPA*-KO iMacs. **(A)** Sample level correlation heatmap of RNA-sequencing data with hierarchal clustering. Heatmap shows pairwise correlation differences (1 – correlation) between individual RNA-sequencing samples from WT or *SIRPA*-KO iMacs under baseline conditions, 'early' 24hr tumor exposure (SK24), or 'prolonged' 96hr tumor exposure (SK96). **(B)** Flow cytometric validation of CD45+ post-magnetic bead sorting of WT or *SIRPA*-KO iMacs under baseline conditions, 'early' 24hr tumor exposure (SK24), or 'prolonged' 96hr tumor exposure (SK96). Representative flow cytometry plots of each treatment group show CD45 and GFP expression after CD45+ magnetic bead sorting enrichment prior to total RNA isolation.

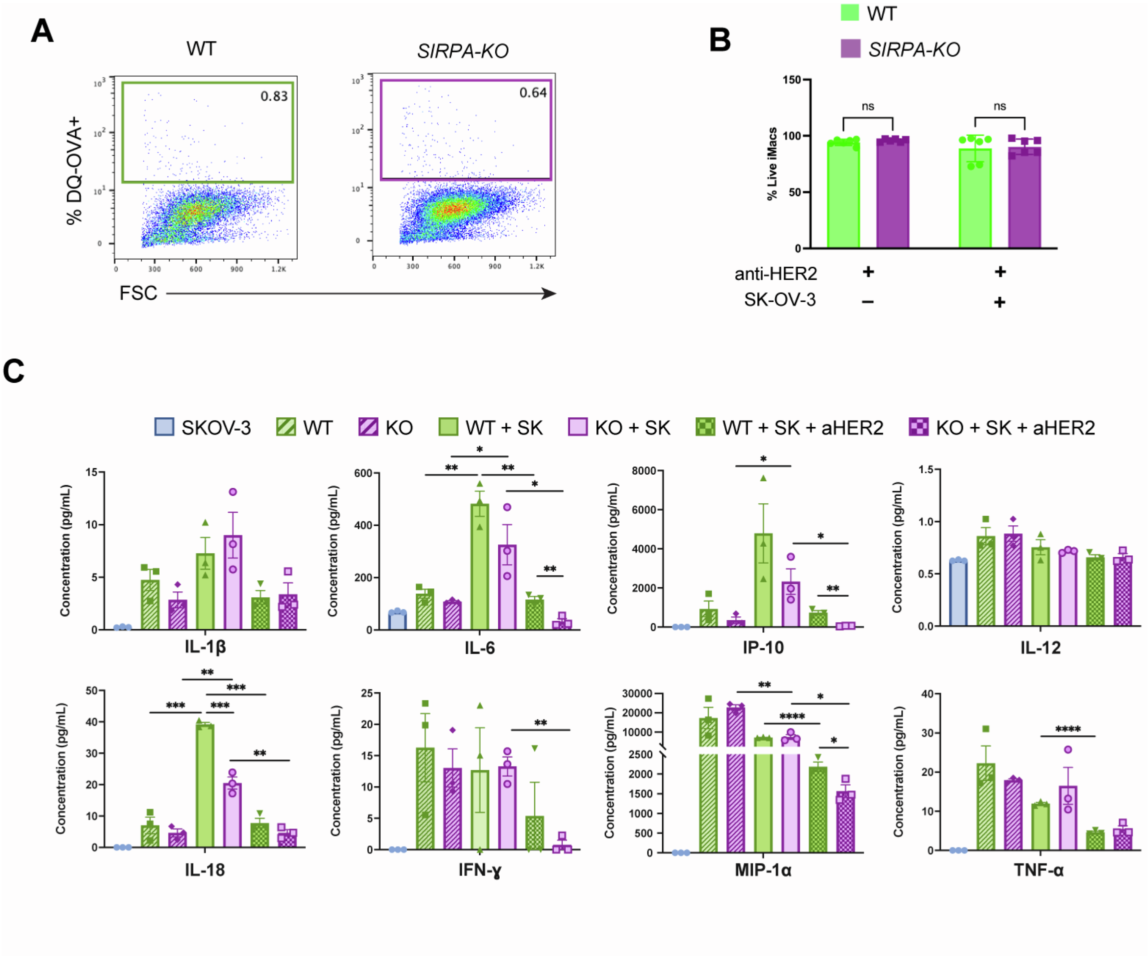

Figure S4. Characterization of *SIRPA*-KO iMacs after serial exposure to the tumor. (A) Representative dot plots show DQ-OVA fluorescence of iMacs from 96 hours serial tumor exposure following 30 minutes of exposure to DQ-OVA at 4^{0}C. **(B)** Viability of CD45$^+$gated iMacs from 96 hours serial tumor exposure cultures with and without the addition of anti-HER2 or SK-OV-3. **(C)** Secretome analysis of iMacs from 96 hours serial tumor exposure cultures. WT and *SIRPA*-KO iMacs were co-cultured with SK-OV-3 GFP-Luc2+ cancer cells +/- anti-HER2 at a 20:1 effector-to-target ratio. Every 24 hours, total media was replenished with fresh SK-OV-3 +/- anti-HER without disturbing the existing co-culture. At 96 hours, cell culture media of the co-cultures were collected for secretome analysis. Results are mean ± SEM (n=3); *p<0.05, **p<0.01, ***p<0.001, ****p<0.0001, student's t test.

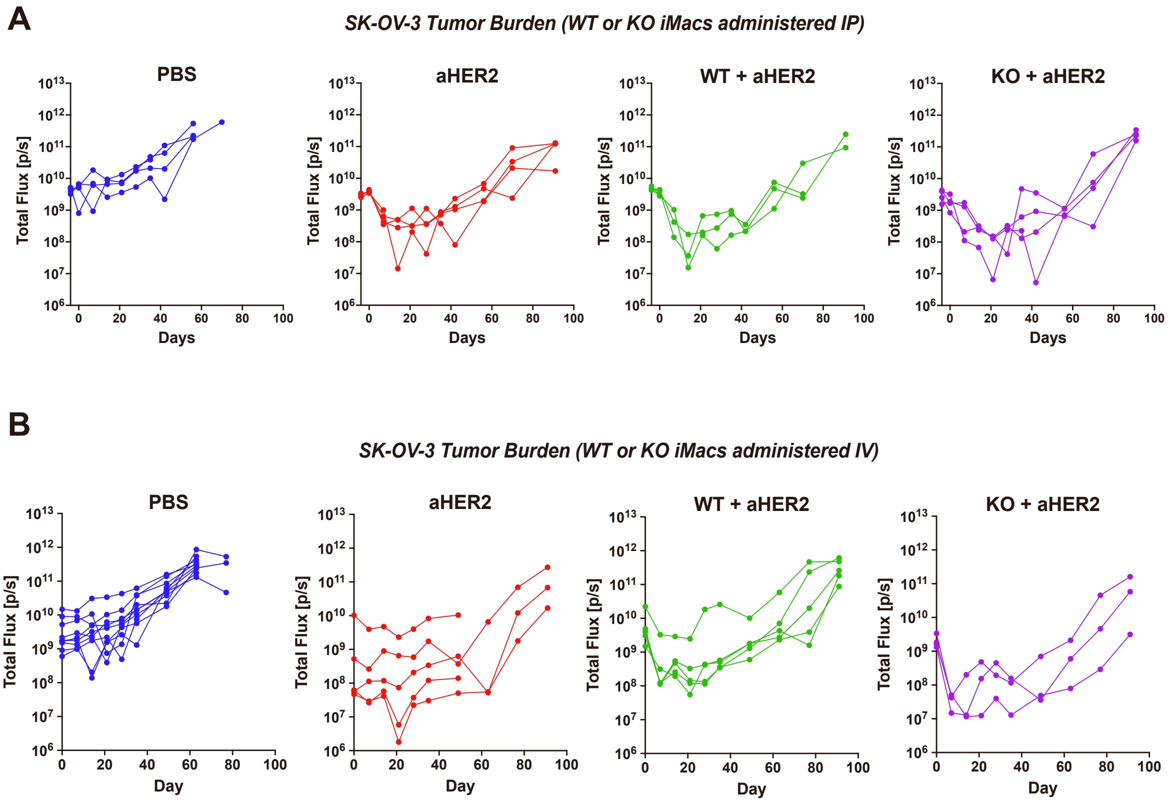

Figure S5. Quantification of tumor burden in individual mice. (A) Quantification of SK-OV-3 tumor xenografts over time for each treatment group after IP injection of iMacs. Results are total flux [photons/s] of tumor burden of each individual mouse from each group (n=4). **(B)** Quantification of SK-OV-3 tumor xenografts over time for each treatment group after IV injection of iMacs. Results are total flux [photons/s] of tumor burden of each individual mouse from each group (PBS n=8, anti-HER2 n=5, WT + aHER2 n=5, KO + aHER2 n=3).

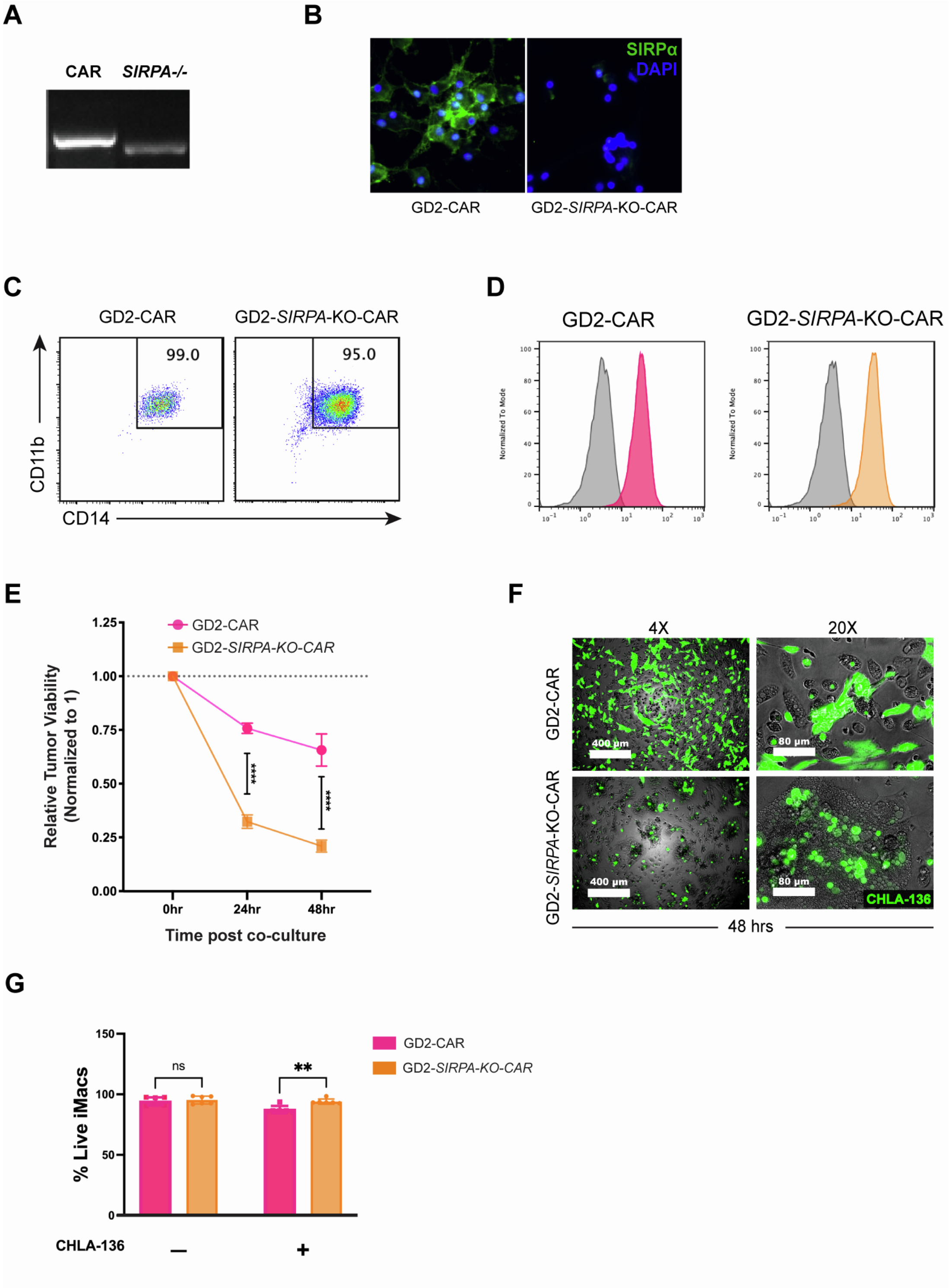

Figure S6. Characterization of the impact of *SIRPA* KO on GD2-CAR iMacs. (A) Genomic PCR of successful *SIRPA*-knockout in GD2-*SIRPA*-KO PBMC-3-1 iPSCs. **(B)** PBMC-3-1 GD2-CAR and GD2-

SIRPA-KO-CAR iMacs were stained for DAPI (blue) and anti-SIRPα antibody (green) and subject to fluorescence microscopy. **(C)** PBMC-3-1 GD2-CAR and GD2-*SIRPA*-KO-CAR iMacs were stained for CD14 and CD11b in flow cytometric analysis. **(D)** PBMC-3-1 WT (grey), GD2-CAR (pink) and GD2-*SIRPA*-KO-CAR (orange) iMacs were stained for an antibody that recognizes anti-GD2-CAR expression (1A7) in flow cytometric analysis. **(E)** IISH2i-BM9 CAR or GD2-*SIRPA*-KO iMacs were co-cultured with CHLA-136 GFP-Luc2+ neuroblastoma cells at 1:1 E:T ratio for 0, 24 or 48 hours. Results are shown as mean ± SEM (n=6); ****p<0.0001, two-way ANOVA. **(F)** Fluorescent microscopy images of GFP+ CHLA-136 viable cells during in vitro serial tumor exposure assay at 4X and 20X magnification. **(G)** Viability of CAR-iMacs in 96 hours serial tumor exposure cultures. Results are mean ± SE (n=6). **p<0.01.

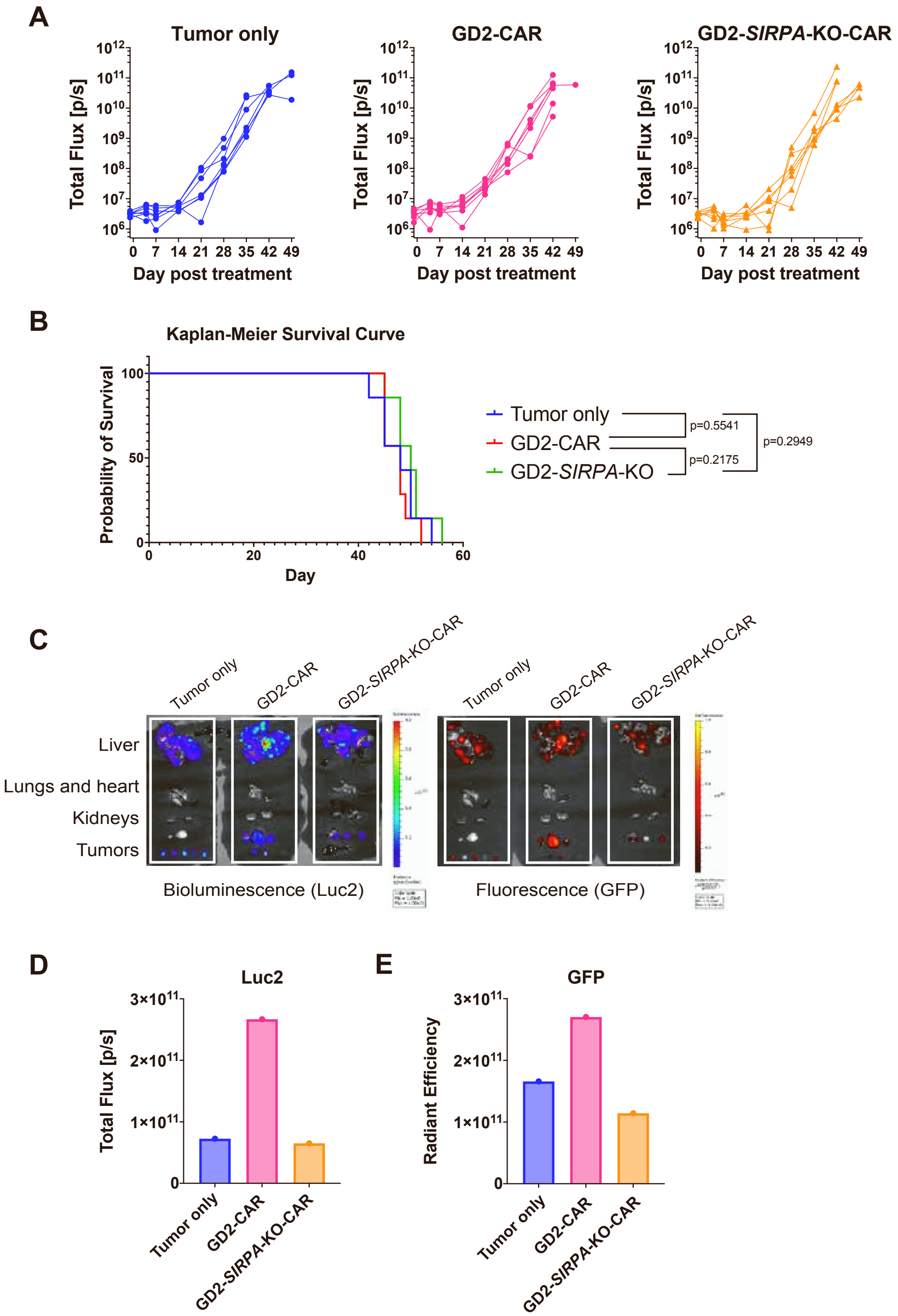
A
Tumor only
GD2-CAR
GD2-SIRPA-KO-CAR
Total Flux [p/s]
Day post treatment
B
Kaplan-Meier Survival Curve
Probability of Survival
Day
Tumor only
GD2-CAR
GD2-SIRPA-KO
p=0.5541
p=0.2175
p=0.2949
C
Liver
Lungs and heart
Kidneys
Tumors
Bioluminescence (Luc2)
Fluorescence (GFP)
D
Luc2
Total Flux [p/s]
E
GFP
Radiant Efficiency

Figure S7. Evaluating GD2-*SIRPA*-KO-CAR iMacs in CHLA-136 xenograft model. (A) Quantification of CHLA-136 tumor xenografts over time for each treatment group, shown as individual mice. NC, negative control. Results are mean total flux [photons/s] ± SEM (n=7). (**B**) Kaplan-Meier Survival Curve of mice from Tumor only, GD2-CAR, and GD2-*SIRPA*-KO-CAR treatment groups using a log-rank test. **(C-E)** Results shown are representative of n=1 mouse from each group. **(C)** Representative images of GFP-Luc2+ CHLA-136 tumor metastasis in NCG-X mice. Organs were isolated upon end of survival on day 48 and imaged using IVIS Spectra and D-Luciferin substrate for bioluminescence (Luc2 expression) and fluorescence (GFP). **(D-E)** Bar graphs of **(D)** Luciferase (Luc2) Total Flux [p/s] and **(E)** GFP Radiant Efficiency of total organs, shown in white ROIs in **(C).**

SUPPLEMENTAL METHODS

SIRPA-KO iPSC PCR sequencing

Partial nanopore PCR sequence alignment of exon 3 of WT and SIRPA KO BM9 iPSCs. Genomic DNA was PCR-amplified, gel-purified, and sequenced using the Oxford Nanopore platform. The KO sequence is identical to WT up to the edited region, followed by a deletion of the downstream sequence.

WT sequence (exon 3)

CCCAGATATGCCAGGCCCCTACCTCTGAGTCTTTGCCCAGGCTGTTCCCTCTGTCTGGAATACC
AGGCTCCCTTCTGGCTCATTCAGATTAGAATTAGCCCAGCTCAGATGTGACTCTTCCAGAGAG
GGTCCAGGCATTCAAACCCACTCTCTGCCATGTCATTTAGCTTTCTGTGCCTCAGTGTCCCCAC
TTAGAATACAGGCTCATGTTGCAGGTTTGTTGTGAGGGTCAAATGAGATGATACATGCACTATA
CTGATCTCACAGCCTGCTTCTGGTGTGCATCCAGTCAATGAACGTCATTGATAAACACTTGAG
GAAACACAGAGGATCACGTAAGGATGAAAAAATGACTGCTTTGTGCTCCTTTCCAGGAGTGG
CGGGTGAGGAGGAGCTGCAGGTGATTCAGCCTGACAAGTCCGTGTTGGTTGCAGCTGGAGA
GACAGCCACTCTGCGCTGCACTGCGACCTCTCTGATCCCTGTGGGGCCCATCCAGTGGTTCAG
AGGAGCTGGACCAGGCCGGGAATTAATCTACAATCAAAAAGAAGGCCACTTCCCCCGGGTAA
CAACTGTTTCAGACCTCACAAAGAGAAACAACATGGACTTTTCCATCCGCATCGGTAACATCA
CCCCAGCAGATGCCGGCACCTACTACTGT

SIRPA-KO sequence (exon 3)

CCCAGATATGCCAGGCCCCTACCTCTGAGTCTTTGCCCAGGCTGTTCCCTCTGTCTGGAATACC
AGGCTCCCTTCTGGCTCATTCAGATTAGAATTAGCCCAGCTCAGATGTGACTCTTCCAGAGAG
GGTCCAGGCATTCAAACCCACTCTCTGCCATGTCATTTAGCTTTCTGTGCCTCAGTGTCCCCAC
TTAGAATACAGGCTCATGTTGCAGGTTTGTTGTGAGGGTCAAATGAGATGATACATGCACTATA
CTGATCTCACAGCCTGCTTCTGGTGTGCATCCAGTCAATGAACGTCATTGATAAACACTTGAG
GAAACACAGAGGATCACGTAAGGATGAAAAAATGACTGCTTTGTGCTCCTTTCCAGG

Cancer cell culture

SK-OV-3 cells were obtained from American Type Culture Collection (ATCC, Manassas, Virginia, U.S.) and sub-cultured according to ATCC recommendations using McCoy's 5A Medium (ATCC, 30-2007) supplemented with 10% Fetal Bovine Serum (FBS; R&D Systems #S12450). WM-266-4 cells were obtained by the Department of Dermatology of the University of Wisconsin-Madison and sub-cultured using McCoy's 5A Medium (ATCC, 30-2007) supplemented with 10% FBS. CHLA-136 cells were

obtained as a gift from the Children's Hospital of Los Angeles and sub-cultured using Iscove's Modified Dulbecco's Medium (IMDM, Gibco, 12200069) supplemented with 10% FBS.

CAR construct and generation of anti-GD2 CAR-iPSCs

GD2-OX40-CD28-ζ CAR sequence was kindly provided by Malcolm Brenner (Baylor College of Medicine),[3] was cloned into an AAVS1-DEST vector (Addgene 80490) and integrated into the AAVS1 locus of the PBMC-3-1 iPSC line using CRISPR-Cas9 as previously described.[4,5]

Flow cytometry

Cells (0.1-1 x 10^6) were resuspended in FACS buffer (PBS with 2% FBS and 0.1% sodium azide) with dilutions of antibodies and incubated at 4°C for 20 – 30 minutes. After washing, flow cytometric analysis was performed on a MACSQuant Analyzer 10 (Miltenyi Biotec). FlowJo software (Version 10, FlowJo LLC) was used for the data analysis. Antibodies used in this study are listed in Table S3.

Phagocytosis assays

iMacs were co-cultured with GFP-expressing cancer cells lines with or without 1µg/mL anti-HER2 or anti-GD3 mAbs, listed in Table S3, in IF9S media, then harvested and stained with anti-CD45 APC antibody (BD, 555483) for flow cytometric analysis. Cells were then washed with FACS buffer for 1-2 times and flow cytometric analysis was performed as above. Engulfment Index was calculated as: (# of DP CD45+GFP+) / (# of total GFP+ cells) x 100.

***In vitro* tumor cell growth assays**

iMacs were co-cultured with GFP- and luciferase-expressing cancer cell lines with or without mAb in respective cancer culture media. To visualize GFP+ cancer cell viability, co-cultures were subject to fluorescence microscopy (ECHO Revolve). For luciferase-based viability, D-luciferin substrate was added to co-cultures and bioluminescence was read at 562nm using a SpectraMax i3X plate reader, Molecular Devices). Untreated cancer cells were used as a spontaneous death control, cancer cells treated with lysis buffer was used as a maximum death control. Relative tumor growth, normalized to 1, was calculated as: (experimental – maximum cell death) / (spontaneous death - maximum cell death).

Serial challenges of iMacs with tumor

iMacs were co-cultured with unmodified or GFP+ Luc2+ cancer cells together in IF9S media (SK-OV-3) or IMDM media + 10% FBS (CHLA-136) at either 10:1 or 20:1 effector-to-target ratios in the presence of anti-HER2 mAb (2µg/mL) for SK-OV-3 or no antibody for CHLA-136. After 24 hours, cultures were assessed for 1) GFP+ tumor viability by fluorescence microscopy and 2) luciferase activity by adding D-luciferin potassium substrate (VivoGlo, Promega, PAP1041) to the co-culture and reading bioluminescence at 562nm on a SpectraMax i3X plate reader. Immediately afterward, the total media of the co-cultures was removed and replenished with 5000 fresh cancer cells and anti-HER2 (2µg /mL) in IF9S media (SK-OV-3) or 5000 fresh cancer cells (CHLA-136). After media replacement, co-cultures were placed back into a normoxic incubator for another 24 hours. This process was repeated three to four times, for a total of four to five tumor exposures over the course of 96 or 120 hours, depending on the specific assay. Expression of FcRs or GD2 CAR was evaluated by flow cytometry. To assess the impact of multiple tumor exposures on phagocytosis, iMacs were serially challenged with unmodified tumor cells four times. iMacs from 96 hour cultures were collected and incubated with GFP-Luc2 cancer cells for 2 hours, labeled with CD45 antibody, and assessed for phagocytosis by flow cytometry. Antigen uptake and processing by iMacs following multiple rounds of tumor exposure was assessed using a self-quenched conjugate of OVA (DQ™ Ovalbumin (D-12053); Molecular Probes) that exhibits fluorescence upon proteolytic degradation. Total iMacs were collected from 96-hour cultures, counted, replated into new 96 well flat-bottom plate and incubated with 100 µg/ml DQ-OVA for 30 mins at 37°C or 4°C (control for background fluorescence) in IF9S media. OVA proteolysis was evaluated by flow cytometry after gating for $CD45^+$ iMacs.

M1/M2 Polarization and Flow Cytometric Analysis

WT and *SIRPA*-KO iMacs were seeded in a 6-well tissue culture plate at 2 million iMacs/mL in IF9S media. For M1 polarization, 20ng/mL IFN-γ (Peprotech, 300-02) and 100ng/mL LPS (Sigma Aldrich, L4391-1MG) were added to the cell culture. For M2 polarization, 20ng/mL IL-4 (Peprotech, 200-04) was added to the cell culture. After 48 hours, M1-stimulated, M2-stimulated, and unstimulated iMacs were collected by incubating with 1X diluted TrypLE (TrypLE Select Enzyme 10X, no phenol red; Thermo Fisher Scientific, A1217701), followed by centrifugation and resuspension in FACS buffer. Cell cultures were then subject to antibody staining and flow cytometric analysis. Antibodies used in this study are listed in Table S3.

Secretome assay

Secretome assay was performed according to the manufacturer's instructions for a Meso Quickplex SQ 120 multiplex cytokine plate reader (U-PLEX assay, MesoScale Discovery).

Western Blot

For Western Blot experiments, iMacs were generated from iPSCs and harvested for analysis. The cells were lysed using Pierce IP lysis buffer with Pierce protease inhibitors (Thermo Fisher Scientific). Protein levels were quantified using the Pierce BCA Assay kit (Thermo Fisher Scientific) and normalized to 10µg of total protein prior to running on pre-cast 4-12% gradient SDS-PAGE gels and subsequent transfer to PVDF membranes using the Bio-Rad Trans-Blot Turbo System. The membrane was blocked with 5% Difco™ Skim Milk (BD, 232100) in TBST buffer (1%; Diluted from 10X TBS Bio-Rad #1706435 and Tween20 Bio-Rad #1610781) for human anti-SIRPα antibody at 55kDa (MyBioSource, MBS2026512, 3µg/mL) and anti-GAPDH at 37kDa (Santa Cruz Biotechnology, 1:5000) for probing. The membranes were incubated with primary antibodies overnight at 4ºC after blocking with mild agitation and were blotted with their corresponding HRP-linked secondary antibodies at room temperature for one hour. Primary and secondary antibodies were diluted in 1% milk TBST buffer. After probing, membranes were washed in 1% TBST buffer for 5 mins three times with mild agitation. Detection of protein on membrane was visualized using Pierce ECL Western Blotting Substrate (Thermo Fisher Scientific).

Immunostaining of pluripotent stem cell markers

The pluripotency markers of WT and *SIRPA*-KO iPSC cells were examined by immunofluorescence staining. The iPSC colonies were fixed with 4% paraformaldehyde (PFA) at room temperature (RT) for 15 minutes after being washed with PBS. The colonies were permeabilized with 0.1 Triton X in PBS for 10 minutes at RT after being washed three times with PBS. Cell colonies were blocked with 5% goat serum at room temperature for one hour after the three washes (1x PBS with 0.2% Tween 20). Following three rounds of washing with 1x PBS containing 0.2% Tween 20, the colonies were incubated for an overnight at 4°C using the primary antibodies OCT3/4 (sc-5279 Santa Cruz Biotechnology), SOX2 (#3579S Cell Signaling Technology), and NANOG (#4903S Cell Signaling Technology) at a 1:200 dilution in 1x PBS containing 1% goat serum. After three washes, the primary antibody was removed, and the colonies were incubated with secondary antibodies, goat anti-mouse IgG Alexa Fluor 488 (#A11001 Thermo Fisher Scientific) for OCT3/4 and goat anti-rabbit IgG Alexa Fluor 594 (#A11012 Thermo Fisher Scientific) for SOX2 and NANOG, at a dilution of 1:5000 in 1x PBS containing 1% goat serum for one hour at RT and put into the platform rocker. As a nuclear stain control, the colonies were stained with Hoechst #33342 (Thermo Fisher Scientific) at 1:1000 in 1x PBS for 10 minutes in the dark after three washes. Finally, a Nikon Eclipse Ti2 widefield fluorescent microscope was used to take pictures of the colonies after they had been washed three times with 1x PBS containing 0.2% Tween 20.

Immunostaining of SIRPα

For immunofluorescence experiments, GD2 CAR iMacs and GD2-*SIRPA*-KO CAR iMacs were generated from iPSCs and harvested for analysis. iMacs were then fixed in cold methanol for 10 mins at -20°C and then blocked and permeabilized with 2.5% donkey serum (Sigma-Aldrich D9663) and 0.2% Triton-X100

(Made from 100% Triton X-100; Sigma Aldrich X100) for 20 mins at room temperature. Human anti-SIRPα (MyBioSource, MBS2026512, 3μg/mL) was added to the iMacs and incubated for 2 hours at room temperature. Primary antibody was removed, and a secondary anti-Rabbit IgG Alexa Fluor 488 (Invitrogen, A-21206, 2μg/mL) was applied and incubated for 1 hour at room temperature. In between staining steps, iMacs were washed with phosphate buffered saline (PBS). DAPI was used as a nuclear stain control. Images were taken by Nikon eclipse Ti confocal microscope, cropped from original 20x images.

Generation and validation of *SIRPA*-KO-*AAVS1*-*SIRPA*-Knock-in (KI) hiPSC line
The AAVS1 donor plasmid (derived from Addgene #22075) is linearized at the cloning site, downstream of CAG promoter (Figure 2I). To obtain *SIRPA* complementary DNA (cDNA) from cells, high-quality RNA was isolated (Invitrogen, #2910133) from WT (BM9) iMacs, removing genomic DNA contamination, and synthesizing cDNA through reverse transcription (Qiagen #178027558). A small sequence overlapped with each end of the cloning site is added onto the *SIRPA* gene through PCR. The insert and the linearized vector, with overlapped sequences of 15 bp – 20 bp on both 5'- and 3'-end, respectively, are mixed in an appropriate ratio and incubated with recombinase Exnase (ABP Biosciences, #D017-02) at 50°C for 5 – 15 mins according to manufacturer's instructions. The product was transformed into competent cells, purified (IBI Scientific, #IB47171) and verified through sequencing (FASTA sequence attached). The AAVS1-CAG-*SIRPA* plasmid, sgRNA (GGGGCCACTAGGGACAGGAT) and Cas9 protein (PNA Bio #CP02) were co-transfected into singularized *SIRPA*-KO iPSCs using Lonza Amaxa and Human Stem Cell Nucleofector Starter Kit (Lonza, VPH-5002). Cells were selected with puromycin (0.5 mg/mL, Sigma) for a maximum of 5 days and resistant clones were screened by PCR and Western Blot experiment.

RNA Sequencing
WT and *SIRPA*-KO iMacs were either cultured alone for 24 hours, or co-cultured with SK-OV-3 cells (E:T = 10:1) and anti-HER2 (1μg/mL) for 24 hours or 96 hours. All cells were harvested and sorted for CD45+ cells using anti-human CD45 antibody and anti-CD45 microbeads (Miltenyi, MACS sorter). Total RNA was isolated from the CD45+ sorted populations by using the RNeasy Mini Kit (Qiagen, 74104) and quantified with TapeStation (GENEWIZ by Azenta). The RNA samples were rRNA depleted using QIAGEN FastSelect rRNA HMR Kit (Qiagen, Germantown, MD, USA). RNA sequencing was performed at Azenta Life Sciences. The kit NEBNext® Ultra™ II RNA Library Prep Kit for Illumina® (New England Biolabs, Ipswich, MA, USA) was used for library preparation following manufacture instructions. For each sample ~20 million paired end reads per sample were sequenced on Illumina NovaSeq Xplus.

Bioinformatic analysis of RNA-sequencing data
Bioinformatic analysis of transcriptomic data adhered to recommended ENCODE guidelines and best practices for RNA-Seq.[6] Alignment of adapter-trimmed (Skewer v0.1.123)[7] 2x150 (paired-end; PE) bp strand-specific Illumina reads to the *Homo sapiens* GRCh38.p10 genome (assembly accession NCBI:GCA_000001405.25) was achieved with the Spliced Transcripts Alignment to a Reference (STAR v2.7.10b) software,[8] a splice-junction aware aligner, using annotation provided by Ensembl. Expression estimation was performed with RSEM v1.3.1 (RNASeq by Expectation Maximization).[9] To test for differential gene expression among individual group contrasts, expected read counts obtained from RSEM were used as input into edgeR (3.42.2).[10] Inter-sample normalization was achieved with the trimmed mean of M-values (TMM)[11] method. Statistical significance of the negative-binomial regression test was adjusted with a Benjamini-Hochberg false discovery rate (FDR) correction at the 5% level.[12] Prior to statistical analysis with edgeR, independent filtering was applied and required genes to have a count-per-million (CPM) above k in n samples, where k is determined by minimum read count (10 reads) and by the sample library sizes where n is determined by the number of biological replicates in each group. The validity of the Benjamini-Hochberg FDR multiple testing procedure was evaluated by inspection of the uncorrected p-value distribution. Gene set enrichment analysis (GSEA) was performed following criteria outlined by Subramanian et al., 2005.[13]

Table S1. Medium components in IF9S media.

Medium Components	Day 0-2	Day 2-4	Day 4-9	Day 9-15	Day 15-19	Vendor	Catalog #
IF9S*	x	x	x	x	x	N/A	N/A
FGF2 (50ng/mL)	x	x	x			Peprotech	100-18B
BMP4 (50ng/mL)	x					Peprotech	120-05
Activin A (15ng/mL)	x					Peprotech	120-14E
LiCl (2mM)	x					Sigma-Aldrich	203637-10G
Y-27632 (10µM)	x					Tocris	1254
VEGF (50ng/mL)		x	x			Peprotech	100-20
SB431542 (5µM)		x				Biogems	3014193
SCF (50ng/mL)			x			Peprotech	300-07
TPO (50ng/mL)			x			Peprotech	300-18
IL-3 (10ng/mL)			x	x		Peprotech	200-03
IL-6 (50ng/mL)			x	x		Peprotech	200-06
M-CSF (80ng/mL)				x	x	Peprotech	300-25

*IF9S medium components are previously defined by Uenishi et al., 2014.[14]

Table S2. PCR primers and synthetic guide (sg) RNAs used in this study.

Name	Primer or sgRNA	Sequence
SIRPA intron 2_F	primer	AATCTTAACACCTTGTACAGCCCCA
SIRPA exon 3_R	primer	AGTGCCTGCTCCAGACTTAAA
SIRPA sgRNA_1	sgRNA	GTGCTCCTTTCCAGGAGTGG
SIRPA sgRNA_2	sgRNA	ACTTAAACTCCACGTCATCG

Table S3. Antibodies used in this study.

Antibody Target	Conjugate	Vendor	Catalog #	Application
CD11b	APC	Miltenyi	130-091-241	Flow cytometry
CD11b	PE-Cy5	BD Biosciences	555389	Flow cytometry
CD14	PE	BD Biosciences	555398	Flow cytometry
CD16	PE	BD Biosciences	555407	Flow cytometry
CD18	APC	BD	551060	Flow cytometry
CD32	APC	BD Biosciences	559769	Flow cytometry
CD41a	PE	BD Biosciences	555467	Flow cytometry
CD43	FITC	BD Biosciences	555475	Flow cytometry
CD45	APC	BD Biosciences	555485	Flow cytometry
CD45	BV421	BD Biosciences	563880	Flow cytometry
CD47	APC	Biolegend	323123	Flow cytometry
CD47	PE	Biolegend	323108	Flow cytometry
CD64	FITC	BD Biosciences	555527	Flow cytometry
CD80	PE-Cy5	BD Biosciences	559370	Flow cytometry
CD86	APC	Miltenyi	130-116-161	Flow cytometry
CD163	BV421	Biolegend	333610	Flow cytometry
CD206	FITC	Biolegend	321104	Flow cytometry
CD235a	PE	BD Biosciences	561051	Flow cytometry
HLA-DR	APC	BD Biosciences	559868	Flow cytometry
Anti-14G2a [1A7] *	APC	NCI Repository	NA	Flow cytometry
Anti-GD3 (R24)	Unconjugated	NCI Repository	NA	Functional studies
Anti-GD2 (Ch14.18)	Unconjugated	NCI Repository	NA	Functional studies
Herceptin (anti-HER2)	Unconjugated	Genetech, Inc	NA	Functional studies
SIRPα	Unconjugated	MyBioSource	MBS2026512	Western blot

*Anti-14G2a [1A7] APC-conjugated antibody was generated in house using unconjugated Anti-14G2a [1A7] antibody and the Abcam APC Conjugation Kit – Lightning Link #ab201807

REFERENCES

1. Hatherley, D., Graham, S.C., Turner, J., Harlos, K., Stuart, D.I., and Barclay, A.N. (2008). Paired Receptor Specificity Explained by Structures of Signal Regulatory Proteins Alone and Complexed with CD47. Molecular Cell *31*, 266–277. https://doi.org/10.1016/j.molcel.2008.05.026.

2. Hatherley, D., Harlos, K., Dunlop, D.C., Stuart, D.I., and Barclay, A.N. (2007). The Structure of the Macrophage Signal Regulatory Protein α (SIRPα) Inhibitory Receptor Reveals a Binding Face Reminiscent of That Used by T Cell Receptors*. Journal of Biological Chemistry *282*, 14567–14575. https://doi.org/10.1074/jbc.M611511200.

3. Louis, C.U., Savoldo, B., Dotti, G., Pule, M., Yvon, E., Myers, G.D., Rossig, C., Russell, H.V., Diouf, O., Liu, E., et al. (2011). Antitumor activity and long-term fate of chimeric antigen receptor-positive T cells in patients with neuroblastoma. Blood *118*, 6050–6056. https://doi.org/10.1182/blood-2011-05-354449.

4. Oceguera-Yanez, F., Kim, S.-I., Matsumoto, T., Tan, G.W., Xiang, L., Hatani, T., Kondo, T., Ikeya, M., Yoshida, Y., Inoue, H., et al. (2016). Engineering the AAVS1 locus for consistent and scalable transgene expression in human iPSCs and their differentiated derivatives. Methods *101*, 43–55. https://doi.org/10.1016/j.ymeth.2015.12.012.

5. Zhang, J., Webster, S., Duffin, B., Bernstein, M.N., Steill, J., Swanson, S., Forsberg, M.H., Bolin, J., Brown, M.E., Majumder, A., et al. (2023). Generation of anti-GD2 CAR macrophages from human pluripotent stem cells for cancer immunotherapies. Stem Cell Reports *18*, 585–596. https://doi.org/10.1016/j.stemcr.2022.12.012.

6. Hitz, B.C., Rowe, L.D., Podduturi, N.R., Glick, D.I., Baymuradov, U.K., Malladi, V.S., Chan, E.T., Davidson, J.M., Gabdank, I., Narayanan, A.K., et al. (2016). SnoVault and encodeD: A novel object-based storage system and applications to ENCODE metadata. Preprint, https://doi.org/10.1101/044578 https://doi.org/10.1101/044578.

7. Jiang, H., Lei, R., Ding, S.-W., and Zhu, S. (2014). Skewer: a fast and accurate adapter trimmer for next-generation sequencing paired-end reads. BMC Bioinformatics *15*, 182. https://doi.org/10.1186/1471-2105-15-182.

8. Dobin, A., Davis, C.A., Schlesinger, F., Drenkow, J., Zaleski, C., Jha, S., Batut, P., Chaisson, M., and Gingeras, T.R. (2013). STAR: ultrafast universal RNA-seq aligner. Bioinformatics *29*, 15–21. https://doi.org/10.1093/bioinformatics/bts635.

9. Li, B., and Dewey, C.N. (2011). RSEM: accurate transcript quantification from RNA-Seq data with or without a reference genome. BMC Bioinformatics *12*, 323. https://doi.org/10.1186/1471-2105-12-323.

10. Robinson, M.D., McCarthy, D.J., and Smyth, G.K. (2010). edgeR: a Bioconductor package for differential expression analysis of digital gene expression data. Bioinformatics *26*, 139–140. https://doi.org/10.1093/bioinformatics/btp616.

11. Robinson, M.D., and Oshlack, A. (2010). A scaling normalization method for differential expression analysis of RNA-seq data. Genome Biology *11*, R25. https://doi.org/10.1186/gb-2010-11-3-r25.

12. Reiner, A., Yekutieli, D., and Benjamini, Y. (2003). Identifying differentially expressed genes using false discovery rate controlling procedures. Bioinformatics *19*, 368–375. https://doi.org/10.1093/bioinformatics/btf877.

13. Subramanian, A., Tamayo, P., Mootha, V.K., Mukherjee, S., Ebert, B.L., Gillette, M.A., Paulovich, A., Pomeroy, S.L., Golub, T.R., Lander, E.S., et al. (2005). Gene set enrichment analysis: A knowledge-based approach for interpreting genome-wide expression profiles. Proceedings of the National Academy of Sciences *102*, 15545–15550. https://doi.org/10.1073/pnas.0506580102.

14. Uenishi, G., Theisen, D., Lee, J.-H., Kumar, A., Raymond, M., Vodyanik, M., Swanson, S., Stewart, R., Thomson, J., and Slukvin, I. (2014). Tenascin C Promotes Hematoendothelial Development and T Lymphoid Commitment from Human Pluripotent Stem Cells in Chemically Defined Conditions. Stem Cell Reports *3*, 1073–1084. https://doi.org/10.1016/j.stemcr.2014.09.014.
